# Rome in its setting. Post-glacial aggradation history of the Tiber River alluvial deposits and tectonic origin of the Tiber Island

**DOI:** 10.1371/journal.pone.0194838

**Published:** 2018-03-28

**Authors:** Fabrizio Marra, Laura Motta, Andrea L. Brock, Patrizia Macrì, Fabio Florindo, Laura Sadori, Nicola Terrenato

**Affiliations:** 1 Istituto Nazionale di Geofisica e Vulcanologia, Rome, Italy; 2 Kelsey Museum of Archaeology, University of Michigan, Ann Arbor, Michigan, United States of America; 3 Department of Classical Studies, University of Michigan, Ann Arbor, Michigan, United States of America; 4 Dipartimento di Biologia Ambientale, Universita di Roma “La Sapienza”, Roma, Italy; Max Planck Institute for the Science of Human History, GERMANY

## Abstract

The Tiber valley is a prominent feature in the landscape of ancient Rome and an important element for understanding its urban development. However, little is known about the city’s original setting. Our research provides new data on the Holocene sedimentary history and human-environment interactions in the Forum Boarium, the location of the earliest harbor of the city. Since the Last Glacial Maximum, when the fluvial valley was incised to a depth of tens of meters below the present sea level, ^14^C and ceramic ages coupled with paleomagnetic analysis show the occurrence of three distinct aggradational phases until the establishment of a relatively stable alluvial plain at 6–8 m a.s.l. during the late 3^rd^ century BCE. Moreover, we report evidence of a sudden and anomalous increase in sedimentation rate around 2600 yr BP, leading to the deposition of a 4-6m thick package of alluvial deposits in approximately one century. We discuss this datum in the light of possible tectonic activity along a morpho-structural lineament, revealed by the digital elevation model of this area, crossing the Forum Boarium and aligned with the Tiber Island. We formulate the hypothesis that fault displacement along this structural lineament may be responsible for the sudden collapse of the investigated area, which provided new space for the observed unusually large accumulation of sediments. We also posit that, as a consequence of the diversion of the Tiber course and the loss in capacity of transport by the river, this faulting activity triggered the origin of the Tiber Island.

## Introduction

**Fig 1:**
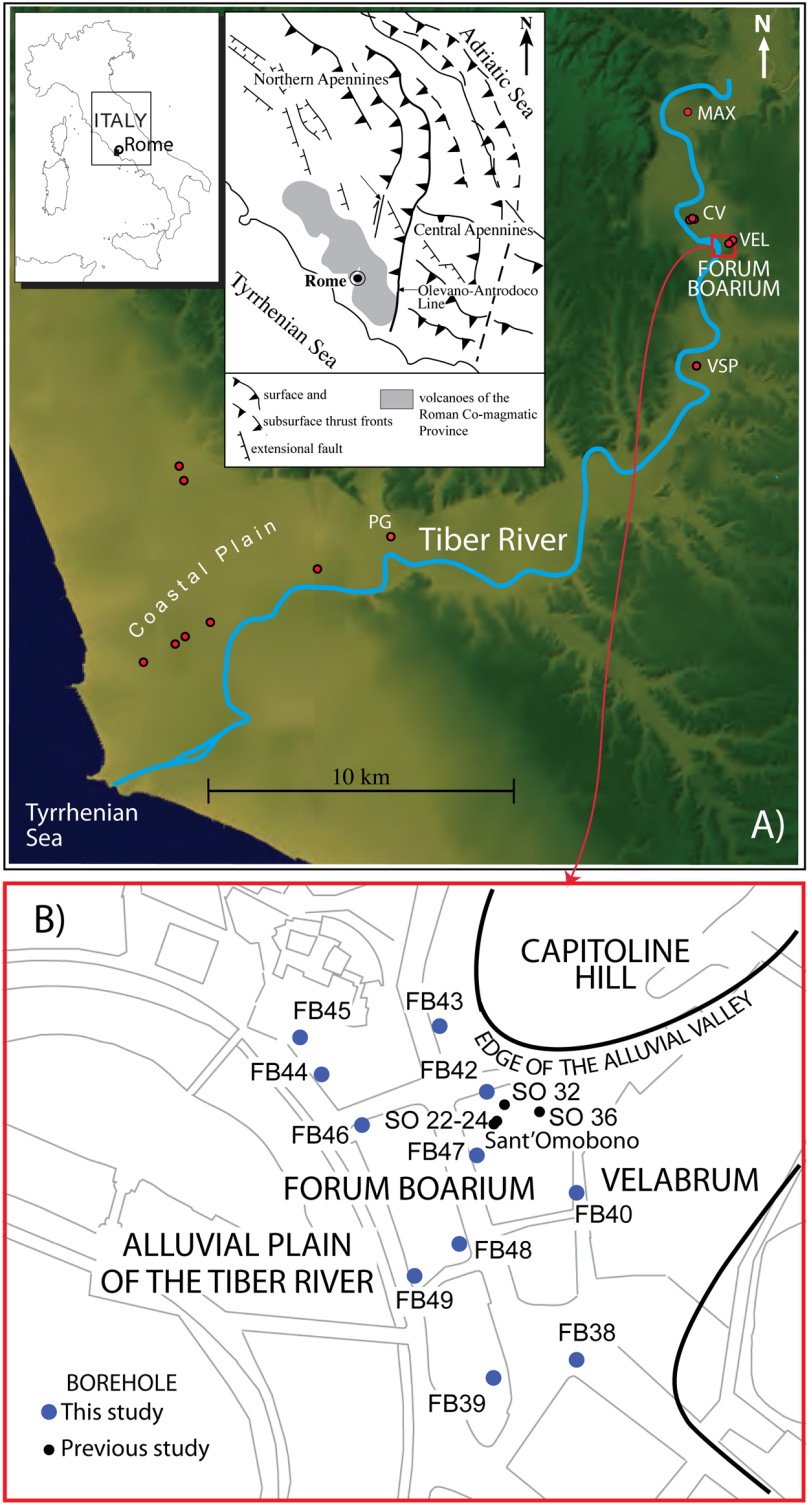
(*A*) Digital Elevation Model (DEM) image TINITALY/01 square WA 6570, used with permission of the Istituto Nazionale di Geofisica e Vulcanologia, Rome (S4 File), of the area of Rome showing location in the Tiber river valley and in the coastal plain of cores from previous literature (red dots) employed in the reconstruction of the aggradational history of the river sediments performed in this paper. PG: Ponte Galera; VSP: Varco San Paolo; VEL: Velabrum; CV: Corso Vittorio; MAX: Maxxi. (*B*) Sketch map of the investigated area in the Forum Boarium, showing location of the boreholes (blue dots) performed for the present study.

Despite Rome’s prominent position in the development of Mediterranean civilization, scholars actually know frustratingly little about the city’s original setting and early history. The present cross-disciplinary investigation of Rome’s river valley has produced new data on the Holocene evolution of the landscape in the heart of the ancient city. This area, known as the Forum Boarium, is the location of Rome’s first river harbor (Fig 1). Our work developed out of geoarchaeological investigations in the context of the Sant’Omobono Project (https://sites.lsa.umich.edu/omobono/), which led to the discovery of settlement evidence during the Late Bronze Age, as well as the remains of an early 6^th^ century BCE temple—the earliest known from Rome. To reconstruct details of the pre-urban landscape, a combination of deep-trench excavation, coring survey, environmental sampling and paleomagnetic analysis has allowed us to explore the archaeological and alluvial sequence of the Tiber River Valley to a depth of 55 m below the modern surface. Here, we present the results of our investigation of the river sedimentary history in this sector since the end of the Last Glacial Maximum, when the fluvial valley was incised to a depth of tens of meters below the present sea level, until the establishment of a relatively stable alluvial plain at 6–8 m a.s.l. during the late 3^rd^ century BCE. This new reconstruction provides a starkly different context for the crucial period during which Rome was transformed from a hut settlement to a monumental city.

The aggradational history of the Tiber River in the 30 km long valley comprised between the city of Rome and the coastal plain is strictly coupled with the post-glacial sea-level rise [1,2]. Geochronological constraints provided to sediment aggradation demonstrate abrupt shifts in the capacity of transport by the Tiber River, both related with the major climatic events (i.e., glacial terminations) as well as minor ones [1,2]. In the present study we have significantly increased the detail upon the Holocene interval by providing 33 new ^14^C age determinations spanning 13500–2250 calibrated years before present (cal yr BP), and an additional 61 age determinations on ceramic fragments incorporated in the sedimentary succession, spanning the 8^th^ through the 2^nd^ century BCE.

## Materials and methods

### Permits

All necessary permits were obtained for the described study, which complied with all relevant regulations.

### Chronostratigraphic investigations

We analyzed sediments from 11 boreholes drilled in the Forum Boarium area, between the Capitoline Hill and the Palatine Hill (see Fig 1 for location; geographical coordinates are provided in Table 1). Visual lithostratigraphic analysis has been performed on more than 230 m of natural and anthropic deposits. Moreover, we have included data from 4 additional boreholes (SO 22-24-32-36) previously drilled in the area of the Sant’Omobono temple [3–5]. Sediments and archaeological deposits have been sampled and screened for organic and cultural material in order to provide chronological constraints for the different stratigraphic units. In addition, we performed paleomagnetic investigations on clay sections between +2 and +6 m a.s.l. in cores FB40-43-47, as well as between -2.5 and +1 m a.s.l. in FB38.

**Table 1:** Geographical coordinates of the boreholes performed for the present study.

CORE	LATITUDE	LONGITUDE
FB38	41° 53' 19.79"N	12° 28' 55.21"E
FB39	41° 53' 19.34"N	12° 28' 52.20"E
FB40	41° 53' 24.64"N	12° 28' 55.05"E
FB42	41° 53' 27.24"N	12° 28' 51.55"E
FB43	41° 53' 28.96"N	12° 28' 49.83"E
FB44	41° 53' 27.55"N	12° 28' 46.56"E
FB45	41° 53' 28.60"N	12° 28' 44.13"E
FB46	41° 53' 26.21"N	12° 28' 47.31"E
FB47	41° 53' 25.50"N	12° 28' 51.30"E
FB48	41° 53' 22.98"N	12° 28' 50.83"E
FB49	41° 53' 22.33"N	12° 28' 49.00"E

### Cores

The boreholes were drilled with variable depth from 15 to 53 m below the ground surface in the investigated area (Fig 1). Continuous recover of the sediment was provided by a Beretta model T46 hydraulic drilling rig using a 3 m long corer with inner diameter of 97 mm.

Visual lithostratigraphic and in situ geotechnical (i.e. pocket penetrometer test) analyses of the cores was performed in order to describe the characteristics of the recovered sediments, including texture, granulometry, coherence, color, mineralization (e.g., oxydation, carbonatic concretion), presence of vegetal, faunal and anthropic material.

### Chronology

Chronological constraints were determined by a combination of ^14^C and archaeological dating. Radiocarbon dating, including the treatment of the bulk sediments to retrieve plant material in FB48, was performed by Beta Analytics (Miami, FL); full analytical data are provided in SI dataset 1. Laura Sadori processed the palynological samples from FB38 while the ^14^C dates on pollen remains were provided by Center for Isotopic Research on Cultural and Environmental Heritage at the Seconda Università di Napoli (Caserta, Italy). ^14^C dating on a piece of wood from core FB38-16 was performed by the Centre for Isotope Research at Groningen (NL).

All dates have been converted to calendar ages BCE and BP (2 sigma range) relative to 1950 using Cal13 dataset [6].

When visible we collected organic and cultural remains from the identified discrete lithostratigraphic units. In addition, the sediment was sampled for wet sieving in selected cores at 10 cm interval in order to produce supplementary dating material.

**Fig 2:**
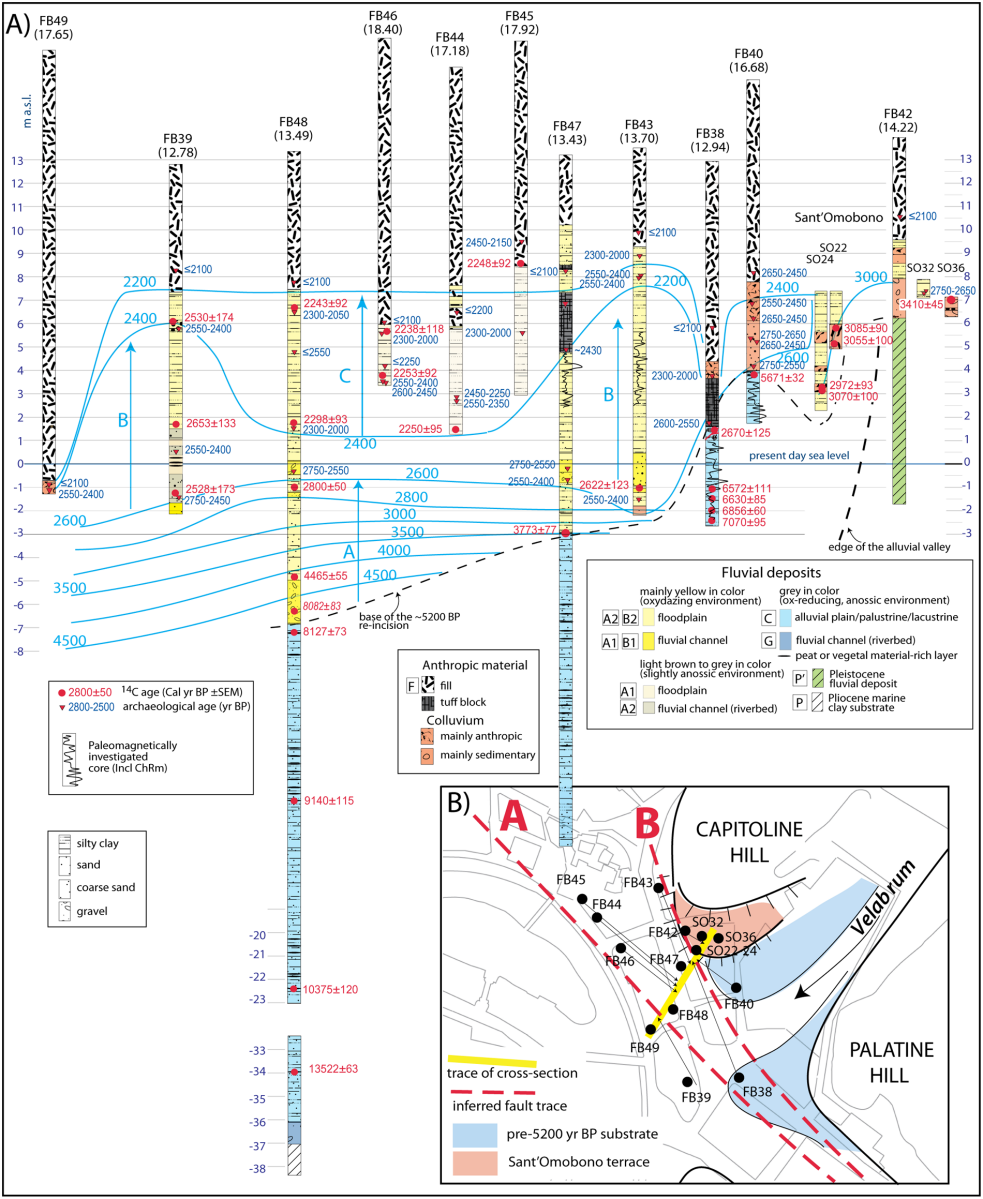
(*A*) Composite cross-section along a principal transect perpendicular to the alluvial valley of the Tiber River (yellow trace in inset b), showing stratigraphic logs of the 11 boreholes performed for this study in the Forum Boarium and of 4 boreholes previously drilled in the Sant’Omobono area [4], with selected ^14^C and ceramic age constraints to sediment aggradation. Additional details on chronology are shown in S2 File. Blue lines are the tentative isochrone lines, defining three phases of sediment aggradation (arrows A, B, C) in the time span 5200–2200 yr BP. (*B*) Map showing location of the boreholes and the trace of the hypothesized faults.

Some of the ^14^C dates (listed in S2 File) have been rejected as non-reliable, based on inconsistency with archaeological dates and/or general stratigraphy, while others produced a date range too wide to be useful, falling within the so called Hallstatt Plateau—a plane on the calibration curve that causes radiocarbon dates in the middle of the 1^st^ millennium BCE to convert to a calendar age ranging several hundred years. The ceramic ages in Fig 2 result from a selection from the total amount of analyses, after discarding those ceramic fragments yielding inconsistent stratigraphic position with respect to lower younger ages, which were interpreted as reworked material. (see S1 File for full methods).

#### Paleomagnetic sampling, laboratory procedures, and analysis

High-resolution palaeomagnetic investigations were performed on cores FB38, 40, 43 and 47, sampled using standard ~8 cm^3^ plastic cubes oriented with respect to the vertical. A total of 198 oriented discrete samples were collected at ca. 5-cm spacing from the center of the three half sections. To minimize sample dehydration and alteration, samples were packed in sealed bags and stored in a refrigerated room until they were processed at the Istituto Nazionale di Geofisica e Vulcanologia, Rome.

Natural and artificial magnetizations were measured at room temperature using a narrow-access pass through 2-G Enterprises cryogenic magnetometer housed in a Lodestar Magnetics shielded room. The natural remanent magnetization (NRM) was stepwise AF demagnetized in 12 steps at successive peak fields of 5, 10, 15, 20, 25, 30, 40, 50, 60, 80, 90 and 100 mT.

Principal component analysis [7] was used to calculate characteristic remanent magnetization (ChRM) directions, with linear best fits calculated from a minimum of three demagnetization steps using the PuffinPlot paleomagnetic analysis application [8]. The maximum angular deviation (MAD) was calculated to provide an estimate of the precision related to each best-fit line. Down-core variations in the inclinations of ChRM is primarily used for comparison and lateral correlation among the sedimentary successions, in consultation with ages and information from radiocarbon and archaeological samples.

In order to demonstrate that the ChRM record is not due to complexities and variations that arise from the magnetic mineralogy of these sediments, a set of rock magnetic analyses, based on magnetic coercivity and thermal unblocking characteristics, were conducted on representative discrete samples (Full paleomagnetic data in S1 File).

### Geologic and geomorphologic setting of the city of Rome

The city of Rome is located on the central Tyrrhenian Sea margin of Italy, where the principal NW-SE structural trend of the Apennines mountain range is interrupted by the N-S oriented external thrust fronts of the northern orogenic arc (Olevano-Antrodoco line, inset in Fig 1). A NW-SE belt of potassic volcanoes developed during Middle Pleistocene along the Tyrrhenian Sea margin (Roman Co-magmatic Province; [9]), parallel to the major extensional faults originated by back-arc extension that induced the opening of the Tyrrhenian Sea Basin [10]. Two major phases of regional uplift associated with volcanism occurred around 800 kyr BP and 250 kyr BP through the Present, causing a paleogeographic change in the area of Rome [11]. This area was characterized by marine sedimentary conditions during Pliocene through Early Pleistocene times; thereafter, a paleo-Tiber delta developed since ca. 800 kyr BP [12]. During Middle-Late Pleistocene and Holocene times the sedimentary processes in the area of Rome were restricted in the fluvial channels and coastal plain and were strongly controlled by sea-level changes linked to glacio-eustatism ([13] and references therein). A well defined hydrographic network drained by the Tiber River is present in the area of Rome (see Fig 1), where the Late Pleistocene-Holocene alluvial valleys display prominent and steep banks bordering the floodplains. The regional uplift of ~ 50 m that occurred in the last 250 kyr was the concomitant cause for rejuvenating and sharpening the morphological features of this hydrographic network. At each glacial maximum in the last two marine isotopic stages 8 and 6, the valleys were excavated deeper than during the previous glacial sea-level falls, and the subsequent sea-level rises associated with the high-stands could account only for partial filling of the incisions originated during the erosive phases [14]. These marked features of the fluvial valleys are partially obliterated in the urban area, where more than 2,000 years of anthropic activity strongly modified the original morphology.

Structural control on the recent hydrographic network of the Tiber River due to active tectonics, acting mainly through vertical movement, has been suggested based on geomorphologic evidence [15], while fault displacement affecting the Holocene alluvial sediments of the Tiber River has been documented in the coastal plain [16]. The occurrence of tectonic deformation in the area of Rome in the historical era has been documented also in the Acque Albule basin near Tivoli, where fault displacement affects an aqueduct system serving a 2^nd^-3^rd^ century CE Roman villa [17]. It remains debated whether the diffuse evidence of tectonic activity should be related to seismic activity, or whether fault displacements should be interpreted as aseismic creep [18–20], due to the lack of strong earthquakes that are attributed to local sources in the historical record for Rome [21].

## Results

### Chronology

The chronological framework of this study is drawn from a combination of radiocarbon and archaeological samples collected from the Forum Boarium coring survey and two additional samples collected in the Maxxi borehole [1] previously performed in the Tiber Valley north of the Forum Boarium (Fig 1). Ages and information for all the radiocarbon and archaeological samples dated in the present work are provided in S2 File. Full ^14^C analytical data are provided in S3 File. Geochronological constraints presented here (Fig 2) have been compared and integrated in the stratigraphic reconstructions with the few others provided to the Tiber River sedimentary succession in previous work [1,2,22–24].

**Fig 3:**
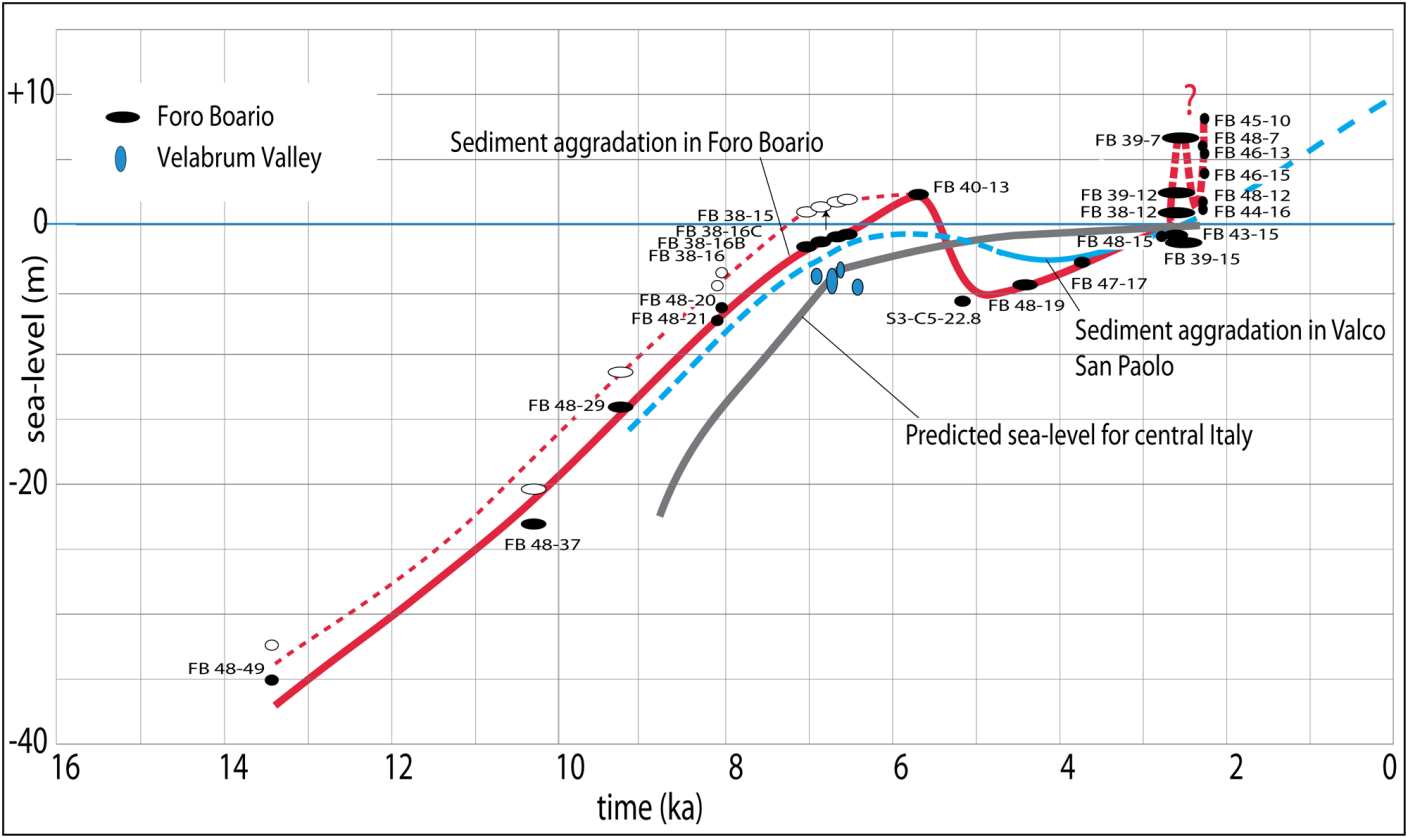
^14^C age vs depth model for the dated samples (black circles: width includes age errors). Data for the Velabrum Valley [23] are also shown (blue circles). Aggradation within the Tiber Valley in Forum Boarium (red solid line) is compared to the equivalent curve reconstructed in Valco San Paolo (VSP, Fig 1 for location) [2] (blue line), and to the curve of the predicted sea-level for central Italy [25]. A possible 3 m tectonic displacement (see text) lowering the stratigraphic column at FB38 with respect to FB40 is considered and amended by shifting upward for an equivalent amount the age data for FB38 (open symbols); the corresponding sediment aggradation curve is represented by the dashed red line.

The ^14^C age vs depth diagram shows a substantially steady, linear trend from 14000 through 7000 yr BP (solid red curve in Fig 3), in agreement with the fast recovery of the sea-level in this time span. In particular, the Tiber River aggradation curve always remains above the sea-level (gray curve in Fig 3), consistent with the continental feature of the alluvial sediments in the Forum Boarium area. The difference in elevation is progressively reduced, according to the establishment of estuarine conditions in the present delta since around 6000 yr BP [2,22]. Also, according to the general trend of the sea-level rise, an initial sub-horizontal curve characterizes the time span 7000–6500 yr BP. However, an inversion of this trend, with an anomalous rise of the aggradation line between 6500 and 5500 yr BP, is observed in the Forum Boarium.

In addition, a marked fall below the predicted sea-level for the corresponding age (gray curve) is observed between 5671±32 and 4465±55 cal yr BP, consistent with the inversion in the trend of the aggradation curve occurring in the southern tract of the Tiber River Valley in Rome (Valco San Paolo) and in the coastal plain [2] (blue curve in Fig 3). Rather than interpreted as a result of an equivalent sea-level fall, such low elevation of the sediment-aggradation curve has been partially explained by erosion and sediment compaction [2]. Independent from the original absolute elevation, data from Forum Boarium indicate a new progressive increase of the base level within the Tiber River alluvial plain from 4465±55cal yr BP through 2800±50 cal yr BP, and a sudden jump of several meters around 2600 yr BP (thick dashed red line in Fig 3).

For the period 700 BCE-400 BCE (known as ^14^C Hallstatt Plateau in archaeology, see Methods) ^14^C ages have been supplemented by dated cultural material which offers a more nuanced and reliable chronology. This has allowed us to check inconsistencies in the geochronological sequence and to recognize some apparent contradictory results due to the re-deposition of older material. Moreover, the introduction of mortar, a Roman invention of the mid-2^nd^ century BCE [26,27], defines a major cultural horizon in the boreholes, while well-documented building phases of the Sant’Omobono temple provide markers in the early 6^th^ and early 5^th^ century BCE [28]. Conversion in years before present (BP) of the archaeological ages, determined on ceramic fragments are reported in S2 File. Together the archaeological and radiocarbon dates support a robust chronological framework for the investigated period of deposition.

### Paleomagnetism

Clay sections from four boreholes have been analyzed to obtain records of the past geomagnetic field and downcore variations of rock magnetic parameters to be used for comparison and lateral correlation among the sedimentary successions. The investigated portions of cores FB47 and FB43 were expected to correspond to different depositional phases with respect to FB38 and FB40. The paleomagnetic analysis was intended to provide a characteristic profile to each interval, based on the selected magnetic parameters (SI Text) that could be compared with each other. In particular, besides lateral correlation between the sedimentary successions occurring in FB43-FB47 and in FB38-FB40, analyses were aimed at checking the possible coeval age of the two lacustrine clay sections occurring at different elevations in FB38 and FB40.

**Fig 4:**
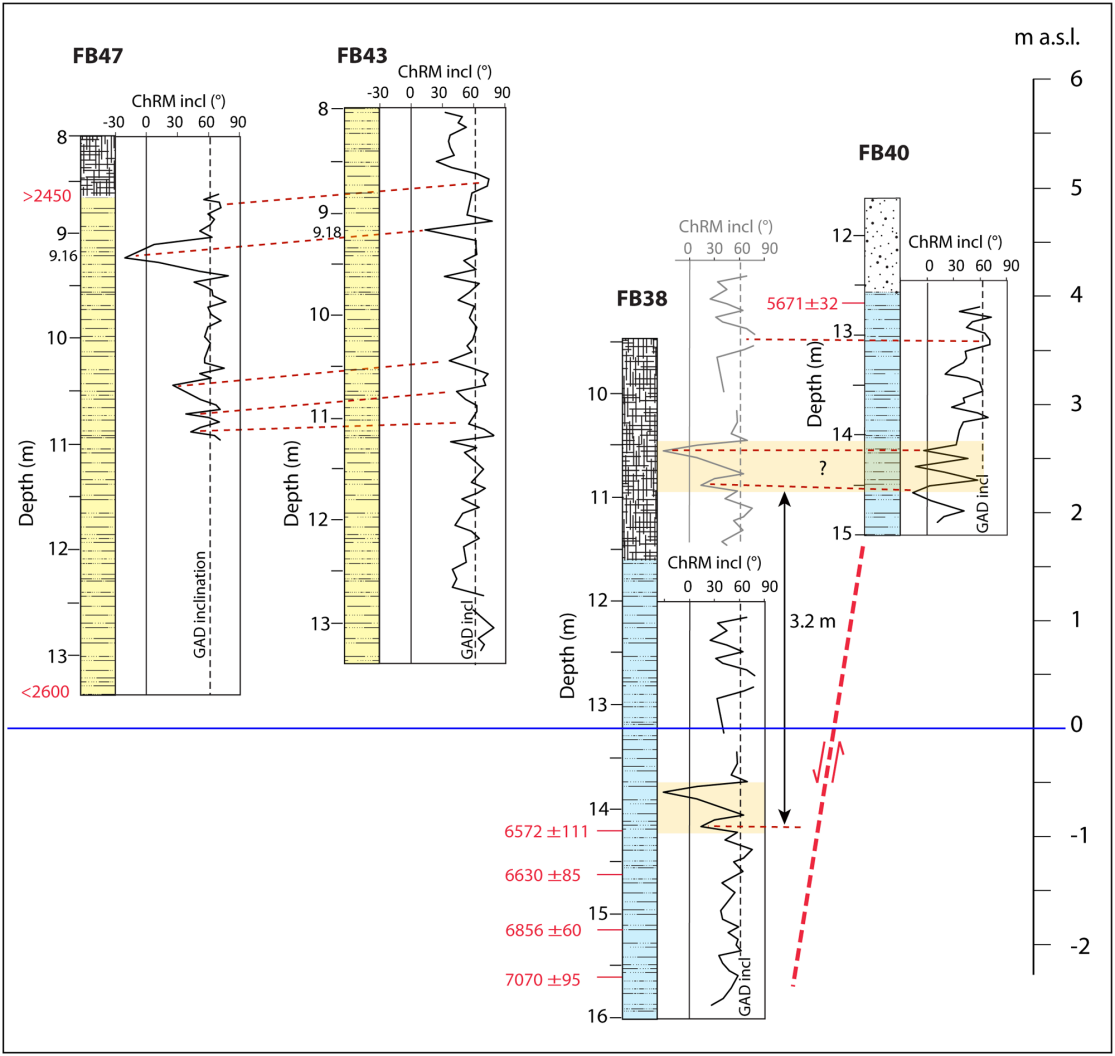
Comparison of the curves of the magnetic inclination (ChRM incl) measured in cores FB 47-43-40-38 (Figure B in S1 File for full paleomagnetic data). Dashed horizontal red lines mark the inferred correlation between the curves measured at FB47 and FB43. Possible correlation is also highlighted between FB38 and FB40 (colored area), after FB 38 is translated 3.2 m upwards to restore the hypothesized fault displacement.

The sediments generally give stable univectorial paleomagnetic directions after removal of a low-coercivity overprint during demagnetization with peak AF of 10 mT. Relatively low coercivities, as evidenced by the median destructive field (MDF_NRM_) parameter imply that magnetite (and/or low-Ti titanomagnetite) is the primary remanence carrier. Characteristic remanence magnetization (ChRM) directions for most analyzed samples tend toward the origin of the vector component plots, with maximum angular deviation (MAD) of 3.5° on average and a range of variation between 0.6° and 18°. All cores show quite large variations of the ChRM inclination vs depth with fluctuations around the expected GAD field value of 61° for the site latitude (41°.89N). Some noisy record is attributable to the silty nature of the sediment characterized by the presence of cm-thick, fine sand layers that could have hampered the aligning influence of the geomagnetic field on ferromagnetic particles. A few samples present shallow or negative ChRM inclinations (e.g. at 9.16 m depth in core FB47 and at 13.83 m in core FB38; see SI Text) and none of these are related to lithological variations. The ChRM inclination profile features corroborated by ^14^C dating, allow a correlation between cores FB47 and FB43 and between FB38 and FB40 (Fig 4).

In particular, Fig 4 shows that discrete overlapping is obtained between the two paleomagnetic curves of FB47 and FB43 after a small vertical translation (~0.5 m), consistent with the apparent coeval deposition of the clayey sediment. Similarly, a match, although not as close, can also be hypothesized between the curves of the magnetic inclination recorded between 12.2 and 15.2 m depth in FB38, and between 13.0 and 15.0 m depth in FB40, with a difference of 3.2 m in elevation. This observation could imply that the FB38 sequence was displaced downward by that much from its original elevation a.s.l. (see Fig 2). While it does not provide definite evidence of coeval deposition of these two clay sections, this element combined with other evidence suggests the occurrence of a possible fault displacement between FB38 and FB40.

### Chronostratigraphic setting at Forum Boarium

Lithostratigraphic analysis of the 15 boreholes reported in Fig 2 has been conducted according to the lithological/geotechnical sedimentary model for the alluvial deposits of Tiber River in Rome [29]. Our dataset has been compared and integrated with the other few published chronostratigraphic data for the sedimentary succession of the Tiber River in Rome [2,23,30–32]. The lithotypes [29] are reported in Fig 2. The details of the stratigraphic scheme are described in Figure A in S1 File.

Stratigraphic logs as well as selected ^14^C and ceramic age constraints to sediment aggradation are shown in the composite cross-section (Fig 2), which projects all of the cores along a principal transect perpendicular to the alluvial valley of the Tiber River (yellow line in Fig 2B) according to their distance from the edge of the valley (black arrows in Fig 2B).

A homogeneous package of fine-grained sediments, corresponding to lithotype C [29], constitutes the lowest, oldest portion of the alluvial deposit. These are represented by gray anossic silty clay with frequent intercalations of silty sand and organic layers, ranging from faintly carbonized vegetal remains to peat, accounting for a repetition of different low-energy environments, including open alluvial plain, palustrine and ephemeral lacustrine (elbow lake) conditions. Although there is no lateral overlapping among the portions of this type of sediment recovered at both FB48 and FB47 and that recovered at FB38, and between that occurring at FB38 and that in FB40, depositional continuity is evidenced by four ^14^C ages of 7070±95, 6856±60, 6620±85, and 6572±111 cal yr BP in the lowest section of FB38. Moreover, the paleomagnetic analysis performed on the portions of the lacustrine/floodplain sediment cored at FB38 and FB40 shows a certain degree of overlapping (Fig 4), supporting (or at least not contrasting) the hypothesis made about coeval age and their post-depositional tectonic dislocation. Also the age of 5671±32 cal yr BP obtained on pollen at the top of the >5200 yr BP succession in FB40, when the sharp decrease in the rate of global sea-level rise occurring around 6500 yr BP is considered (see Fig 3), is not in conflict with the hypothesis of a 3 m displacement between FB38 and FB40.

In particular, the sediment aggradation curve (thick red line in Fig 3) shows an increase in sedimentation rate between 6500 and 5500 yr BP, which is not in agreement with the general trend, either inferred from the predicted sea-level (gray curve in Fig 3), or from previous published data for the Tiber Valley (blue curve in Fig 3). However, when the hypothetical ~3 m of tectonic displacement between FB40 and FB38 (black arrow in Fig 3) is amended (open red symbols in Fig 3), the resulting curve of sediment aggradation (thin dashed red line in Fig 3) resumes the sub-horizontal trend consistent with the progressive decrease in sedimentation rate expected for this time span.

The occurrence of an unconformable, erosive contact at the top of the lithotype C sedimentary succession (thin dashed line in Fig 2), above which the markedly different lithotypes A1-2 and B1-2 are present, is attested by a number of factors:

1. First, the overall oxidized condition characterizing the sedimentary succession above this contact,
2. the higher energy depositional conditions, evidenced by the occurrence of several coarse grained layers, ranging from fine gravel to coarse sand;
3. the overall grading upward features of this overlying succession (lithoypes B1 and B2), with gravel at the base incorporating organic matter of the substrate (i.e. the sample dated 8082±83 cal yr BP few centimeters above the contact in FB48);
4. the young ages characterizing the sediments at the base of this upper sedimentary succession, immediately above the contact (FB47) or slightly above it (FB48), contrasting with the much older ages of the sediment occurring immediately below it (see FB48) or at a higher elevation (see FB38).

These features support the existence of an erosional phase followed by a new aggradational event starting at least 4465±55 cal yr BP (see FB48) that causes the initial deposition of gravel within the fluvial channel, followed by deposition of clay in the alluvial plain. The ^14^C age of 2800±50 cal yr BP around 1 m b.s.l. in FB48 indicates that the early stages of this new aggradational phase are characterized by a slow sedimentation rate (A light blue arrow in Fig 2). Instead, sudden flooding of the alluvial valley (B light blue arrow in Fig 2) is observed since around 2600 yr BP, leading to the deposition of a several-meter-thick package of sediments in approximately one century.

As the three ^14^C dates from these deposits in borehole FB39 fall within the range of the Hallstatt Plateau, ceramics indicate a more precise date around 2550 BP (combining data from FB39, 43, 47, 48) for the beginning of the fast aggradation phase. In addition, building phases at the Sant’Omobono temple, whose tuff platform encountered in borehole FB 47 rests above the alluvial succession, provide key chronological upper boundary at 2430 yr BP for the end. Therefore, the archaeological evidence suggests a time span comprised between the beginning of 6^th^ and early 5^th^ century BCE for the deposition of the sediments comprised between 1 m b.s.l. and 5 m a.s.l. at cores 39, 43, 47, in the Forum Boarium, corresponding to lithotypes A1 (basal, fluvial channel facies) and A2 (upper, floodplain facies).

The coeval, homogeneous filling of this area of the alluvial valley is consistent with the overlapping paleomagnetic signals recorded in the two portions of sediment occurring at the same elevation in core FB43 and FB47 (Fig 4). This sediment is composed of yellow silty clay with frequent, cm-thick, fine sand layers, consistent with for low-energy conditions.

Rapid re-incision of this massive package of sediments soon after its deposition is supported by the occurrence of ceramics dated 2400–2300 yr BP at elevation around 4 m a.s.l. (FB38 and FB46). In contrast, the anthropic fill at ca. 1 m b.s.l. incorporating ceramics dated ≤2100 yr BP at FB49 is problematic, and cannot be explained with the re-incision of the riverbed down to a depth below the sea-level. However, sediment compaction and/or human intervention can justify the observed low elevation of the base of anthropic fill.

Finally, a new sudden sediment aggradation is evidenced around 2250 yr BP, based on the rising of a thick anthropic fill up to the previous elevation of the floodplain at around 9 m a.s.l. (FB38, FB49), and based on the contemporaneous deposition of floodplain sediments (C blue arrow in Fig 2) in this time span (FB44, 45, 46, 47).

## Discussion

### Geochronological and archaeological constraints to sediment aggradation in the Tiber valley

The aggradational history in response to the post-glacial sea-level rise along a N-S cross-section longitudinal to the Tiber River Valley, reconstructed using ^14^C dates from previous work [2,22,24] and from the present study, is shown in Figure C in S1 File and described below.

The study of the sedimentary sequence of the Tiber River dating 15.000–6.000 yr BP has been thoroughly presented and discussed in the cited previous work, which showed that an overall synchronous aggradation of clastic sediments characterized the >20 km terminal tract of the fluvial channel and the coastal plain (Figure C-*A* in S1 File) since the end of the Last Glacial Cycle, as evidenced by a sharp lithological transition between gravel and clay that formed between 13.6±0.2 and 12.8±0.2 cal BP ka, and by parallel, sub-horizontal isochron lines across the upper package of clayey sediments. Geochronologic constraints provided in Marra et al. [1], show that most of the gravel aggradation occurred since 15 ka through 13 ka, indicating that the timing of the sedimentary switch to fine sediments overlaps meltwater pulse (mwp) 1a, as statistically identified in Stanford et al. [33], closely following the maximum rate of sea-level rise during the Last Glacial Termination. This observation is consistent with the fact that transportation of very coarse gravel (> 5 cm diameter) by the Tiber River requires exceptional hydrologic conditions that were seen during glacial terminations only and have not been repeated during the Holocene. Such conditions existed due to a combination of: (i) increased sediment supply to the Tiber drainage basin due to rapid melting of Apennine glaciers that released large amounts of clastic material; and (ii) low sea levels that caused a steep topographic gradient, hence greater and more energetic river transport capacity. Eventually, accelerated sea-level rise during terminations caused a rapid drop in transport capacity of the Tiber River, which in turn resulted in sandy clay deposition in a less energetic environment. Finally, almost complete infilling occurred of the fluvial incision that was excavated during the lowstand. Thus, the floodplain approached present-day sea level, which for the last glacial termination occurred at around 6000 yr BP (Figure C-*A* in S1 File).

The steady aggradation of the Tiber River alluvial succession during the time span 13.000–6000 yr BP is remarkably similar to that reconstructed for the Arno River [34], while it differs from that outlined in the Po River delta for the same time span, where a bi-partition of this aggradational phase is marked by a paleosoil developed during the Younger Dryes event, 12.000–10.000 yr BP [35].

After this almost complete resumption of the sea-level, the occurrence of an intervening erosional phase (Figure C-*B* in S1 File), previously dated around 4500 yr BP [2], is supported and constrained more closely by the new data from Maxxi and Forum Boarium boreholes. This event, re-incising the floodplain formed 5500 yr BP and accounting for a base-level as low as 7 m b.s.l. for the Tiber River in Forum Boarium is now dated around 5200 yr BP.

A sea-level so low clearly is not compatible with literature data on global sea-level at 5200 yr BP; however, this datum needs to be corrected for sediment compaction in the alluvial body. Marra et al. [2] have evaluated sediment compaction through a geotechnical model for the different stratigraphic columns in which the dated samples occurred, assessing that the variation in elevation may be as large as several meters. In Figure C-*B* in S1 File the corrected elevation and the associated error for each of the dated samples is reported (thick circles and vertical bars): when the original elevation for the different samples is restored, the depth of the re-incision (thinner red dashed line in Figure C-*B* in S1 File) reaches a maximum depth of ~4 m b.s.l., which is not much lower than the estimated global sea-level for the Tyrrhenian Sea at 5000 yr BP [25]. Whereas discussing the evidence for a moderate sea-level drop in this time span (5500–5000 yr BP) is beyond the scope of the present study, the occurrence of this erosional phase cannot be questioned and its causes may be explained without necessarily invoking a sea-level change [2]. Re-incision of the alluvial plain in the mid-late Holocene is a phenomenon observed also in the Arno River coastal plain [36–38]. A marked phase of incision, broadly constrained during the pre-Roman period (6000–2500 cal yr BP), has been documented at the archaeological site of Pisa San Rossore, where Benvenuti *et al*. [36] suggested a possible control on channel erosion by small-scale sea-level lowering, as Marra *et al*. [2] did for the similar event occurring in the Tiber River hydrographic network. In contrast, Sarti *et al*. [38] have distinguished two erosive events in the coastal plain of the Arno River, chronologically constrained to the Eneolithic-Bronze age transition (~ 3800 cal yr BP) and to the Bronze–Iron age transition (2900–2800 cal yr BP), which the authors interpret as reflecting centennial-scale changes in the aggradation/degradation ratio, correlated with two phases of increased humidity (Abies peaks 1 and 2) recorded in several sites of Europe [38].

More generally, a widespread period of climatic change has been documented in the Central Mediterranean Region (4.2 ka event, see [39] for a review), with two phases characterized by wetter conditions at ca. 4300–4100 and 3950–3850 yr BP, bracketing a dry event at ca. 4100–3950 ky BP. We note that the mid-Holocene erosive phase in the Tiber channel broadly encompasses all the paleo-climatic events since 4.2 ky BP mentioned above, but largely predates their initiation, suggesting that a severe climatic (and/or eustatic?) fluctuation already occurred around 5.2 ky BP.

Indeed, the new chronological data from Forum Boarium allow a better constraining of the beginning of this phase and show that re-filling of the previous incisions was achieved around ~2800 yr BP (Figure C-*C* in S1 File), consistent with termination of the latest erosive phase during the Bronze-Iron age transition documented in the Arno River [38]. Moreover, these data indicate the anomalous occurrence of an important sudden and fast accumulation of sediments within this portion of the Tiber Valley since ~2600 yr BP, clearly independent from the sea-level trend, which in this time span recovers very modestly from 2.5 to 1.5 m b.s.l [25] (Figure D in S1 File).

### Reconstruction of the sedimentary and tectonic history at Forum Boarium

#### 6.000–2.800 yr BP interval: Mid-Holocene erosive phase

In close agreement with the general aggradation history of the Tiber Valley described above, ^14^C age at FB40 shows that at 5671±32 cal yr BP the alluvial plain of the Tiber River in Forum Boarium reaches a maximum flooding surface around 4 m a.s.l. (borehole FB 38, Fig 2 and Fig D-*A* in S1 File). There follows an erosional phase that re-excavates the gray clay, low energy, deposits representing this flood plain (boreholes FB 43-47-48, Fig 2 and Figure D-*B* in S1 File). Above the erosional surface, dated at around 5200 yr BP by the Maxxi core, oxidized, yellow sandy clay deposits of higher energy environment indicate the starting of the new aggradational phase. Age constraints from boreholes FB48 and BF47 suggest a slow, progressively decreasing sedimentation rate characterizing the time interval ~5200 - ~2800 yr BP, for a total thickness of 6 m of sediment in ca. 2400 yr (A arrow in Fig 2. Figure D-*C* in S1 File). This is consistent with the trend of the global sea-level curve accounting for a slow recovery from 4 m to 2 m b.s.l. in this time span (see also Figure C-*C* in S1 File).

The isochrone lines in Fig 2 give insights about the landscape of the Forum Boarium area around 2800 yr BP, at the beginning of the 8^th^ century BCE. The area is characterized by a small terraced section of floodplain beneath the Sant'Omobono temple, at the foot of the Capitoline slope, facing the Tiber River Valley at the confluence of the tributary stream valley of Velabrum Maius (see also Fig 2B). These relatively stable paleogeographic conditions likely endured until the late 7^th^ century BCE.

#### 2600–2400 yr BP: Sudden rise of the alluvial plain

In contrast, sediment aggradation, dated between 2550 yr BP and 2430 yr BP by ^14^C and archaeological ages, indicates a marked and dramatic change of the landscape since the early 6^th^ century BCE, with the rapid inflation of the alluvial plain caused by the sudden deposition of a 4 to 6 meter-thick package of sediments in approximately one century (Fig 2).

It is not simple to explain such a large increase in sedimentation rate, or the rise of the floodplain of the Tiber River from ca. 1 m up to 5–8 m a.s.l., especially when considering the substantially stable sea-level characterizing this time span. Such a dramatic change in sediment supply would require a substantial hydrologic change, with a significant increase in rainfall causing repeated, outstanding flooding events and/or a massive anthropic impact on the landscape triggering erosion.

#### Climate variability and hydrologic regime during the Holocene in central Italy

While it has proven difficult to disentangle natural forces from human induced modifications during the mid and late Holocene especially for processes such as river hydrology and sedimentation [40], temporal clusters of synchronized flood activity have been identified in the Mediterranean and have been related to atmospheric triggers [41]. Different rapid climate change (RCC) episodes and centennial scale phases of increased humidity have been recently recognized in the Central Mediterranean for this period [42]. More specifically, a pronounced climate instability and frequent paleo-hydrological oscillations can be observed in peninsular Italy starting around 4200 yr BP [43] with more frequent and intense alluvial phases until 2500 years BP [44].

In Central Italy, several lakes record climatic shifts, seasonality trends, rainfall patterns, as well as forest clearance during the Holocene [45–51]. A low rainfall phase, with higher summer temperature, cooler winters and low seasonal precipitation variation, corresponds to the Bronze Age [52,53], and it is followed by a period with a marked increase in precipitation 2700–2300 yr BP [42] that could be related to the unusual flooding event in the Forum Boarium.

Using data from North-East and peninsular Italy, Benito et al. [54] suggest a flood cluster with overlapping periods at 2300–2100 yr BP and 2350–1850 yr BP that, however, are not consistent with the sedimentary record of the Forum Boarium.

In addition, palynological data support anthropic impact on the environment, as well as episodes of increased erosion in the lakes catchment during the late Holocene. Multiple cycles of forest reduction and re-growth coincide with the expansion of human settlements in central Italy, as seen at Lago di Mezzano and at Lago di Vico at 2900–2800 yr BP [52], at Lago di Albano and Lago di Nemi at 3800–3600 and 3000–2800 yr BP [55]. A parallel marked increase in cultivated trees after 2800 yr BP suggests large-scale human induced vegetation change. This is particularly apparent at Valle di Castiglione, where there is also an increase in anthropogenic indicators 3000–2600 uncal. yr BP [56].

In the Tiber valley, sediments retrieved in the harbor of ancient Ostia at the river mouth [57] are in general good agreement with the Forum Boarium sequence, but they lack the chronological discrimination needed for a more precise correlation. They show a low energy environment starting around 2850–2750 BP, while, right under the date 2257±96 cal yr BP (403–211 BCE), a high energy level is characterized by enhanced erosion in the catchment and increased amount of pollen transported by the river. Flooding evidence is also found in the Fiume Morto (a paleo-meander of the Tiber delta) area beneath the date 2184±122 cal yr BP [58,59].

In light of these considerations, however, we suggest that a shift in the river hydrology triggered by modified precipitation patterns and a change in the vegetation cover cannot by themselves account for the sudden and unprecedented accumulation of alluvial deposits in the 6^th^ century BCE. Indeed, only a few of the previous several RCC episodes of increased humidity and rainfall that occurred during the mid-late Holocene [42] can be recognized in the Tiber River sedimentary record at Forum Boarium, and they do not seem to have affected the river with comparable magnitude. Even if we hypothesize a correlation between the transition to a wetter climatic phase 4500–4000 yr BP and the observed re-infilling of the Tiber valley after 5200 yr BP, we do not see a sedimentation influx as large as that recorded during a much shorter interval in 2600–2400 BP. On the other hand, sudden flood episodes registered in the river delta in the 2700–2200 BP interval are just decimeter-thick [57] while upriver, in the Rieti basin, the maximum highstand of the lake is observed in the 9^th^ century BCE with sediment accumulation remaining low until the 3^rd^ century BCE [60].

In addition, variation in woodland density, with several episodes of reduced forest cover, is well documented for the whole mid-late Holocene period. In particular, clear anthropic deforestation seems to start few centuries earlier (around 1000–800 BCE) without such a visible immediate effect on the Tiber sedimentation rate (see Fig 3).

#### 2600–2400 yr BP: Tectonic displacement of the 6^th^ century alluvial plain?

One possible alternative explanation to this remarkable alluvial aggradation is the co-occurrence of tectonic subsidence within the Tiber Valley, accounting for a sudden increase in accommodation space, which allows for the observed rapid sedimentary filling.

**Fig 5:**
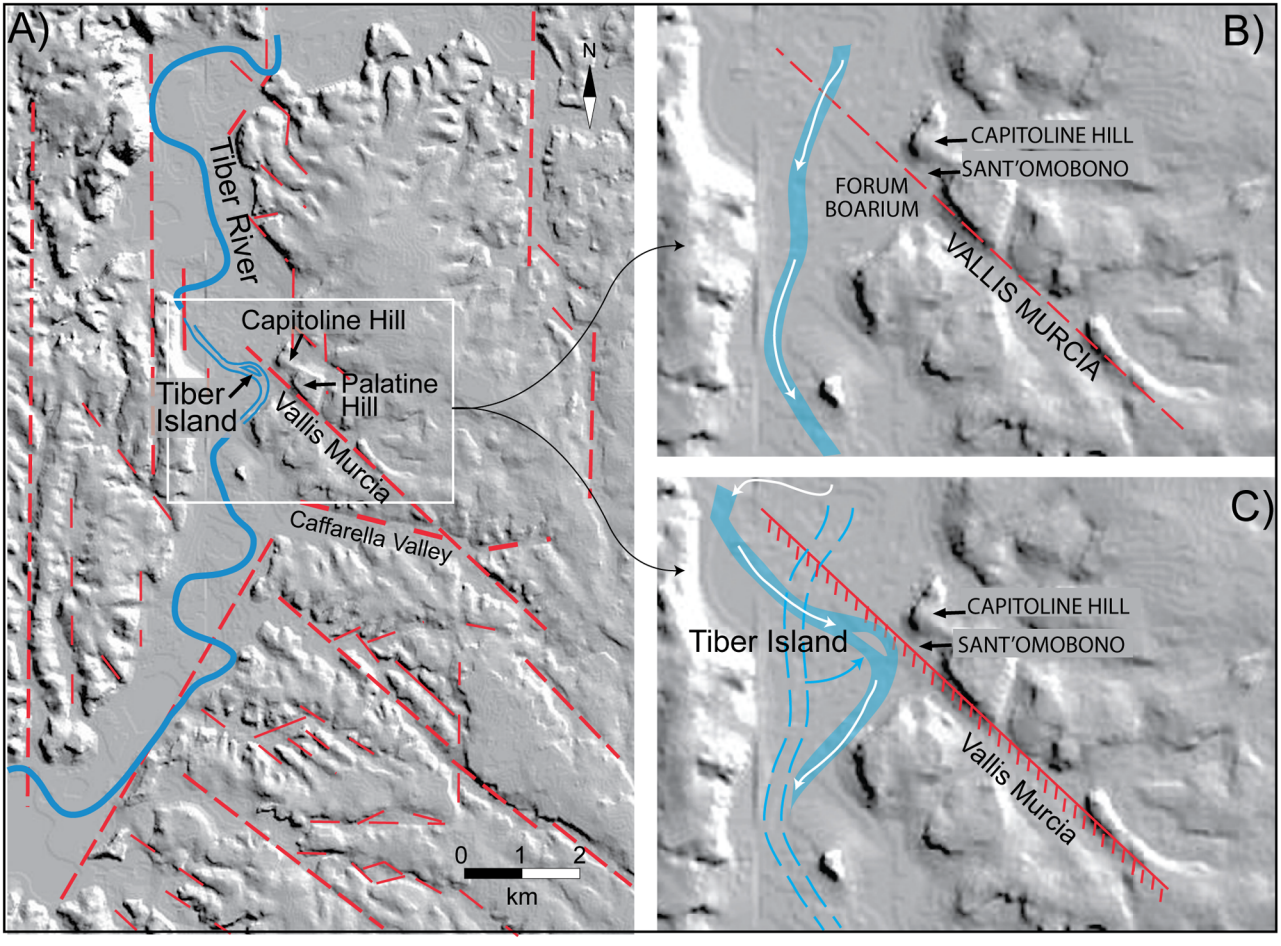
(*A*) digital elevation model (DEM) image of the area of Rome (TINITAL/01 square WA 6570; under permission by Istituto Nazionale di Geofisica e Vulcanologia, Italy) in which a set of inferred fault segments controlling the principal NW-SE and N-S striking streambeds and alluvial valleys [15] is reported (red dashed lines). (*B*) Hypothesized landscape before the fault activity. (*C*) Tectonic displacement, through the lowering and the NE tilting of the fault's hanging wall, diverts backward the river course toward the fault scarp, causing a loss in capacity of transport as well as the delivering of the sediment that formed the Tiber Island.

In a digital elevation model (DEM) image of the area of Rome (Fig 5) is possible to infer a set of fault segments (red dashed lines) controlling the principal NW-SE and N-S striking streambeds and alluvial valleys [15]. Remarkably, one of the most evident and continuous NW-SE morpho-structural lineament is the one coinciding with the Vallis Murcia, a tributary valley, which joins the Tiber Valley in the Forum Boarium area.

Such feature suggests that the Tiber Island, located exactly along the continuation of the inferred fault segment, may be the result of its continued activity in early historical times. Indeed, the Tiber course seems strongly controlled by the presence of the fault in this sector, which through the lowering and the NE tilting of its hanging wall, may have generated a step acting as a natural dam to the riverbed (Fig 5B and 5C). Tectonic displacement may have diverted backward the river course toward the fault scarp, relenting its flooding velocity and causing a loss in capacity of transport as well as the delivering of the sediment that formed the Tiber Island, before continuing its southward flowing.

Tectonic subsidence seems also suggested by the possible coeval age and postulated 3.2 m dislocation of the two lacustrine sections at different elevation in FB38 and FB40, as inferred by paleomagnetic analysis.

#### Landscape transformation and birth of the Tiber Island

**Fig 6:**
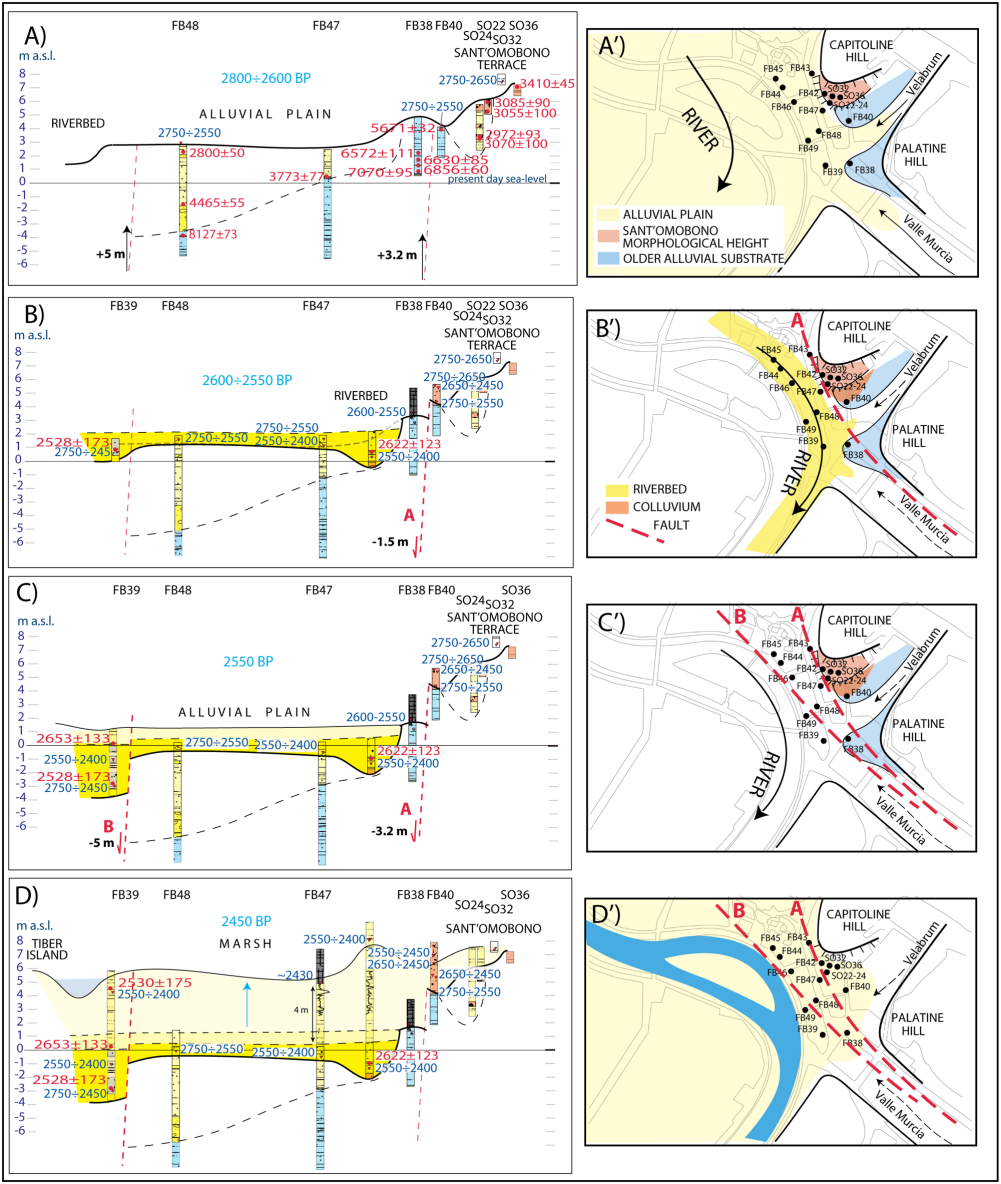
Step-by-step reconstruction of the evolution of the landscape in Forum Boarium at 2880–2450 yr BP, through the combined effect of sediment aggradation and fault activity. Archaeological and 14C ages as in inset of Fig 2.

Based on the geomorphological evidence described above, we have restored the original stratigraphic setting in the investigated area by hypothesizing a fault system characterized by a main NW-SE striking lineament developing in two parallel, en-echelon segments. Surface trace of these fault segments (A, B) is shown in the maps of Fig 6, and their position is reported in the cross-sections of this figure. We have estimated vertical slips of 3.2 and 2 m for these fault segments, in order to account for the present displacements offsetting the sedimentary deposits, and we have restored cross-section of Fig 6A by uplifting each faulted block for the corresponding elevation gain (black arrows in Fig 6A). Starting from this undeformed original landscape at around 2800 yr BP, we have reconstructed a step-by-step evolution of the area, to reproduce the present stratigraphic setting and to explain the rapid sedimentary filling of the alluvial plain in this sector between 2600 and 2400 yr BP.

Fig 6A shows the landscape in the 8^th^-7^th^ century BCE, when a raised fluvial terrace at the foot of the Capitoline Hill overlooked the alluvial plain of the Tiber River. The shifting of the riverbed close to this sector is recorded around 2600 yr BP by the presence of a continuous layer of coarse sediments in FB39, FB48, FB47 and FB43. This is interpreted in Fig 6B as the effect of incipient faulting, causing the shifting of the riverbed towards the eastern edge of the alluvial valley, consistent with the initial slip of the easternmost fault segment (A), as shown in the map view of Fig 6B'.

The continued activity of this fault and of the second segment (B) during the 6^th^ century BCE provides differential collapse of the western sector of the alluvial plain, for a cumulated displacement of 3.2 and 5 m, respectively (Fig 6C). This causes the further shift of the riverbed, whose position is controlled by the largest cumulated displacement released along the western segment B (Fig 6C'). Evidence for this shift is supported in FB39 by the thick coarse sedimentary layer accumulated in a relatively short time interval and bracketed by ^14^C ages of 2528±173 and 2653±133 cal yr BP, as well as by consistent ceramic age of 2600–2400 yr BP, (Fig 6C).

The faulting process and the consequent lowering of the adjacent sector of the alluvial valley probably needs to be understood as a slow, progressive phenomenon, during which the newly created accommodation space is continuously filled by sedimentary material released by the river. The riverbed is constrained by the fault scarp along the B segment, acting as a dam and providing the condition for the origin and the stable positioning of the Tiber Island (Fig 6D'). The progressive subsidence caused the rapid accumulation of sediments (Fig 6D) due to the loss in capacity of transport of the river entering the collapsing basin. Between the mid-6^th^ and the mid-5^th^ century the area in front of Sant'Omobono became a subsiding marshy plain, crossed by the river whose ephemeral bed was controlled by the fault scarp.

^14^C and archaeological age constraints on the ~4 to 7 m thick sedimentary fill (from the center of the valley, i.e.: FB47, towards its edge, i.e.: FB43) indicate that the action of faulting and the related subsidence ended in the second half of the 5^th^ century BCE (~2430 yr BP), when a stable flooding surface was established between 5–6 m a.s.l. (Fig 6D). However, the elevation of this floodplain is 2–3 m higher than that of the alluvial plain around 2600 yr BP (Fig 6A), when the collapsing of this area started. Multiple concurring causes are needed in order to justify the deposition of a package of sediment 2–3 m thicker than the accommodation space (ca. 3 m) created by tectonic subsidence. Part of this rise in elevation is attributable to the progressive filling of the entire alluvial valley of the Tiber River that had been ongoing since 5200 yr BP, while the RCC at 2700–2300 yr BP, as well as the progressive woodland cover reduction since 2800 yr BP also clearly played a role. In addition, we suggest that anthropic impact on the landscape contributed to the increase in sediment transport that occurred since the early 6^th^ century BCE.

It is important to emphasize here that this sedimentary event coincides with the rapid growth of the settlement in Rome and the emergence of the first cities in the region, as reflected in the archaeological record. Debate on precise chronology as well as on the nature of the social and economic processes involved is still ongoing [61] and different contrasting models have been proposed for estimate of population growth and agricultural intensification [62]. Still, most scholars agree that by the late 6^th^/early 5^th^ century BCE settlement patterns in Central Italy are characterized by bigger urban settlements and a marked increase in the number of rural sites [63]. In Rome, in particular, from the late 7^th^ to the early 5^th^ century BCE, a plethora of new building projects were undertaken, marking the transformation from hut settlement to city [64]. These constructions employ stone architecture and engage in landscape modification on a scale not previously seen at Rome, suggesting that such a surge in urban development would have required considerable resources. We argue that this urban growth and building activity, well documented in the archaeological record of archaic Rome, would have occurred simultaneously with an increase in logging activities and a change in land management that would have had a profound impact on the local and regional landscape increasing soil erosion and sediment accumulation.

A further explanation that could justify the observed sedimentation pattern is that the faulting in the Forum Boarium sector was part of a larger tectonic process affecting the area of Rome, characterized by a general subsidence in the order of 2–3 m, and by localized, deeper collapsing of fault-controlled small sectors.

#### 2400–2250 yr BP: Third aggradational phase and the river harbor

**Fig 7:**
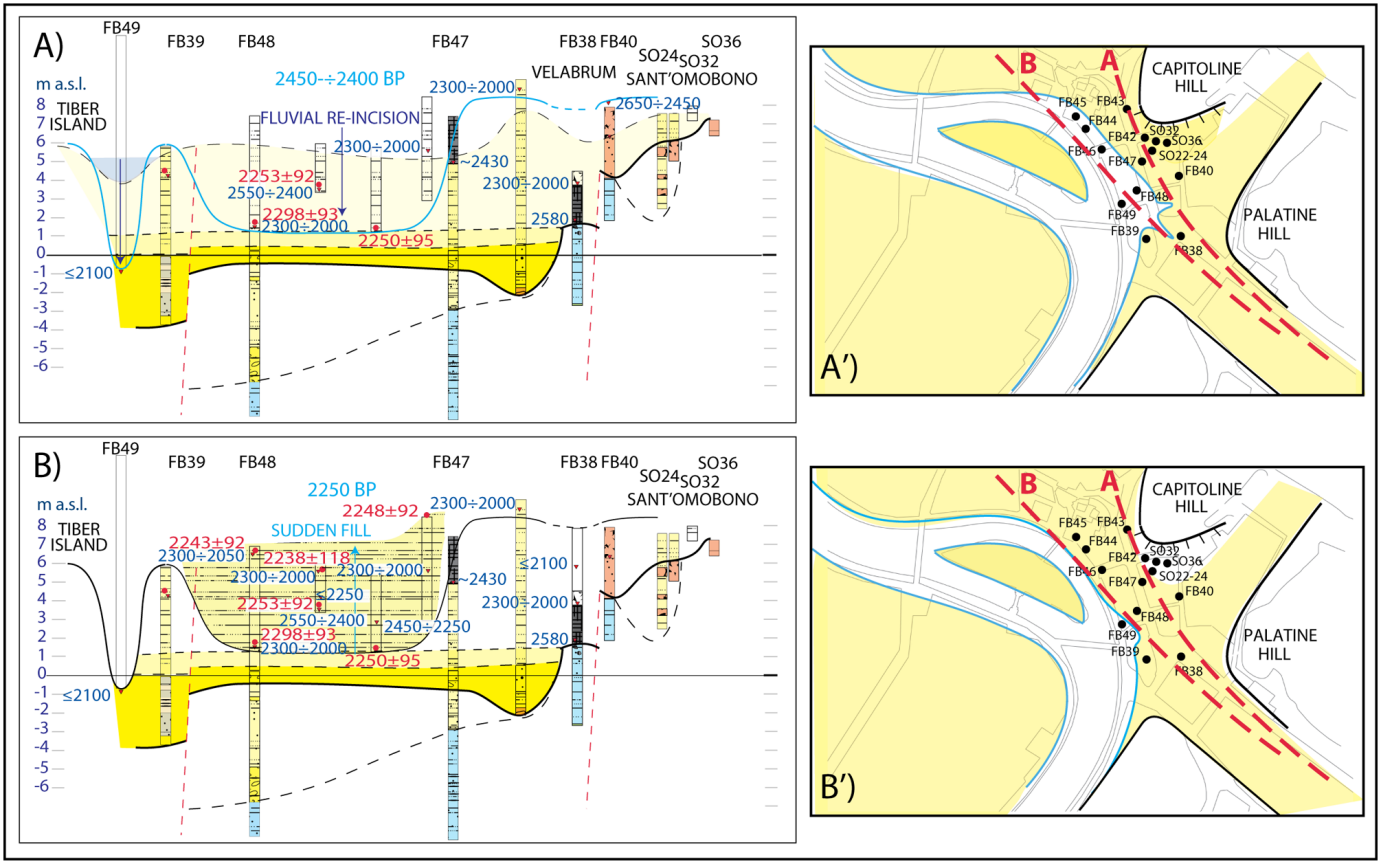
Step-by-step reconstruction of the evolution of the landscape in Forum Boarium at 2450–2250 yr BP. Archaeological and 14C ages as in inset of Fig 2.

A rapid, marked re-incision of the ‘overhanging’ alluvial plain is evidenced by stratigraphy at boreholes FB38, 44, 45, 46, 48 (Fig 7A), where a new sudden fill of this paleo-incision seems to occur in the 3^rd^ century BCE, as indicated by the homogeneous age around 2250 yr BP of ^14^C dated samples and ceramic fragments occurring within the sedimentary deposits recovered in FB44, 45, 46, 48 (Fig 7B).

This re-incision of the alluvial plain may be partially explained by the fact that, after the end of the subsidence, the alluvial plain was re-excavated by the erosive action of the riverbed (Fig 7A'), to re-establish its hydrographic profile at the same elevation (~1 m a.s.l.) as that it had at the beginning of the 6^th^ century BCE. However, the occurrence of ceramic homogeneously dating to the mid-3^rd^ century BCE throughout the entire thickness of the sedimentary package, as documented by the stratigraphy of cores FB44, 45, 46 and 48, indicates concurrent anthropic activities.

Indeed, the re-incision of the alluvial plain may be explained through dredging of the fluvial channel since the mid-5^th^ century BCE, aimed at defending the river harbor in the Forum Boarium. Although there is a substantial historical record of dredging of the Tiber channel [65], this investigation of the alluvial sequence in the Forum Boarium valley provides the first empirical evidence of this practice in the presumed location of Rome’s original river harbor [66]. Dredging would have been made necessary by the progressive silting up of the basin. Continuous flooding episodes between the 4^th^ and the 1^st^ century BCE are supported by the sediment stratigraphy at the river delta [59] Indeed, the literary sources from ancient Rome report the occurrence of floods in the city (the oldest flood mentioned by Livy is reported for the 5^th^ century (414/419) BCE; [67]). The documentary record describing the frequency and magnitude of the events improves significantly during the period between 200 BCE and 200 CE. As a result, several scholars, extrapolating from the historical sources, argue that major flooding events occurred in this period as a consequence of intensive deforestation [65,68]. Therefore, the successive new sudden fill in the mid-3^rd^ century may indicate abandoning of the river port and ceasing of dredging activity.

## Conclusion and final remarks

The aggradational history of the Tiber River in central Rome is defined by three significant erosional/depositional events, which appear unrelated to the global eustatic signal and only partially related with the climatic events for the Holocene.

The earliest event is characterized by a marked erosional phase around 5200 yr BP, re-excavating down to -6 m a.s.l. an early alluvial plain of the Tiber River established in Rome since ~5600 yr BP at elevation around 4 m a.s.l. Lack of information on the full thickness of the investigated sedimentary succession at Forum Boarium hinders assessment of variation in original absolute elevation of the dated erosional surface due to time-dependent sediment compaction, preventing us from discussing possible eustatic/tectonic versus climatic/sedimentological forcing of this erosive phase.

The second event follows the progressive recovery of the floodplain up to present day sea-level, and is characterized by the sudden deposition of an ca. 6 m-thick package of sediment in one century, 2550 through 2450 yr BP. Based on the analysis of convergent geomorphological, structural and chronostratigraphic elements, corroborated by paleomagnetic investigation, we test the hypothesis that the abrupt overflooding of the river valley may be the consequence of fault displacement along a morpho-structural lineament crossing the Forum Boarium area, creating coupled increase in accommodation space and loss in capacity of transport by the river. Far from being definitive, the provided evidence accounts for the hypothesized fault displacement causing the consequent deposition of an extraordinary thick succession of sediments, the diversion of the river course, and the birth of the Tiber Island between the 6^th^ and the early 5^th^ century BCE. However, concurrent climatic and especially anthropogenic causes are likely to contribute to the increased sediment accumulation. In this scenario, the deposition of several meters of alluvium would be indeed the result of several factors. Sediment released by increasing erosion, due to growing anthropic disturbance and landscape modifications connected to urbanization processes occurring in the 6^th^ century BCE, further exacerbated by a shift towards wetter climate and few centuries of deforestation, would find a trap in the space created by the fault.

Archaeological evidence demonstrates a significant increase in anthropic activity on the local landscape beginning in the late 7^th^ century BCE. Urban growth in 6^th^ century Rome is characterized by large-scale building and landscape modification projects, efforts that are well-documented and coincide with the marked increase in sedimentation in the Forum Boarium valley.

As the city of Rome continued to expand in Republican Period, a third, less marked rapid increase in elevation of the alluvial plain occurs during the 3^rd^ century BCE.

By providing new data on the changing hydrology of the Tiber River over the entire Holocene, this reconstruction has both scientific and historical implications for our understanding of the origins of the Eternal City and the impact of urban systems on the local and regional landscape. Additional cores, both on the Tiber Island and north of the fault displacement, are needed in order to fully support these hypotheses as well as framing them within the historical and archaeological context of the early occupation of Rome.

## Supporting information

S1 File Supplementary text and Figures. (Word) **Figure A—Lithological/geotechnical sedimentary model for the alluvial deposits of Tiber River in Rome**. See text for legend and description. **Figure B—Measured magnetic parameters.** Downcore variation of natural remanent magnetization at 0 mT (NRM), characteristic remanent magnetization (ChRM) declination and inclination, maximum angular deviation (MAD), magnetic susceptibility (k), anhysteretic remanent magnetization (ARM), median destructive field of the NRM (MDFnrm). See Methods section in main text for further explanations. **Figure C—Aggradational history of the Tiber River.** ^14^C age constraints provided by literature data (Marra et al., 2013; Belluomini et al., 1986; Bellotti et al., 2007) and by the present study to sediment aggradation in the Tiber Valley between Rome and the coastline. A cross-section longitudinal to the river course is reconstructed using all available borehole data (location in Fig 1). Each panel show the stratigraphic setting at different ages; insets show the aggradation curve of the Tiber sediments (thick colored line) compared to the global sea-level curve from coral reefs data (Peltier and Fairbanks, 2006). Different aggradational and erosional phases are numbered 1 to 4. **Figure D**—**Landscape evolution in Forum Boarium.** Cross-sections showing the evolution of the landscape in the Forum Boarium area since 6000 yr BP (A), and following the 5200 yr BP erosional phase (B), until the formation of a new alluvial plain around 2800 yr BP (C), based on the reported core chronostratigraphy.
(DOC)

S2 File ^14^C and ceramic ages. All 14C ages are newly reported, except the last four (labelled SO) which have been published previously [3–5].
(DOC)

S3 File Supplementary dataset - ^14^C analyses. (PDF)

S4 File Permission to use the DEM. (DOC)
