# Supplementary material for: Rome in its setting. Post-glacial aggradation history of the Tiber River alluvial deposits and tectonic origin of the Tiber Island
Source: PLoS One. 2018 Mar 28;13(3):e0194838. doi: 10.1371/journal.pone.0194838 (PMC5874049; doi:10.1371/journal.pone.0194838)
Supplement: S1 File — (Word) Figure A—Lithological/geotechnical sedimentary model for the alluvial deposits of Tiber River in Rome. See text for legend and description. Figure B—Measured magnetic parameters. Downcore variation of natural remanent magnetization at 0 mT (NRM), characteristic remanent magnetization (ChRM) declination and inclination, maximum angular deviation (MAD), magnetic susceptibility (k), anhysteretic remanent magnetization (ARM), median destructive field of the NRM (MDFnrm). See Methods section in main text for further explanations. Figure C—Aggradational history of the Tiber River. 14C age constraints provided by literature data (Marra et al., 2013; Belluomini et al., 1986; Bellotti et al., 2007) and by the present study to sediment aggradation in the Tiber Valley between Rome and the coastline. A cross-section longitudinal to the river course is reconstructed using all available borehole data (location in Fig 1). Each panel show the stratigraphic setting at different ages; insets show the aggradation curve of the Tiber sediments (thick colored line) compared to the global sea-level curve from coral reefs data (Peltier and Fairbanks, 2006). Different aggradational and erosional phases are numbered 1 to 4. Figure D—Landscape evolution in Forum Boarium. Cross-sections showing the evolution of the landscape in the Forum Boarium area since 6000 yr BP (A), and following the 5200 yr BP erosional phase (B), until the formation of a new alluvial plain around 2800 yr BP (C), based on the reported core chronostratigraphy. (DOC) [file pone.0194838.s001.doc]

**Supporting Information**

**Rome in its setting. Post-glacial aggradation history of the Tiber River alluvial deposits and tectonic origin of the Tiber Island.**

**Authors:** Fabrizio Marra^1*^, Laura Motta^2^, Andrea L. Brock^3^, Patrizia Macrì^1^, Fabio Florindo^1^, Laura Sadori^4^, Nicola Terrenato^3^

**Affiliations:**

^1^Istituto Nazionale di Geofisica e Vulcanologia, Via di Vigna Murata 605, 00143 Rome, Italy; ^2^Kelsey Museum of Archaeology, 434 S. State St, University of Michigan, Ann Arbor, MI 48109-1390;

^3^Department of Classical Studies, 435 S. State St, University of Michigan, Ann Arbor, MI 48109-1003;

^4^Dipartimento di Biologia Ambientale, Universita di Roma “La Sapienza”, Piazzale Aldo Moro 5, 00185 Roma, Italy.

*corresponding author: fabrizio.marra@ingv.it

**Sedimentary model for the Tiber River**

The lithostratigraphic analysis has been conducted according to the lithological/geotechnical sedimentary model for the alluvial deposits of Tiber River in Rome provided in Bozzano et al. (2009), who identified several lithotechnical classes of sediment, based on their physical and mineralogical features, as previously also described in Corazza et al. (1999) and Bozzano et al. (2000).


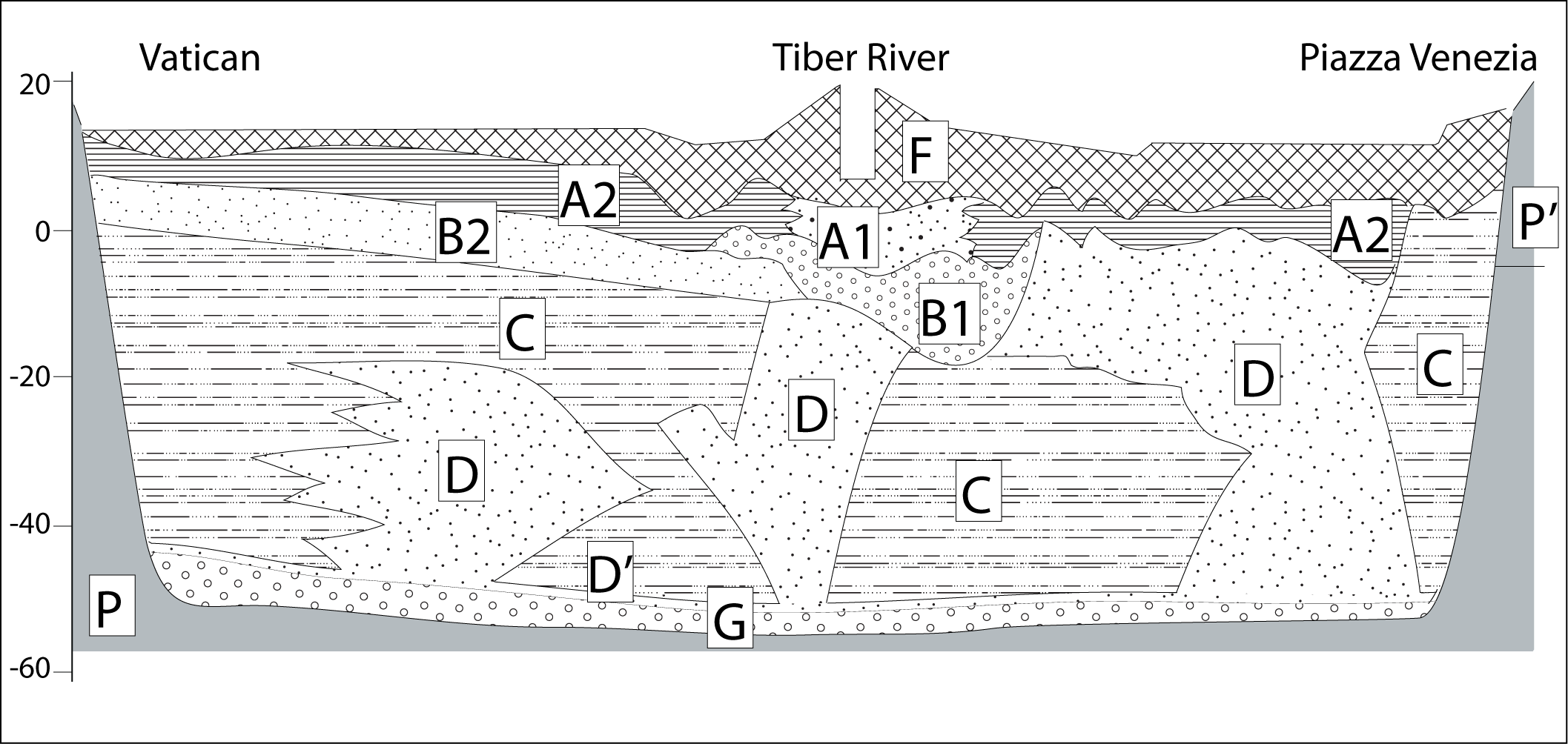


Figure A. Lithological/geotechnical sedimentary model for the alluvial deposits of Tiber River in Rome. See text for legend and description.

This model is summarized in the stratigraphic scheme illustrated in Fig. S1, which is based on a large number of borehole data along a transect of the Tiber Valley between the Vatican and Piazza Venezia. A basal layer of coarse gravel (level G), 8–10 m thick on average, is continuously present with erosive contact above the Pliocene bedrock (P). An approximately 2m-thick sand layer (level D') occurs above this contact throughout the valley and is followed by a several meter thick silty-clay layer (level C), which is laterally in contact with a clayey sand deposit (level D). The silty-clay deposits of level C, representing alternating low-energy palustrine and lacustrine sedimentary facies, and slightly more energetic alluvial plain facies, are dominant at the margins of the valley. In particular, on the eastern bank of the valley, near Piazza Venezia, the silty clay sedimentary package of level C displays more vertical continuity, reaching a maximum thickness of about 50 m and top elevation close to 8 m a.s.l.. In contrast, the sandy deposits of level D represent the fluvial channel facies, which concentrates in the middle of the valley. Above lithotypes D and C, the alluvial deposits sharply reverse their grain size: level B1 (medium-coarse sand) representing the riverbed facies, and level B2 (silty sand and clay) representing the alluvial plain deposits, have respectively lenticular or tabular geometries. More fine-grained deposits constituting the "historical" alluvia of the Tiber (level A) close the sedimentary succession. Two facies can be distinguished: level A1 (silty sand), and level A2 (clay silt). A thick cover of anthropic fill (level F), ranging from the 3^rd^ century BCE (Republican period) to the present-day, interfingers and eventually overlies the historical alluvia.

**Chronology**

**Radiocarbon dating**

A total of 117 sediment samples were screened with a 250-micron mesh for plant macro-remains, animal bones, ceramic fragments as well as building material. Organic remains were selected for AMS radiocarbon dating according to the following criteria. Seeds were the preferred material, and if a single seed was too small, multiple seeds from the same samples were combined. When seeds were not available, either waterlogged or charred wood from non-aquatic taxa and above-ground plant structures was used. We did not recover any macro-remains from core FB48 below -1m a.sl. therefore bulk samples were processed for the extraction of plant matter. The same scarcity of macro-remains was observed between 2 m a.s.l. and-1 a.s.l. in core FB38, however, we were able to extract enough pollen for AMS dating from the lacustrine sediments.

All radiocarbon dates and their calibrated equivalents together with the elevation of the dated samples and core information are presented in S2 Table.

**Ceramic material**

The diversity and abundance of the ceramic record included in the sequence of the anthropic deposits provides a nuanced and reliable chronology. We relied on fine wares such as Etruscan bucchero, as well as Attic and Corinthian ceramic, which can be precisely dated by fabric, shape, and/or decoration, to provide detailed age constraints to the stratigraphy for the period between 700-400 BCE. In addition, wheel thrown coarse ware is a chronological marker defined as post-orientalizing (after 600 BCE) in S2 Table while black gloss pottery indicates a date range during the republican period (3^rd^ and 2^nd^ century BCE). All archaeological dates are presented in S2 Table. Inconsistent dates, out of sequence and rejected as reworked material, are shown in *Italic* and have not been included in the chronostratigraphic analysis illustrated in Fig. 2 of the main text.

**Paleomagnetism**

Following AF demagnetization of the natural remanent magnetization (NRM) , mineral magnetic analyses, based on magnetic coercivity and thermal unblocking characteristics, were conducted on a set of representative discrete samples. From the stepwise AF demagnetization diagrams we derived the median destructive field of the NRM (MDF_NRM_), defined as the field required to reduce the NRM intensity to one-half of its initial value, while ARM/k were calculated as magnetic grain size proxy. Low-field volume specific magnetic susceptibility (k), was measured using the in-line Bartington MS2C susceptibility loop sensor and with the AGICO KLY-3S Kappabridge magnetic susceptibility meter. An anhysteretic remanent magnetization (ARM) was imparted by using a 0.1 mT direct current (DC) bias field superimposed on a 100 mT peak AF and by translating samples through the AF and DC coil system at 10 cm/s. The resulting ARM was then stepwise demagnetized using the same sequence of AF peaks applied to the NRM. Full paleomagnetic data are reported in Fig. B.

**
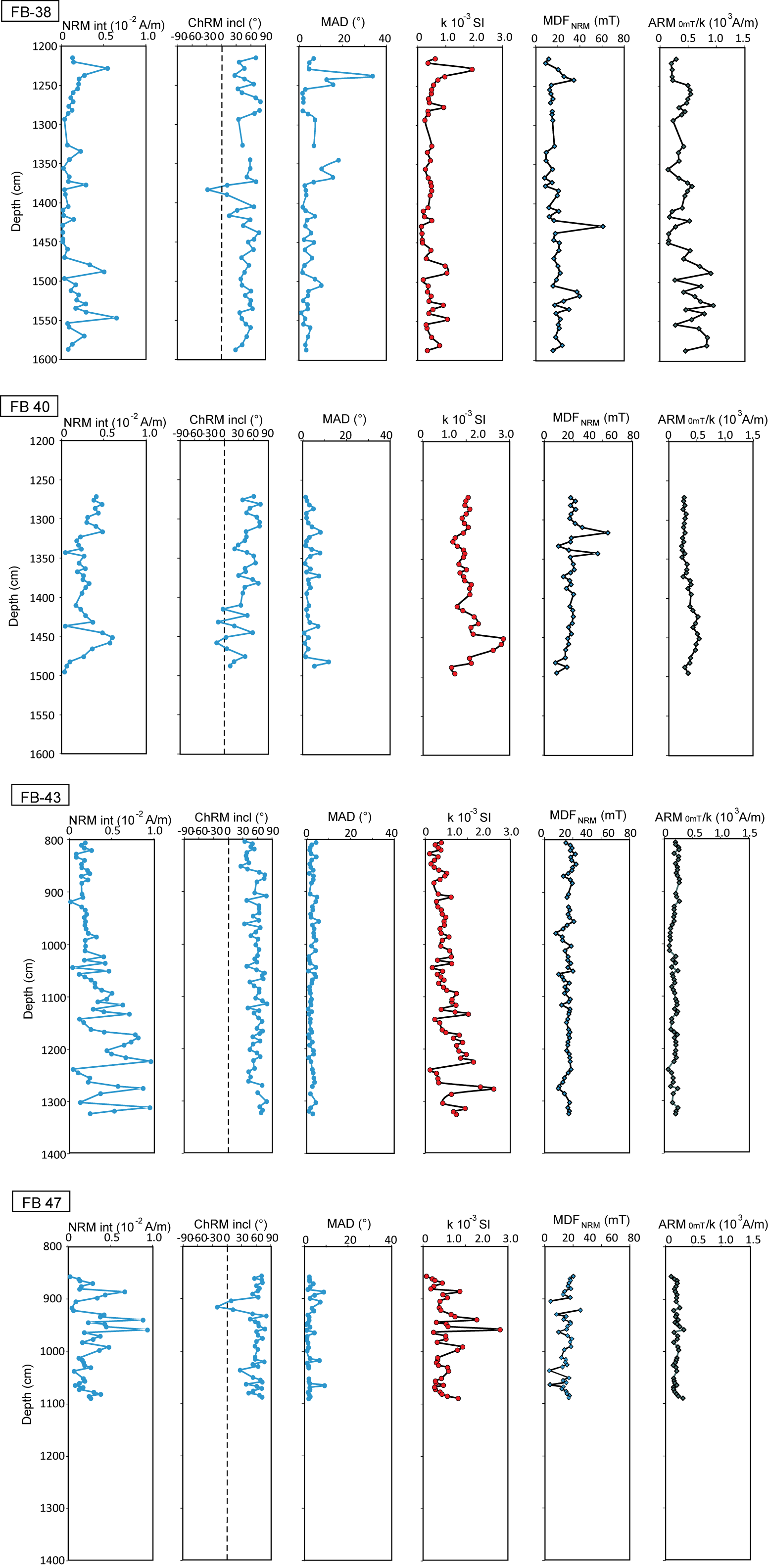
**

Figure B - Measured magnetic parameters.

Downcore variation of natural remanent magnetization at 0 mT (NRM), characteristic remanent magnetization (ChRM) declination and inclination, maximum angular deviation (MAD), magnetic susceptibility (k), anhysteretic remanent magnetization (ARM), median destructive field of the NRM (MDFnrm). See methods section in main text for further explanations.

**
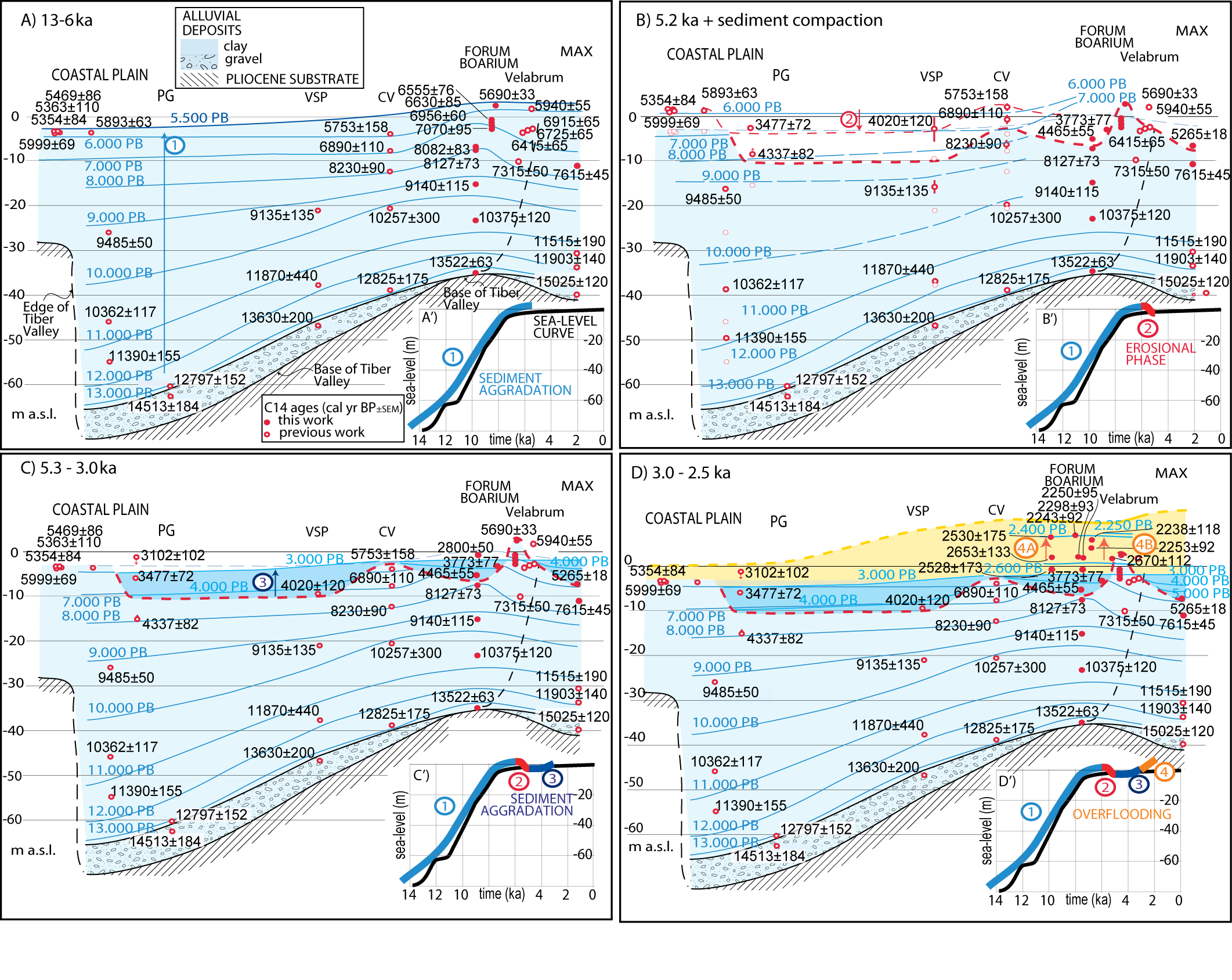
**

Figure C - Aggradational history of the Tiber River. ^14^C age constraints provided by literature data and by the present study to sediment aggradation in the Tiber Valley between Rome and the coastline . A cross-section longitudinal to the river course is reconstructed using all available borehole data (location in Fig. 1). Each panel show the stratigraphic setting at different ages; insets show the aggradation curve of the Tiber sediments (thick colored line) compared to the global sea-level curve from coral reefs data . Different aggradational and erosional phases are numbered 1 to 4.


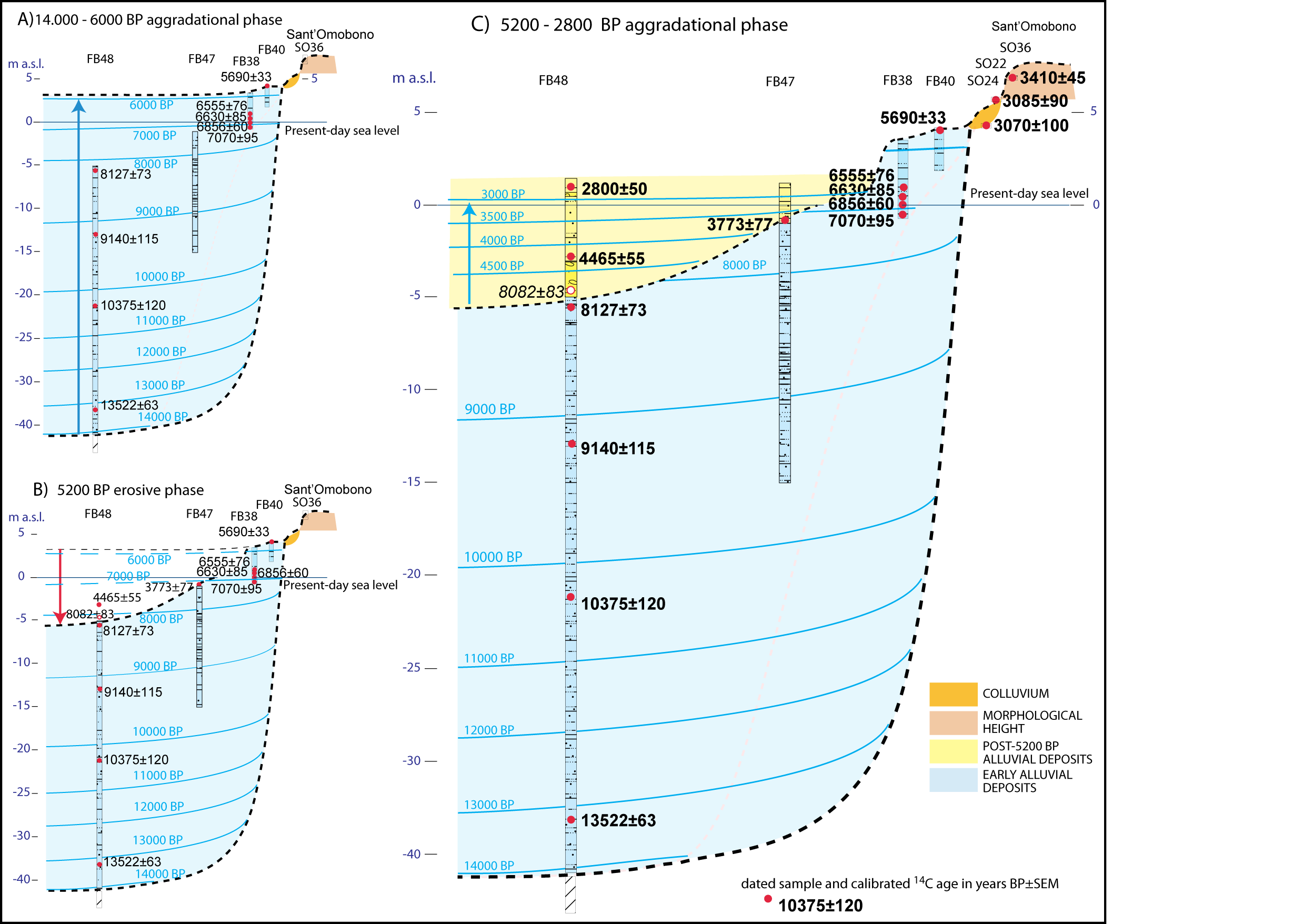


Figure D - Landscape evolution in Forum Boarium. Cross-sections showing the evolution of the landscape in the Forum Boarium area since 6000 yr BP (A), and following the 5200 yr BP erosional phase (B), until the formation of a new alluvial plain around 2800 yr BP (C), based on the reported core chronostratigraphy.

**References**

Bellotti P*, et al.* (2007) Late Quaternary landscape evolution of the Tiber River delta plain (Central Italy): new evidence from pollen data, biostratigraphy and 14C dating. *Zeitschrift fur Geomorphologie* 51(4):505-534.

Belluomini G, Iuzzolini P, Manfra L, Mortari R, & Zalaffi M (1986) Evoluzione recente del delta del Tevere. *Geologica Romana* 25:213-234.

Bozzano, F., Andreucci, A., Gaeta, M., Salucci, R., 2000. A geological model of the buried Tiber River valley beneath the historical centre of Rome. Bull. Ang. Geol. Env.*,* 59, 1-21.

Bozzano, F., Caserta, A., Govoni, A., Marra, F., Martino, S., 2008. Static and dynamic characterisation of alluvial deposits in the Tiber River Valley: new data for assessing potential ground motion in the city of Rome, J.G.R., 113, BO1303, doi:10.1029/2006JB004873.

Corazza, A., Lanzini, M., Rosa, C., Salucci, R, 1999. Caratteri stratigrafici, idrogeologici e geotecnici delle alluvioni tiberine nel settore del centro storico di Roma. Il Quaternario, 12, 215-235.

Marra F, Bozzano F, & Cinti FR (2013) Chronostratigraphic and lithologic features of the Tiber River sediments (Rome, Italy): Implications on the post-glacial sea-level rise and Holocene climate. *Global and Planetary Change* 107(Supplement C):157-176.

Peltier WR & Fairbanks RG (2006) Global glacial ice volume and Last Glacial Maximum duration from an extended Barbados sea level record. *Quaternary Science Reviews* 25(23):3322-3337.
