# Supplementary material for: Rome in its setting. Post-glacial aggradation history of the Tiber River alluvial deposits and tectonic origin of the Tiber Island
Source: PLoS One. 2018 Mar 28;13(3):e0194838. doi: 10.1371/journal.pone.0194838 (PMC5874049; doi:10.1371/journal.pone.0194838)
Supplement: S2 File — All 14C ages are newly reported, except the last four (labelled SO) which have been published previously [3–5]. (DOC) [file pone.0194838.s002.doc]

S2 Table. ^14^C and archaeological dates

| **Core** | **Cut** | **Depth (m)^1^** | **Elevation (masl)^1^** | **Chronological marker** | **Material** | **Calendar year^2^**  **BCE BP^3^** | | **Calibrated**  **^14^C age BP (2σ)** | **^14^C age BP** | **Sample #** | **Lab code** |
| --- | --- | --- | --- | --- | --- | --- | --- | --- | --- | --- | --- |
| FB 38 | 8 | 7.30 | 5.64 | Archaeological | Mortar | After 150 | ≤2100 |  |  |  |  |
|  | 10 | 9.25 | 3.69 | Archaeological | Republican sherd | Late 4^th^-early1^st^ c. | 2300-2050 |  |  |  |  |
|  | *12* | *11.62-11.73* | *1.32 1.21* | *Archaeological* | *Post-archaic sherd* | *4th-1st c.* | *2350-1950* |  |  |  |  |
|  | 12 | 11.72-11.78 | 1.22 to 1.16 | ^14^C | Waterlogged seeds | 795-545 | 2745-2495 | 2620±125 | 2530±30 | 22 | Beta 449392 |
|  | 15 | 14.03-14.43 | -1.09 to -1.49 | ^14^C | Sorted pollen | 4683-4461 | 6633-6411 | 6522±111 | 5716±42 | 31 | DSH 7887 |
|  | 15 | 14.72-14.77 | -1.78 to -1.83 | ^14^C | Waterlogged wood- above ground | 4715-4545 | 6665-5495 | 6580±85 | 5790±30 | 23 | Beta 449393 |
|  | 16 | 15.20-15.24 | -2.26 to -2.30 | ^14^C | Waterlogged wood- above ground | 4934-4727 | 6884-6677 | 66781±103 | 5950±40 | 1 | GrA 66073 |
|  | 16 | 15.73-15.76 | -2.79 to -2.82 | ^14^C | Waterlogged wood-above ground | 5215-5025 | 7165-6975 | 7020±96 | 6170±30 | 2 | Beta 424137 |
| FB 39 | 2 | 1.18 | 11.60 | Archaeological | Republican sherd | Late 4^th^-early1^st^ c. | 2300-2050 |  |  |  |  |
|  | 4 | 3.08 | 9.70 | Archaeological | Republican/Imperial sherd | After 600 | ≤2400 |  |  |  |  |
|  | 5 | 4.55 | 8.23 | Archaeological | Mortar | After 150 | ≤2100 |  |  |  |  |
|  | 7 | 6.70-6.80 | 6.08 to 5.98 | ^14^C | Charcoal | 755-405 | 2705-2355 | 2530±175 | 2440±30 | 3 | Beta 431184 |
|  | 7 | 6.80-6.85 | 5.98 to 5.83 | Archaeological | Iron Age/ orientalizing sherd | 800-600 | 2750-2550 |  |  |  |  |
|  | 8 | 7.10 | 5.68 | Archaeological | Archaic sherd | 600-450 | 2550-2400 |  |  |  |  |

In *Italic* date rejected, reworked material

1) Depth and elevation ranges are for sediment samples that contained the dated material. 2) Radiocarbon ranges are calibrated dates at the 95.4% confidence level based on the calibration dataset IntCal13 (Reimer et al., 2013). 3) BP: before present calculated from 1950

S2 Table. Cont.

| **Core** | **Cut** | **Depth (m)^1^** | **Elevation (masl)^1^** | **Chronological marker** | **Material** | **Calendar year^2^**  **BCE BP^3^** | | **Calibrated**  **^14^C age BP (2σ)** | **^14^C age BP** | **Sample #** | **Lab code** |
| --- | --- | --- | --- | --- | --- | --- | --- | --- | --- | --- | --- |
| FB 39 | 12 | 11.18-11.22 | 1.60 to 1.56 | ^14^C | Bone, mammal | 785-520 | 2735-2470 | 2603±133 | 2500±30 | 24 | Beta 449394 |
|  | 13 | 12.36 | 0.42 | Archaeological | Archaic sherd | 600-450 | 2550-2400 |  |  |  |  |
|  | 15 | 14.25 | -1.47 | ^14^C | Bone, tortoise | 750-405 | 2700-2355 | 2528±173 | 2430±30 | 4 | Beta 424138 |
|  | 15 | 14.34 | -1.56 | Archaeological | Iron Age/ Archaic sherd | 800-500 | 2750-2450 |  |  |  |  |
| FB 40 | 5 | 4.0-4.45 | 12.68 to 12.23 | Archaeological | Imperial sherd | After 30 | ≤1950 |  |  |  |  |
|  | 6 | 5.02 | 11.66 | Archaeological | Republican/Imperial sherd | After 350 | ≤2300 |  |  |  |  |
|  | 6 | 5.75 | 10.93 | Archaeological | Mortar | After 150 | ≤2100 |  |  |  |  |
|  | 7 | 6.48 | 10.20 | Archaeological | Post-Orientalizing sherd | After 600 | ≤2550 |  |  |  |  |
|  | 9 | 8.42-8.52 | 8.26 to 8.16 | Archaeological | Etrusco-Corinthian pottery | 700-500 | 2650-2450 |  |  |  |  |
|  | *10* | *9.00-9.10* | *7.68-7.58* | *^14^C* | *Charcoal* | *1050-910* | *3000-2860* | *2930±70* | *2830±30* | *5* | *Beta 422662* |
|  | 10 | 9.82 | 6.86 | Archaeological | Orientalizing/Archaic sherd | 700-500 | 2650-2450 |  |  |  |  |
|  | 10 | 9.85-10 | 6.83-6.68 | Archaeological | Archaic sherd | 600-500 | 2550-2450 |  |  |  |  |
|  | 11 | 10.50-10.60 | 6.18 to 6.08 | Archaeological | Orientalizing/Archaic sherd | 700-500 | 2650-2450 |  |  |  |  |
|  | 12 | 11.20-11.30 | 5.48 to 5.38 | Archaeological | Iron Age sherd | 900-700 | 2850-2650 |  |  |  |  |
|  | 12 | 11.30-11.40 | 5.38 to 5.28 | Archaeological | Iron Age sherd | 800-700 | 2750-2650 |  |  |  |  |
|  | 12 | 11,40 | 5.28 | Archaeological | Bucchero | 700-500 | 2650-2450 |  |  |  |  |

S2 Table. Cont.

| **Core** | **Cut** | **Depth (m)^1^** | **Elevation (masl)^1^** | **Chronological marker** | **Material** | **Calendar year^2^**  **BCE BP^3^** | | **Calibrated**  **^14^C age BP (2σ)** | **^14^C age BP** | **Sample #** | **Lab code** |
| --- | --- | --- | --- | --- | --- | --- | --- | --- | --- | --- | --- |
| FB 40 | 13 | 12.40-12.50 | 4.28 to 4.18 | Archaeological | Iron Age/ Orientalizing sherd | 800-600 | 2750-2550 |  |  |  |  |
|  | 13 | 12.74 | 3.94 | ^14^C | Sorted pollen | 3703-3693 | 5653-5589 | 5621±32 | 4879±28 | 30 | DSH 7836 |
| FB 42 | 3 | 2.90 | 11.32 | Archaeological | Mortar | After 150 | ≤2100 |  |  |  |  |
| FB 43 | 4 | 3.92 | 9.78 | Archaeological | Mortar | After 150 | ≤2100 |  |  |  |  |
|  | 5 | 4.90 | 8.80 | Archaeological | Republican sherd | Late 4^th^-early 1^st^ c. | 2300-2050 |  |  |  |  |
|  | 6 | 5.57-5.75 | 8.13 to 7.95 | Archaeological | Archaic sherd | 600-450 | 2550-2400 |  |  |  |  |
|  | 6 | 5.65-5.85 | 8.05 to 7.85 | Archaeological | Archaic sherd | 600-500 | 2550-2450 |  |  |  |  |
|  | 15 | 14.80-14.85 | -1.10 to -1.15 | ^14^C | Plant material | 795-550 | 2745-2500 | 2622±123 | 2540±30 | 6 | Beta 424139 |
|  | 16 | 15.05 | -1.35 | Archaeological | Archaic sherd | 600-450 | 2550-2400 |  |  |  |  |
| FB 44 | 8 | 7.80 | 9.38 | Archaeological | Republican sherd | After 300 | ≤2250 |  |  |  |  |
|  | 10 | 9.38 | 7.80 | Archaeological | Mortar | After 150 | ≤2100 |  |  |  |  |
|  | 10 | 9.95 | 7.23 | Archaeological | Republican sherd | 300-200 | 2250-2150 |  |  |  |  |
|  | 11 | 10.09-10.26 | 7.09 to 6.92 | Archaeological | Republican sherd | After 250 | ≤2200 |  |  |  |  |
|  | *11* | *10.34* | *6.84* | *Archaeological* | *Archaic Sherd* | *600-500* | *2550-2450* |  |  |  |  |
|  | 11 | 10.55-10-83 | 6.63 to 6.35 | Archaeological | Republican sherd | After 250 | ≤2200 |  |  |  |  |
|  | 15 | 14.37-14.42 | 2.81 to 2.76 | Archaeological | Post-archaic sherd | 500-300 | 2450-2250 |  |  |  |  |
|  | *15* | *14.67* | *2.51* | *Archaeological* | *Iron Age/Archaic sherd* | *800-500* | *2750-2450* |  |  |  |  |

S2 Table. Cont.

| **Core** | **Cut** | **Depth (m)^1^** | **Elevation (masl)^1^** | **Chronological marker** | **Material** | **Calendar year^2^**  **BCE BP^3^** | | **Calibrated**  **^14^C age BP (2σ)** | **^14^C age BP** | **Sample #** | **Lab code** |
| --- | --- | --- | --- | --- | --- | --- | --- | --- | --- | --- | --- |
| FB 44 | *15* | *14.70-14.77* | *2.48 to 2.41* | *Archaeological* | *Archaic sherd* | *600-400* | *2550-2350* |  |  |  |  |
|  | 16 | 15.86-15.90 | 1.32 to 1.28 | ^14^C | Waterlogged wood- above ground | 395-205 | 2345-2155 | 2250±95 | 2250±30 | 7 | Beta 431188 |
| FB 45 | 6 | 5.15 | 12.77 | Archaeological | Mortar | After 150 | ≤2100 |  |  |  |  |
|  | 6 | 5.26 | 12.66 | Archaeological | Republican/Imperial sherd | After 350 | ≤2300 |  |  |  |  |
|  | 9 | 8.54 | 9.38 | Archaeological | Post-archaic sherd | 500-200 | 2450-2150 |  |  |  |  |
|  | 10 | 9.35-9.40 | 8.57 to 8.52 | ^14^C | Charcoal | 390-205 | 2340-2155 | 2248±92 | 2240±30 | 8 | Beta 431187 |
|  | 13 | 12.37 | 5.55 | Archaeological | Republican sherd | Late 4^th^-early 1^st^ c. | 2300-2050 |  |  |  |  |
| FB 46 | 13 | 12.55 | 5.85 | Archaeological | Mortar | After 150 | ≤2100 |  |  |  |  |
|  | 13 | 12.85 | 5.55 | Archaeological | Republican sherd | Late 4^th^ -early 1^st^ c. | 2300-2050 |  |  |  |  |
|  | 13 | 12.84-12.92 | 5.56 to 5.48 | ^14^C | Bone, cattle | 355-120 | 2305-2070 | 2187±118 | 2170±30 | 25 | Beta 449396 |
|  | 15 | 14.40-14.50 | 4.0 to 3.90 | Archaeological | Republican sherd | After 350 | ≤2300 |  |  |  |  |
|  | 15 | 14.67-14.80 | 3.73 to 3.60 | ^14^C | Charred seed | 395-210 | 2345-2160 | 2253±92 | 2270±30 | 9 | Beta 431185 |
|  | 15 | 14.80-14.90 | 3.60 to 3.50 | Archaeological | Archaic sherd | 600-450 | 2550-2400 |  |  |  |  |
|  | 15 | 14.90-14.98 | 3.50 to 3.42 | Archaeological | Archaic sherd | 650-500 | 2600-2450 |  |  |  |  |
| FB 47 | *4* | *3.24-3.31* | *10.19 to 10.12* | *Archaeological* | *Post-Orientalizing sherd* | *After 600* | *≤2550* |  |  |  |  |
|  | 6 | 5.42 | 8.01 | Archaeological | Mortar | After 150 | ≤2100 |  |  |  |  |
|  | 7 | 6.00 | 7.43 | Archaeological | Late Archaic building phase | ca. 480 | ̴ 2430 |  |  |  |  |

S2 Table. Cont.

| **Core** | **Cut** | **Depth (m)^1^** | **Elevation (masl)^1^** | **Chronological marker** | **Material** | **Calendar year^2^**  **BCE BP^3^** | | **Calibrated**  **^14^C age BP (2σ)** | **^14^C age BP** | **Sample #** | **Lab code** |
| --- | --- | --- | --- | --- | --- | --- | --- | --- | --- | --- | --- |
| FB 47 | 7 | 6.54 | 6.89 | Archaeological | Post-Orientalizing sherd | After 600 | ≤2550 |  |  |  |  |
|  | 9 | 8.50 | 4.93 | Archaeological | Late Archaic building phase | ca. 480 | ̴ 2430 |  |  |  |  |
|  | *9* | *8.80-8.90* | *4.63 to 4.53* | *^14^C* | *Plant material* | *1625-1500* | *3575-3450* | *3513±63* | *3280 ±30* | *10* | *Beta 422664* |
|  | *10* | *9.60* | *3.83* | *^14^C* | *Organic sediment* | *3355-3095* | *5305-5045* | *5175±130* | *4510 ±30* | *12* | *Beta 430834* |
|  | *12* | *11.65-11.70* | *1.78 to 1.73* | *^14^C* | *Plant material* | *805-765* | *2755-2715* | *2735±20* | *2580 ±30* | *11* | *Beta 422665* |
|  | *14* | *13.85* | *-0.42* | *Archaeological* | *Iron Age sherd* | *800-700* | *2750-2650* |  |  |  |  |
|  | 14 | 13.80-14 | -0.37 to -0.57 | Archaeological | Iron Age/ Orientalizing sherd | 800-600 | 2750-2550 |  |  |  |  |
|  | 15 | 14.10-14.13 | -0.67 to -0.70 | Archaeological | Archaic sherd | 600-450 | 2550-2400 |  |  |  |  |
|  | 15 | 14.15-14.25 | -0.72 to -0.82 | Archaeological | Archaic sherd | 600-450 | 2550-2400 |  |  |  |  |
|  | 17 | 16.55 | -3.12 | ^14^C | Plant material | 1900-1745 | 3850-3695 | *3773±77* | 3500±30 | 13 | Beta 422666 |
| FB 48 | 1 | 0.45 | 13.04 | Archaeological | Imperial sherd | After 50CE | ≤1900 |  |  |  |  |
|  | 6 | 5.85 | 7.64 | Archaeological | Mortar | After 150 | ≤2100 |  |  |  |  |
|  | 7 | 6.80-7.0 | 6.69 to 6.49 | ^14^C | Charred seeds | 385 - 200 | 2335-2150 | 2243±92 | 2230±30 | 14 | Beta 431186 |
|  | 8 | 7.10 | 6.39 | Archaeological | Republican sherd | 350 - 100 | 2300-2050 |  |  |  |  |
|  | *8* | *7.05-7.30* | *6.44 to 6.19* | *Archaeological* | *Archaic/Republican sherd* | *After 600* | *≤2550* |  |  |  |  |
|  | *8* | *7.35* | *6.14* | *Archaeological* | *Iron Age/ Orientalizing sherd* | *800-600* | *2750-2550* |  |  |  |  |
|  | *8* | *7.65* | *5.84* | *Archaeological* | *Archaic/post-archaic* | *600-300* | *2550-2250* |  |  |  |  |

S2 Table. Cont.

| **Core** | **Cut** | **Depth (m)^1^** | **Elevation (masl)^1^** | **Chronological marker** | **Material** | **Calendar year^2^**  **BCE BP^3^** | | **Calibrated**  **^14^C age BP (2σ)** | **^14^C age BP** | **Sample #** | **Lab code** |
| --- | --- | --- | --- | --- | --- | --- | --- | --- | --- | --- | --- |
| FB 48 | *9* | *8.3* | *5.19* | *Archaeological* | *Archaic/post-archaic* | *600-300* | *2550-2250* |  |  |  |  |
|  | *9* | *8.84* | *4.65* | *Archaeological* | *Archaic/post-archaic* | *600-300* | *2550-2250* |  |  |  |  |
|  | *12* | *11.05* | *2.44* | *Archaeological* | *Iron Age/ Orientalizing sherd* | *800-600* | *2750-2550* |  |  |  |  |
|  | 12 | 11.86 | 1.63 | ^14^C | Waterlogged wood- above ground | 390-205 | 2340-2155 | 2298±93 | 2240±30 | 26 | Beta 449395 |
|  | 13 | 12.2 | 1.29 | Archaeological | Republican sherd | Late 4^th^-early 1^st^ c. | 2300-2050 |  |  |  |  |
|  | 15 | 14.08 | -0.59 | Archaeological | Iron Age/ Orientalizing sherd | 800-600 | 2750-2550 |  |  |  |  |
|  | 15 | 14.70-14.71 | -1.21 | ^14^C | Plant material | 900-800 | 2850-2750 | 2800±50 | 2690±30 | 15 | Beta 424140 |
|  | 19 | 18.6 | -5.11 | ^14^C | Carbonized plant material | 2570-2460 | 4520-4410 | 4465±55 | 3980±30 | 16 | Beta 430836 |
|  | 20 | 20 | -6.51 | ^14^C | Plant material | 6215-6050 | 8165-8000 | 8082±83 | 7250±30 | 17 | Beta 422668 |
|  | 21 | 21 | -7.51 | ^14^C | Plant material | 6250-6105 | 8200-8055 | 8127±73 | 7350±30 | 18 | Beta 422669 |
|  | 29 | 28.4 | -14.91 | ^14^C | Plant material | 7305-7075 | 9255-9025 | 9140±115 | 8180±30 | 19 | Beta 430835 |
|  | 37 | 36.7 | -23.21 | ^14^C | Plant material | 8545-8305 | 10495-10255 | 1037±120 | 9210±30 | 20 | Beta 422670 |
|  | 49 | 48.60 | -35.11 | ^14^C | Carbonized plant material | 11635-11510 | 13585-13460 | 13522±63 | 11720±40 | 21 | Beta 425692 |
| FB 49 | 17 | 16.25-16.50 | 1.40 to 1.15 | Archaeological | Republican/Imperial sherd | After 350 | ≤2300 |  |  |  |  |
|  | 19 | 18.70 | -1.05 | Archaeological | Mortar | After 150 | ≤2100 |  |  |  |  |
|  | 19 | 18.77 | -1.12 | Archaeological | Orientalizing/Archaic sherd | 700-500 | 2650-2450 |  |  |  |  |

S2 Table. Cont.

| **Core** | **Cut** | **Depth (m)^1^** | **Elevation (masl)^1^** | **Chronological marker** | **Material** | **Calendar year^2^**  **BCE BP^3^** | | **Calibrated**  **^14^C age BP (2σ)** | **^14^C age BP** | **Sample #** | **Lab code** |
| --- | --- | --- | --- | --- | --- | --- | --- | --- | --- | --- | --- |
| FB 49 | 19 | 18.95 | -1.30 | Archaeological | Archaic sherd | 600-450 | 2550-2400 |  |  |  |  |
| *Maxii* | *S3 C2* | *21.0* | *-5.00* | *^14^C* | *Charred plant material* | *4455-4345* | *6405-6295* | *6350±55* | *5570*±30 | *29* | *Beta 422672* |
|  | S3 C5 | 22.8 | -6.80 | ^14^C | Wood | 3490-3140 | 5440-5090 | 5265±175 | 4580±30 | 27 | Beta 422673 |
|  | S3 C6 | 26.8 | -10.80 | ^14^C | Wood | 5710-5620 | 7660-7570 | 7615±45 | 6740±30 | 28 | Beta 430837 |
| SO22 | 2 | 1.66-1.83 | 5.79 to 5.62 | ^14^C | Charred seed | 1225-1045 | 3175-2995 | 3085±90 | 2940±30 |  | Beta 393853 |
|  | 3 | 2.25-2.47 | 5.20 to 4.98 | ^14^C | Charred seed | 1205-1005 | 3155-2955 | 3055±100 | 2900±30 |  | Beta 393850 |
| SO24 | 4 | 4.11-4.20 | 3.28 to 3.19 | ^14^C | Charred seed | 1115-930 | 3065-2880 | 2972±93 | 2860±30 |  | Beta 393852 |
|  | 4 | 4.25-4.36 | 3.14 to 3.03 | ^14^C | Charred seed | 1220-1020 | 3170-2970 | 3070±100 | 2930±30 |  | Beta 393851 |
| SO32 | 5 | 5.69 | 7.17 | Archaeological | Iron Age sherd | 800-700 | 2750-2650 |  |  |  |  |
| SO36 | 5 | 4.77-4.87 | 7.04 to 6.94 | ^14^C | Charcoal | 1505-1415 | 3455-3365 | 3410±45 | 3190±30 |  | Beta 393854 |
