# Supplementary material for: Rome in its setting. Post-glacial aggradation history of the Tiber River alluvial deposits and tectonic origin of the Tiber Island
Source: PLoS One. 2018 Mar 28;13(3):e0194838. doi: 10.1371/journal.pone.0194838 (PMC5874049; doi:10.1371/journal.pone.0194838)
Supplement: S3 File — (PDF) [file pone.0194838.s003.pdf]

CALIBRATION OF RADIOCARBON AGE TO CALENDAR YEARS

(Variables: C13/C12 = -21.8 o/oo : lab. mult = 1)

**Laboratory number**	**Beta-393850**
**Conventional radiocarbon age**	**2900 ± 30 BP**
**2 Sigma calibrated result**<br>**95% probability**	**Cal BC 1205 to 1140 (Cal BP 3155 to 3090)**<br>**Cal BC 1130 to 1005 (Cal BP 3080 to 2955)**
Intercept of radiocarbon age with calibration curve	Cal BC 1105 (Cal BP 3055)<br>Cal BC 1100 (Cal BP 3050)<br>Cal BC 1080 (Cal BP 3030)<br>Cal BC 1075 (Cal BP 3025)<br>Cal BC 1065 (Cal BP 3015)<br>Cal BC 1055 (Cal BP 3005)
1 Sigma calibrated results<br>68% probability	Cal BC 1120 to 1020 (Cal BP 3070 to 2970)

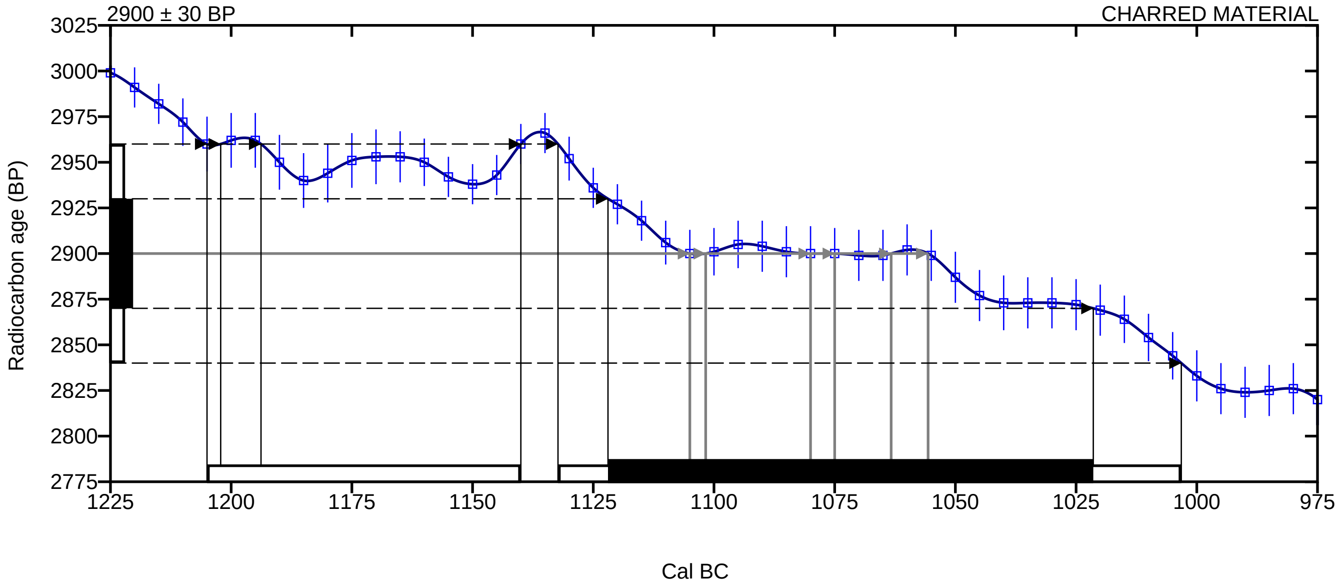

Database used

INTCAL13

References

Mathematics used for calibration scenario

A Simplified Approach to Calibrating C14 Dates, Talma, A. S., Vogel, J. C., 1993, Radiocarbon 35(2):317-322

References to INTCAL13 database

Reimer PJ et al. IntCal13 and Marine13 radiocarbon age calibration curves 0–50,000 years cal BP. Radiocarbon 55(4):1869–1887.

CALIBRATION OF RADIOCARBON AGE TO CALENDAR YEARS

(Variables: C13/C12 = -22.8 o/oo : lab. mult = 1)

**Laboratory number**	**Beta-393851**
**Conventional radiocarbon age**	**2930 ± 30 BP**
**2 Sigma calibrated result<br>95% probability**	**Cal BC 1220 to 1020 (Cal BP 3170 to 2970)**
Intercept of radiocarbon age with calibration curve	Cal BC 1120 (Cal BP 3070)
1 Sigma calibrated results<br>68% probability	Cal BC 1205 to 1140 (Cal BP 3155 to 3090)<br>Cal BC 1130 to 1075 (Cal BP 3080 to 3025)<br>Cal BC 1065 to 1055 (Cal BP 3015 to 3005)

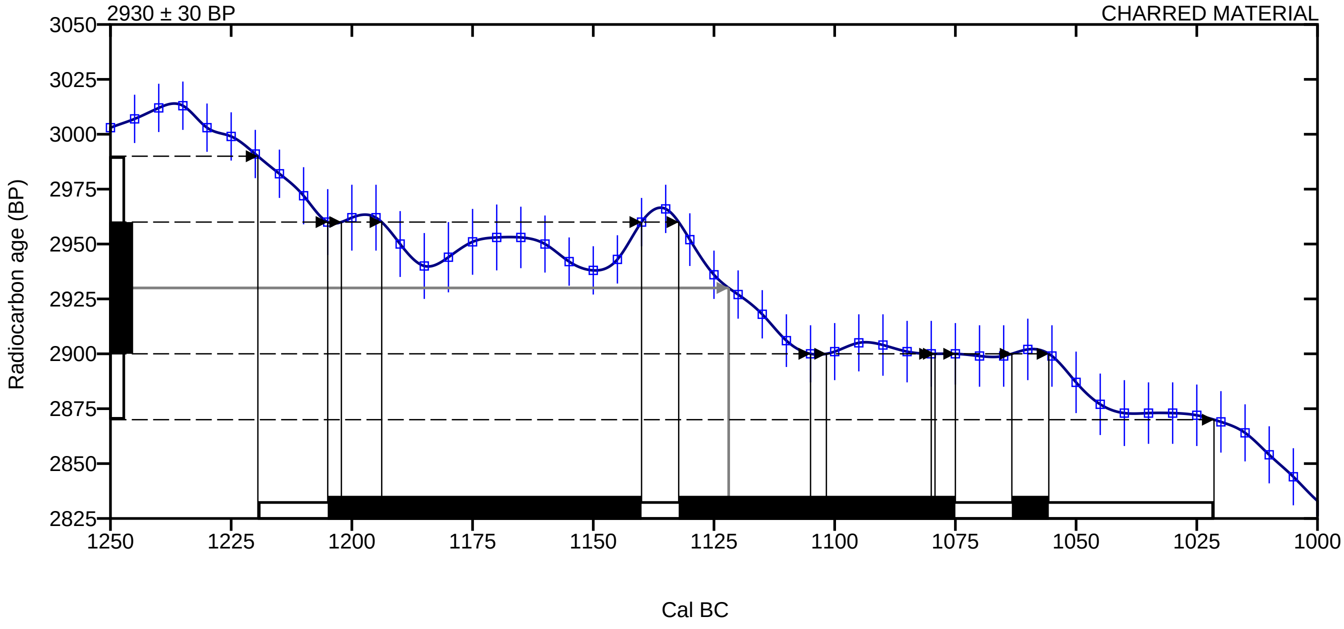

Database used

INTCAL13

References

Mathematics used for calibration scenario

A Simplified Approach to Calibrating C14 Dates, Talma, A. S., Vogel, J. C., 1993, Radiocarbon 35(2):317-322

References to INTCAL13 database

Reimer PJ et al. IntCal13 and Marine13 radiocarbon age calibration curves 0– 50,000 years cal BP. Radiocarbon 55(4):1869– 1887.

Beta Analytic Radiocabon Dating Laboratory

4985 S.W. 74th Court, Miami, Florida 33155 • Tel: (305)667-5167 • Fax: (305)663-0964 • Email: beta@radiocarbon.com

CALIBRATION OF RADIOCARBON AGE TO CALENDAR YEARS

(Variables: C13/C12 = -22.9 o/oo : lab. mult = 1)

**Laboratory number**	**Beta-393852**
**Conventional radiocarbon age**	**2860 ± 30 BP**
**2 Sigma calibrated result**<br>**95% probability**	**Cal BC 1115 to 970 (Cal BP 3065 to 2920)**<br>**Cal BC 960 to 930 (Cal BP 2910 to 2880)**
Intercept of radiocarbon age with calibration curve	Cal BC 1015 (Cal BP 2965)
1 Sigma calibrated results<br>68% probability	Cal BC 1050 to 1000 (Cal BP 3000 to 2950)

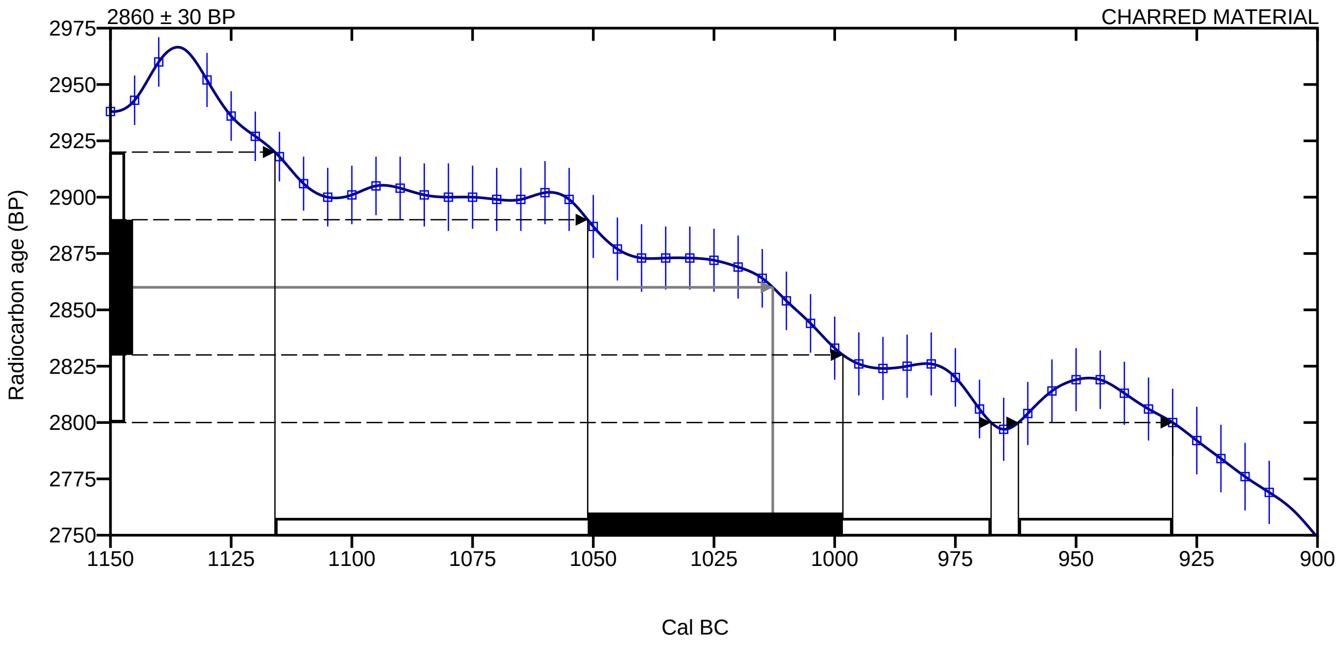

Database used
INTCAL13

References
Mathematics used for calibration scenario

A Simplified Approach to Calibrating C14 Dates, Talma, A. S., Vogel, J. C., 1993, Radiocarbon 35(2):317-322

References to INTCAL13 database

Reimer PJ et al. IntCal13 and Marine13 radiocarbon age calibration curves 0– 50,000 years cal BP. Radiocarbon 55(4):1869– 1887.

4985 S.W. 74th Court, Miami, Florida 33155 • Tel: (305)667-5167 • Fax: (305)663-0964 • Email: beta@radiocarbon.com

CALIBRATION OF RADIOCARBON AGE TO CALENDAR YEARS

(Variables: C13/C12 = -23.4 o/oo : lab. mult = 1)

**Laboratory number**	**Beta-393853**
**Conventional radiocarbon age**	**2940 ± 30 BP**
**2 Sigma calibrated result**<br>**95% probability**	**Cal BC 1225 to 1045 (Cal BP 3175 to 2995)**
Intercept of radiocarbon age with calibration curve	Cal BC 1185 (Cal BP 3135)<br>Cal BC 1155 (Cal BP 3105)<br>Cal BC 1145 (Cal BP 3095)<br>Cal BC 1125 (Cal BP 3075)
1 Sigma calibrated results<br>68% probability	Cal BC 1210 to 1110 (Cal BP 3160 to 3060)

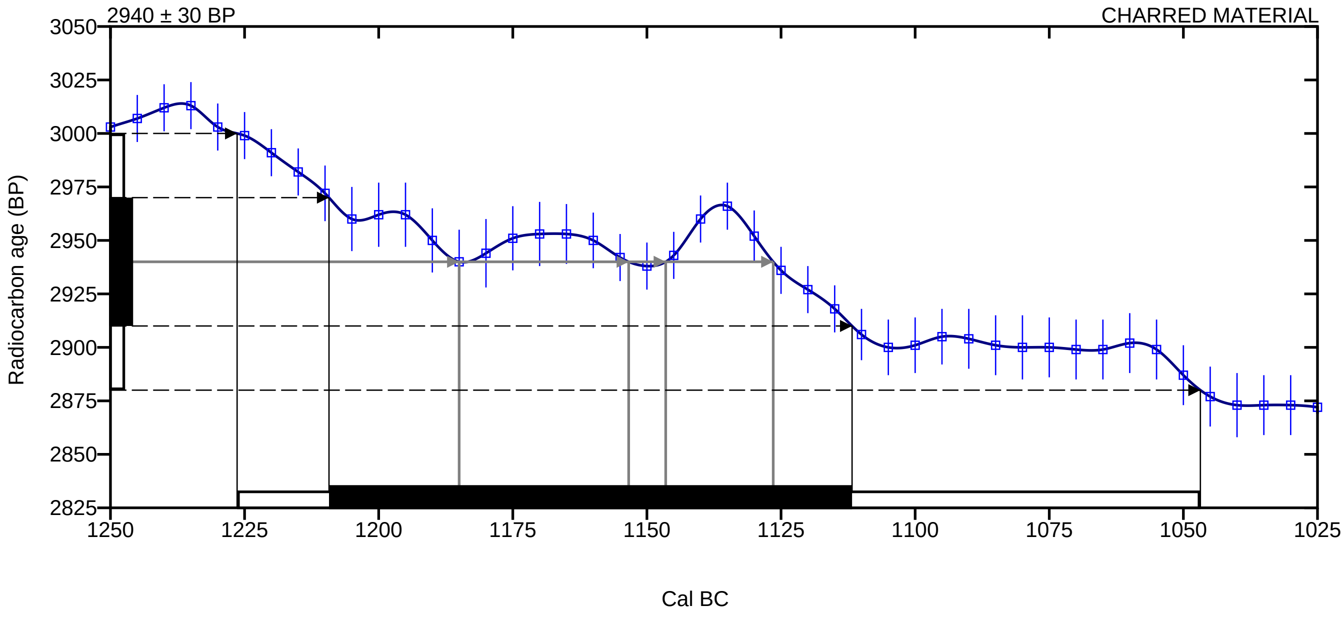

Database used

INTCAL13

References

Mathematics used for calibration scenario

A Simplified Approach to Calibrating C14 Dates, Talma, A. S., Vogel, J. C., 1993, Radiocarbon 35(2):317-322

References to INTCAL13 database

Reimer PJ et al. IntCal13 and Marine13 radiocarbon age calibration curves 0–50,000 years cal BP. Radiocarbon 55(4):1869–1887.

Beta Analytic Radiocabon Dating Laboratory

4985 S.W. 74th Court, Miami, Florida 33155 • Tel: (305)667-5167 • Fax: (305)663-0964 • Email: beta@radiocarbon.com

CALIBRATION OF RADIOCARBON AGE TO CALENDAR YEARS

(Variables: C13/C12 = -26.1 o/oo : lab. mult = 1)

**Laboratory number**	**Beta-393854**
**Conventional radiocarbon age**	**3190 ± 30 BP**
**2 Sigma calibrated result 95% probability**	**Cal BC 1505 to 1415 (Cal BP 3455 to 3365)**
Intercept of radiocarbon age with calibration curve	Cal BC 1450 (Cal BP 3400)
1 Sigma calibrated results 68% probability	Cal BC 1500 to 1430 (Cal BP 3450 to 3380)

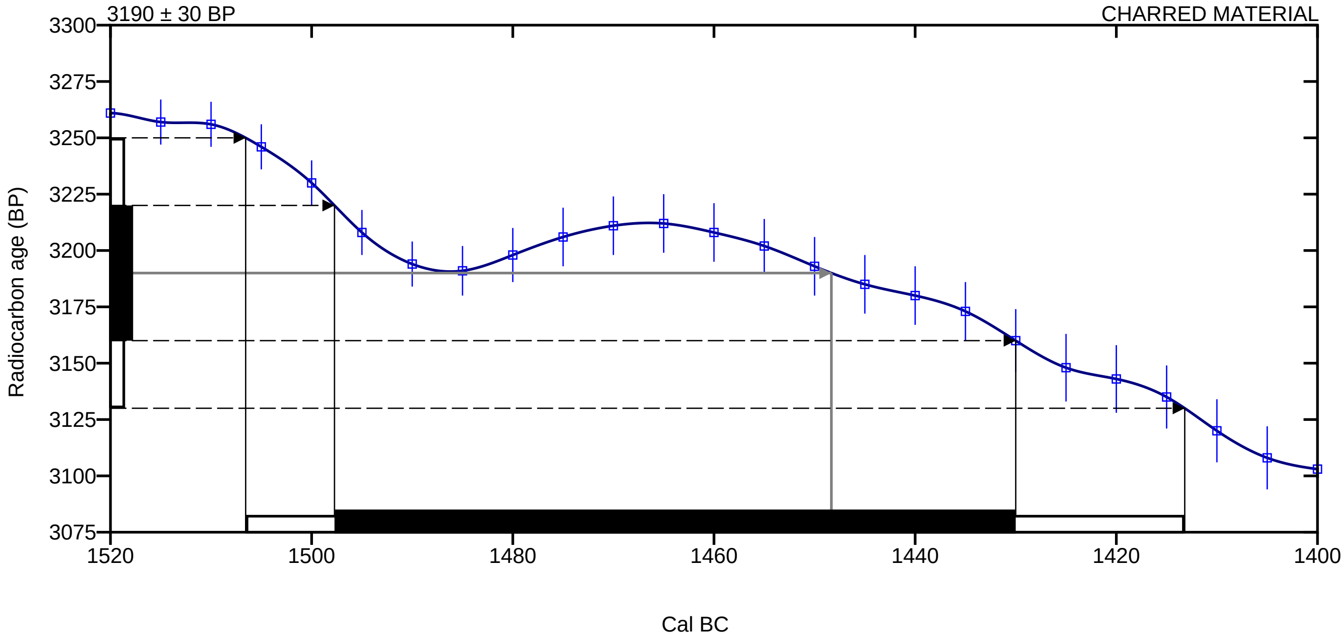

Database used
INTCAL13

References

Mathematics used for calibration scenario
A Simplified Approach to Calibrating C14 Dates, Talma, A. S., Vogel, J. C., 1993, Radiocarbon 35(2):317-322
References to INTCAL13 database
Reimer PJ et al. IntCal13 and Marine13 radiocarbon age calibration curves 0–50,000 years cal BP. Radiocarbon 55(4):1869–1887.

Beta Analytic Radiocabon Dating Laboratory
4985 S.W. 74th Court, Miami, Florida 33155 • Tel: (305)667-5167 • Fax: (305)663-0964 • Email: beta@radiocarbon.com

CALIBRATION OF RADIOCARBON AGE TO CALENDAR YEARS

(Variables: C13/C12 = -23.8 o/oo : lab. mult = 1)

**Laboratory number**	**Beta-431184 : FB 39-7**
**Conventional radiocarbon age**	**2440 ± 30 BP**
**Calibrated Result (95% Probability)**	**Cal BC 755 to 680 (Cal BP 2705 to 2630)**<br>**Cal BC 670 to 610 (Cal BP 2620 to 2560)**<br>**Cal BC 595 to 405 (Cal BP 2545 to 2355)**
Intercept of radiocarbon age with calibration curve	Cal BC 535 (Cal BP 2485)<br>Cal BC 525 (Cal BP 2475)<br>Cal BC 520 (Cal BP 2470)
Calibrated Result (68% Probability)	Cal BC 735 to 690 (Cal BP 2685 to 2640)<br>Cal BC 660 to 645 (Cal BP 2610 to 2595)<br>Cal BC 545 to 415 (Cal BP 2495 to 2365)

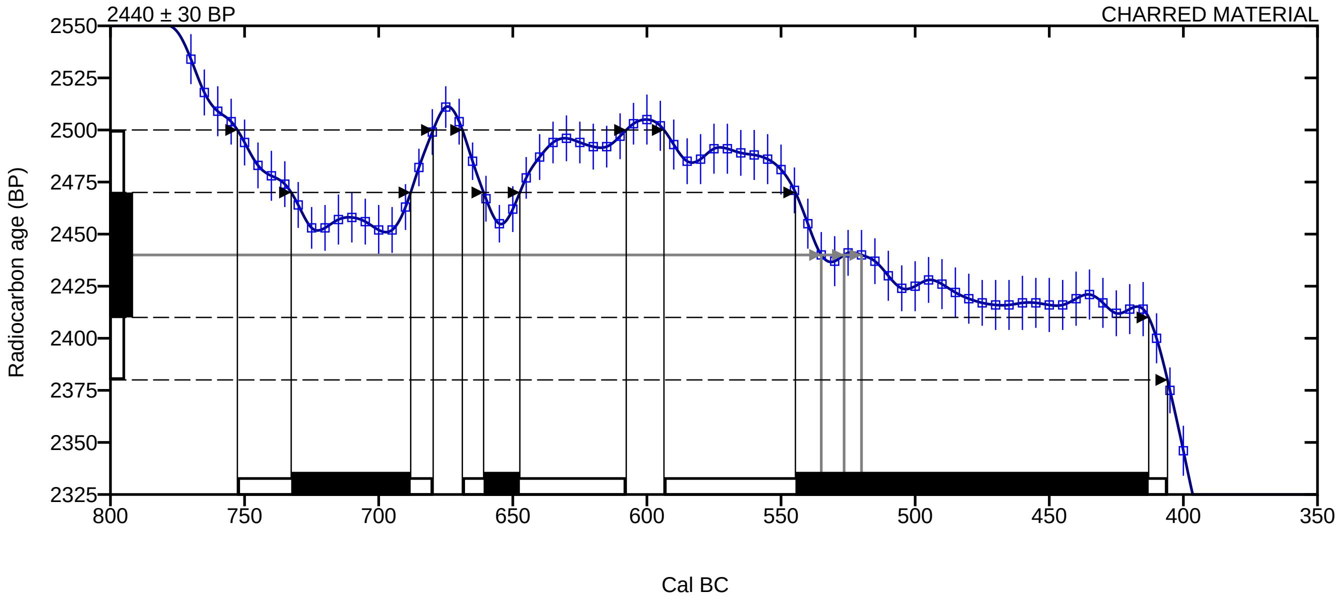

Database used

INTCAL13

References

Mathematics used for calibration scenario

A Simplified Approach to Calibrating C14 Dates, Talma, A. S., Vogel, J. C., 1993, Radiocarbon 35(2):317-322

References to INTCAL13 database

Reimer PJ et al. IntCal13 and Marine13 radiocarbon age calibration curves 0– 50,000 years cal BP. Radiocarbon 55(4):1869– 1887., 2013.

(Variables: C13/C12 = -23.3 o/oo : lab. mult = 1)

Laboratory number **Beta-431185 : FB 46-15**

Conventional radiocarbon age **2270 ± 30 BP**

Calibrated Result (95% Probability)
Cal BC 395 to 350 (Cal BP 2345 to 2300)
Cal BC 295 to 230 (Cal BP 2245 to 2180)
Cal BC 220 to 210 (Cal BP 2170 to 2160)

Intercept of radiocarbon age with calibration curve Cal BC 375 (Cal BP 2325)

Calibrated Result (68% Probability) Cal BC 390 to 360 (Cal BP 2340 to 2310)

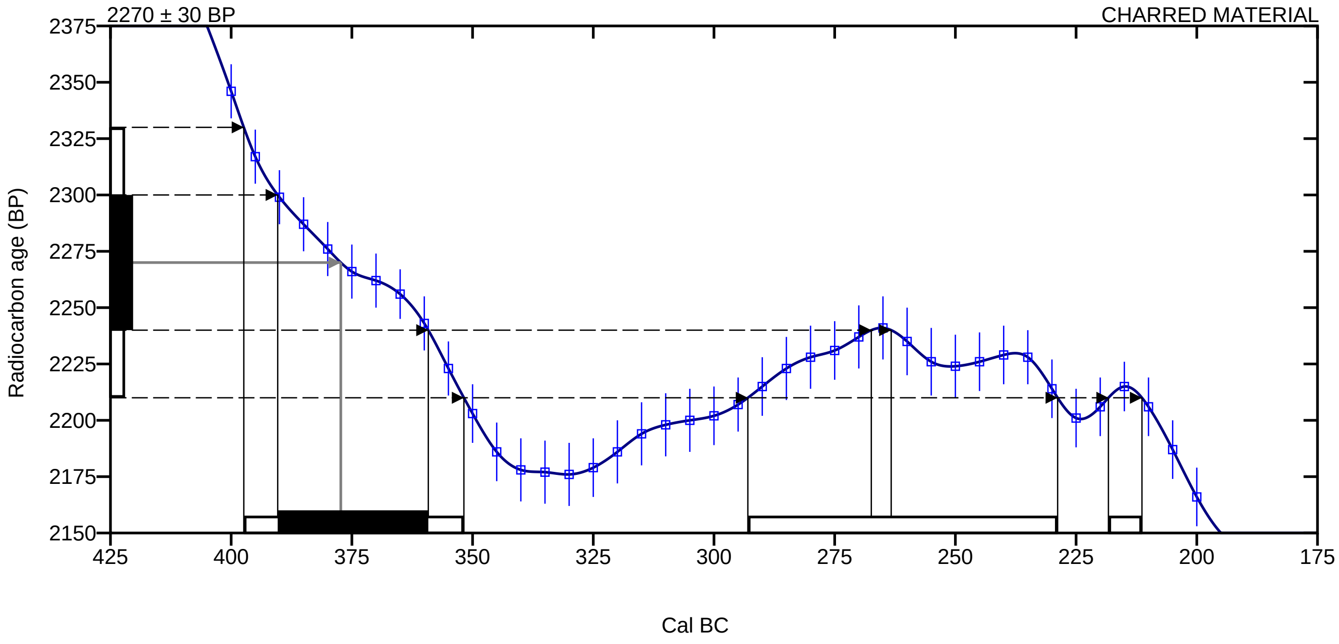

Database used

INTCAL13

References

Mathematics used for calibration scenario

A Simplified Approach to Calibrating C14 Dates, Talma, A. S., Vogel, J. C., 1993, Radiocarbon 35(2):317-322

References to INTCAL13 database

Reimer PJ et al. IntCal13 and Marine13 radiocarbon age calibration curves 0– 50,000 years cal BP. Radiocarbon 55(4):1869– 1887., 2013.

CALIBRATION OF RADIOCARBON AGE TO CALENDAR YEARS

(Variables: C13/C12 = -22.8 o/oo : lab. mult = 1)

**Laboratory number**	**Beta-431186 : FB 48-7**
**Conventional radiocarbon age**	**2230 ± 30 BP**
**Calibrated Result (95% Probability)**	**Cal BC 385 to 200 (Cal BP 2335 to 2150)**
Intercept of radiocarbon age with calibration curve	Cal BC 355 (Cal BP 2305)<br>Cal BC 275 (Cal BP 2225)<br>Cal BC 255 (Cal BP 2205)
Calibrated Result (68% Probability)	Cal BC 370 to 350 (Cal BP 2320 to 2300)<br>Cal BC 305 to 210 (Cal BP 2255 to 2160)

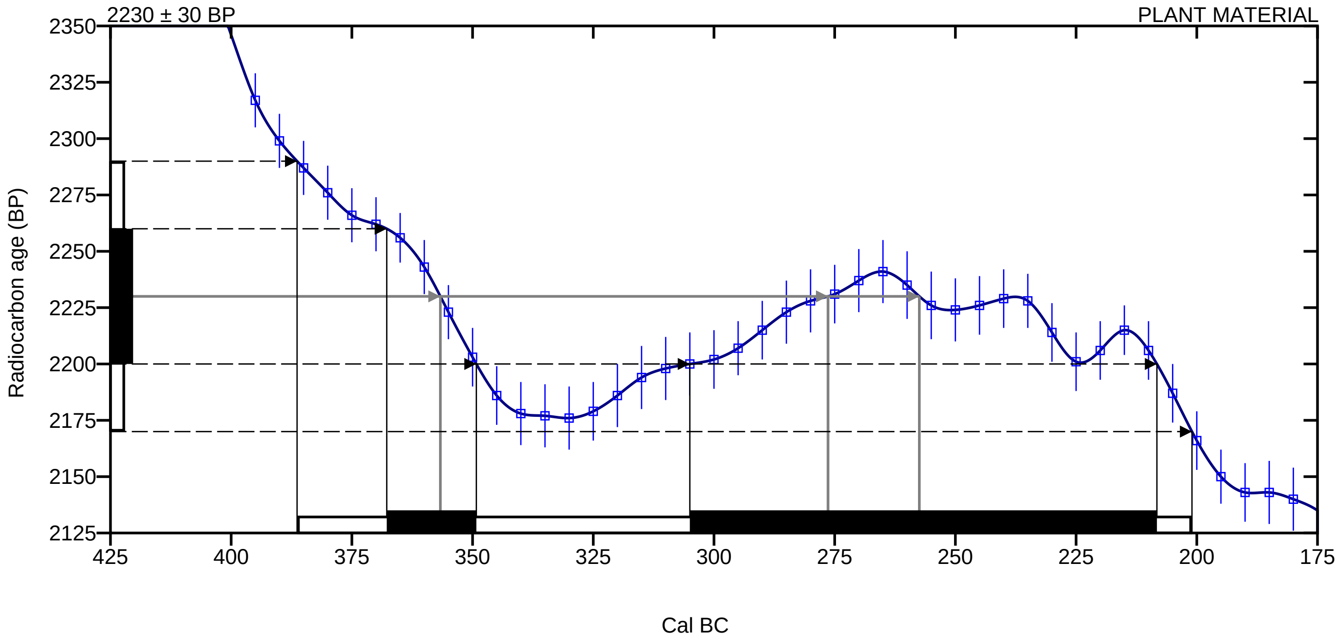

Database used

INTCAL13

References

Mathematics used for calibration scenario

A Simplified Approach to Calibrating C14 Dates, Talma, A. S., Vogel, J. C., 1993, Radiocarbon 35(2):317-322

References to INTCAL13 database

Reimer PJ et al. IntCal13 and Marine13 radiocarbon age calibration curves 0– 50,000 years cal BP. Radiocarbon 55(4):1869– 1887., 2013.

CALIBRATION OF RADIOCARBON AGE TO CALENDAR YEARS

(Variables: C13/C12 = -26.8 o/oo : lab. mult = 1)

**Laboratory number**	**Beta-431187 : FB 45-10**
**Conventional radiocarbon age**	**2240 ± 30 BP**
**Calibrated Result (95% Probability)**	**Cal BC 390 to 340 (Cal BP 2340 to 2290)**<br>**Cal BC 325 to 205 (Cal BP 2275 to 2155)**
Intercept of radiocarbon age with calibration curve	Cal BC 360 (Cal BP 2310)<br>Cal BC 265 (Cal BP 2215)
Calibrated Result (68% Probability)	Cal BC 375 to 350 (Cal BP 2325 to 2300)<br>Cal BC 295 to 230 (Cal BP 2245 to 2180)<br>Cal BC 220 to 210 (Cal BP 2170 to 2160)

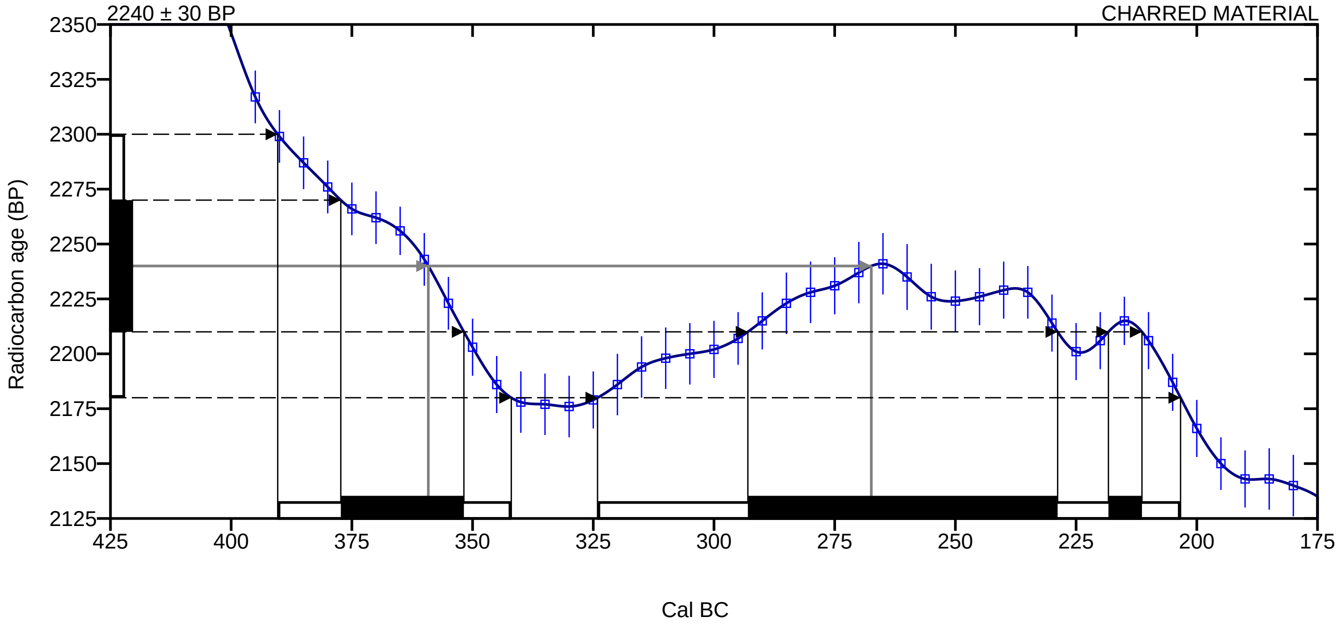

Database used

INTCAL13

References

Mathematics used for calibration scenario

A Simplified Approach to Calibrating C14 Dates, Talma, A. S., Vogel, J. C., 1993, Radiocarbon 35(2):317-322

References to INTCAL13 database

Reimer PJ et al. IntCal13 and Marine13 radiocarbon age calibration curves 0– 50,000 years cal BP. Radiocarbon 55(4):1869– 1887., 2013.

CALIBRATION OF RADIOCARBON AGE TO CALENDAR YEARS

(Variables: C13/C12 = -26 o/oo : lab. mult = 1)

Laboratory number **Beta-431188 : FB 44-16**

Conventional radiocarbon age **2250 ± 30 BP**

Calibrated Result (95% Probability) **Cal BC 395 to 345 (Cal BP 2345 to 2295)**
Cal BC 320 to 205 (Cal BP 2270 to 2155)

Intercept of radiocarbon age with calibration curve Cal BC 360 (Cal BP 2310)

Calibrated Result (68% Probability) Cal BC 380 to 355 (Cal BP 2330 to 2305)
Cal BC 285 to 230 (Cal BP 2235 to 2180)

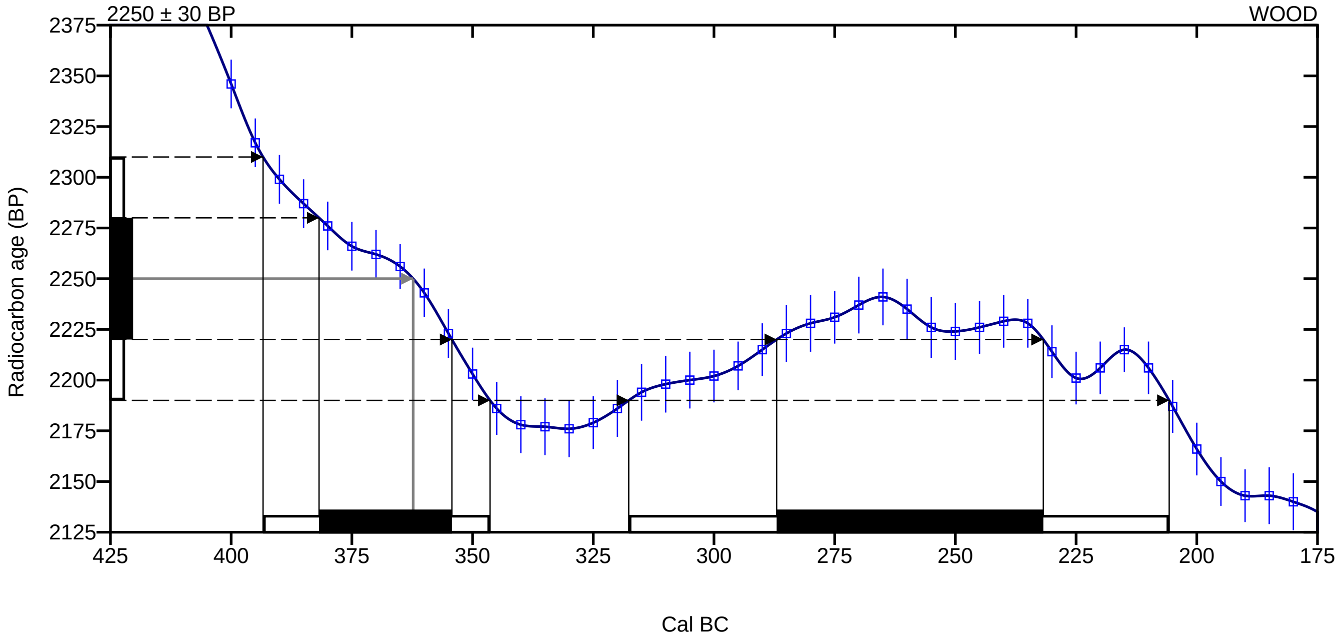

Database used
INTCAL13

References
Mathematics used for calibration scenario
A Simplified Approach to Calibrating C14 Dates, Talma, A. S., Vogel, J. C., 1993, Radiocarbon 35(2):317-322
References to INTCAL13 database
Reimer PJ et al. IntCal13 and Marine13 radiocarbon age calibration curves 0– 50,000 years cal BP. Radiocarbon 55(4):1869– 1887., 2013.

Center for Isotopic Research on Cultural and Environmental heritage

INNOVA

Centro per lo Sviluppo ed il Trasferimento dell'Innovazione nel Settore dei Beni Culturali e Ambientali – INNOVA Scarl

Dipartimento di Matematica e Fisica

CIRCE
Viale Carlo III n. 153
81020 San Nicola La Strada
Tel. +39 0823 274814

Order number/ *Numero d'ordine*	**D**
Customer / *Committente*	**SADORI**
Report Date/ *Data del report*	**22/02/2017**

User code: *Codice utente*	**FB38_15**
SAMPLE: *CAMPIONE:*	**D-DSH7887_PO**
Radiocarbon age: *Età Radiocarbonica*	**5716±42 a BP**
$\delta^{13}C$:	**-18±1‰**

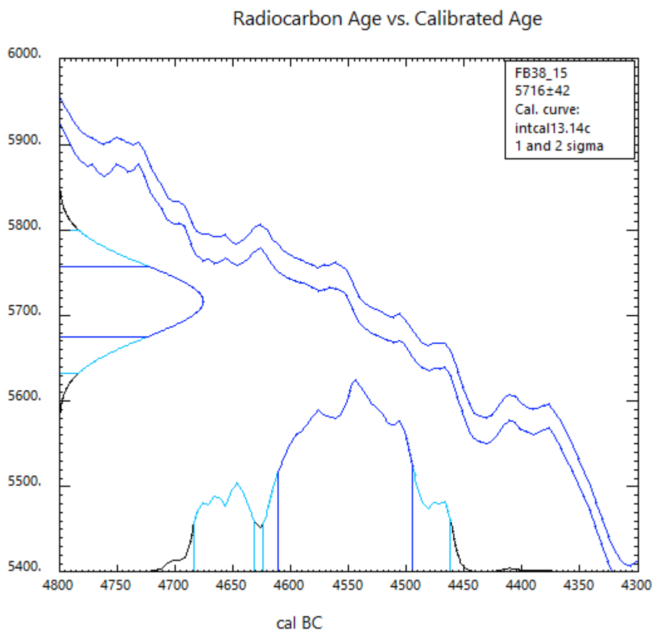

Calibration data set: intcal13.14c # Reimer et al. 2013

confidence level *livello di confidenza*	cal AD/BC age ranges *intervalli di età calibrata*	relative probability *probabilità relativa*
68.3 (1 sigma)	cal **BC 4610 - BC 4494**	100.0%
	-	
95.4 (2 sigma)	cal **BC 4683 - BC 4631**	15.0%
	**BC 4624 - BC 4461**	85.0%
	-	

User code:<br>*Codice utente*	**FB40_13**
SAMPLE:<br>*CAMPIONE:*	**DSH7836_PO**
Radiocarbon age:<br>*Età Radiocarbonica*	**4879±28 a BP**
$\delta^{13}C$:	**-30±2‰**

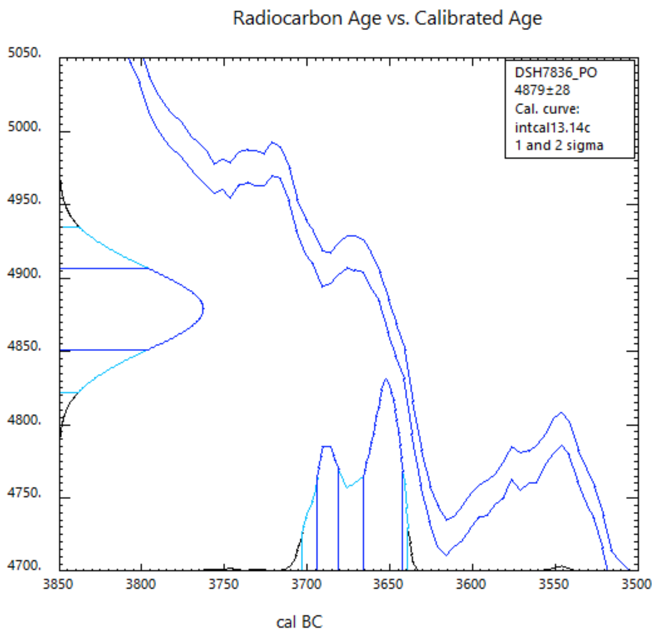

Calibration data set: intcal13.14c # Reimer et al. 2013

confidence level<br>*livello di confidenza*	cal AD/BC age ranges<br>*intervalli di età calibrata*	relative probability<br>*probabilità relativa*
68.3 (1 sigma)	cal **BC 3694 - BC 3680**	29.6%
	**BC 3665 - BC 3642**	70.4%
	-	
95.4 (2 sigma)	cal **BC 3703 - BC 3639**	100.0%
	-	

(Variables: C13/C12 = -25.2 o/oo : lab. mult = 1)

Laboratory number **Beta-422662 : FB40-10**

Conventional radiocarbon age **2830 ± 30 BP**

Calibrated Result (95% Probability) **Cal BC 1050 to 910 (Cal BP 3000 to 2860)**

Intercept of radiocarbon age with calibration curve Cal BC 1000 (Cal BP 2950)

Calibrated Result (68% Probability) Cal BC 1015 to 970 (Cal BP 2965 to 2920)
Cal BC 960 to 930 (Cal BP 2910 to 2880)

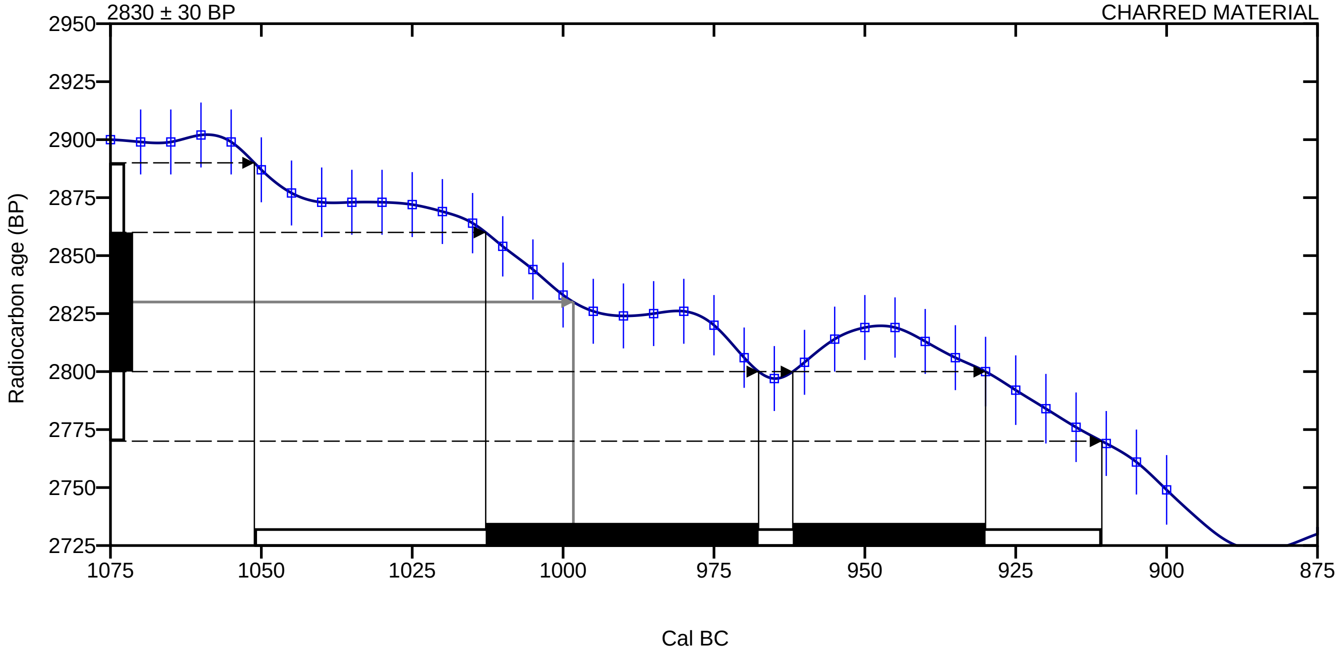

Database used
INTCAL13

References
Mathematics used for calibration scenario
A Simplified Approach to Calibrating C14 Dates, Talma, A. S., Vogel, J. C., 1993, Radiocarbon 35(2):317-322
References to INTCAL13 database
Reimer PJ et al. IntCal13 and Marine13 radiocarbon age calibration curves 0– 50,000 years cal BP. Radiocarbon 55(4):1869– 1887., 2013.

Beta Analytic Radiocarbon Dating Laboratory
4985 S.W. 74th Court, Miami, Florida 33155 • Tel: (305)667-5167 • Fax: (305)663-0964 • Email: beta@radiocarbon.com

CALIBRATION OF RADIOCARBON AGE TO CALENDAR YEARS

(Variables: C13/C12 = -27.2 o/oo : lab. mult = 1)

**Laboratory number**	**Beta-422664 : FB47-9**
**Conventional radiocarbon age**	**3280 ± 30 BP**
**Calibrated Result (95% Probability)**	**Cal BC 1625 to 1500 (Cal BP 3575 to 3450)**
Intercept of radiocarbon age with calibration curve	Cal BC 1595 (Cal BP 3545)<br>Cal BC 1585 (Cal BP 3535)<br>Cal BC 1530 (Cal BP 3480)
Calibrated Result (68% Probability)	Cal BC 1610 to 1505 (Cal BP 3560 to 3455)

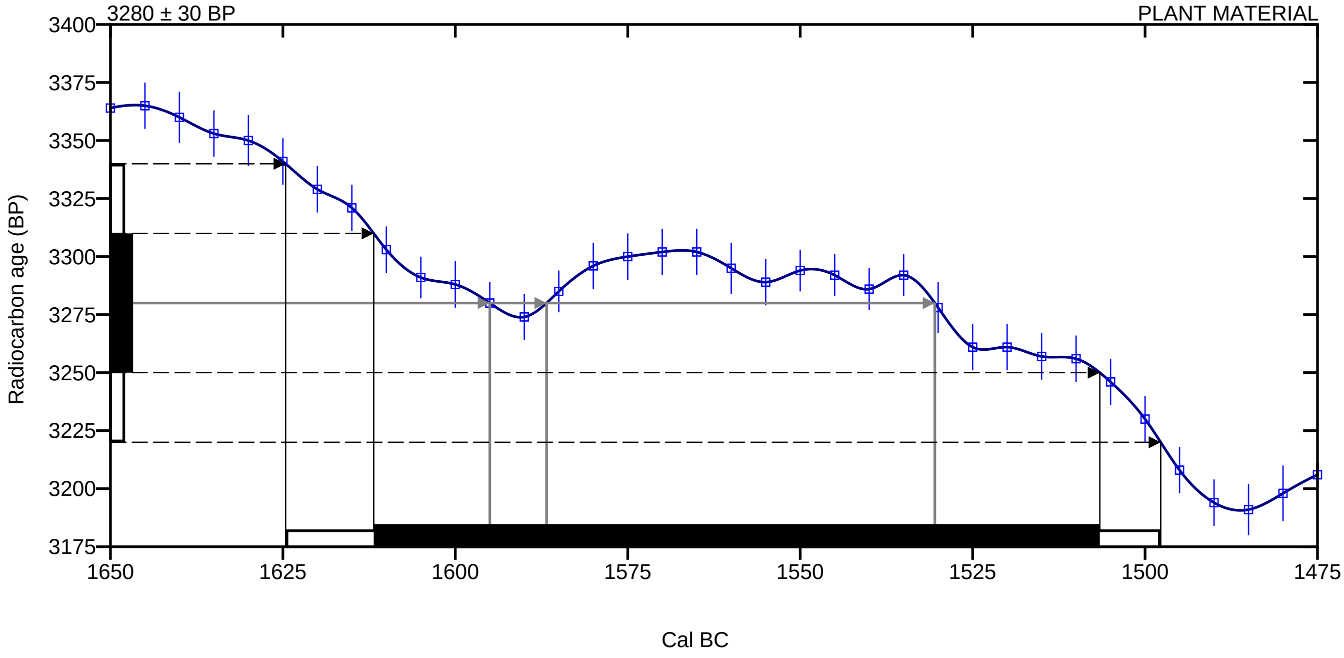

Database used
INTCAL13

References
Mathematics used for calibration scenario

A Simplified Approach to Calibrating C14 Dates, Talma, A. S., Vogel, J. C., 1993, Radiocarbon 35(2):317-322

References to INTCAL13 database

Reimer PJ et al. IntCal13 and Marine13 radiocarbon age calibration curves 0– 50,000 years cal BP. Radiocarbon 55(4):1869– 1887., 2013.

CALIBRATION OF RADIOCARBON AGE TO CALENDAR YEARS

(Variables: C13/C12 = -28 o/oo : lab. mult = 1)

Laboratory number **Beta-422665 : FB47-12**

Conventional radiocarbon age **2580 ± 30 BP**

Calibrated Result (95% Probability) **Cal BC 805 to 765 (Cal BP 2755 to 2715)**

Intercept of radiocarbon age with calibration curve Cal BC 790 (Cal BP 2740)

Calibrated Result (68% Probability) Cal BC 800 to 780 (Cal BP 2750 to 2730)

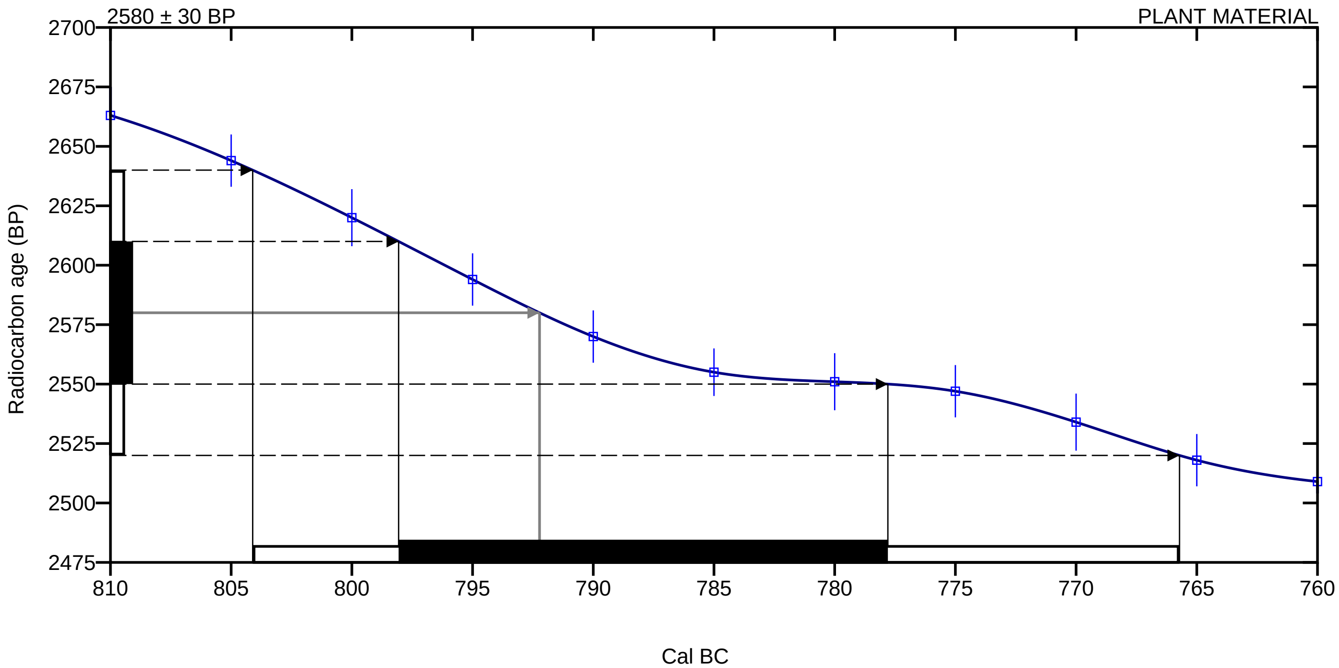

Database used

INTCAL13

References

Mathematics used for calibration scenario

A Simplified Approach to Calibrating C14 Dates, Talma, A. S., Vogel, J. C., 1993, Radiocarbon 35(2):317-322

References to INTCAL13 database

Reimer PJ et al. IntCal13 and Marine13 radiocarbon age calibration curves 0– 50,000 years cal BP. Radiocarbon 55(4):1869– 1887., 2013.

CALIBRATION OF RADIOCARBON AGE TO CALENDAR YEARS

(Variables: C13/C12 = -28 o/oo : lab. mult = 1)

**Laboratory number**	**Beta-422666 : FB47-17**
**Conventional radiocarbon age**	**3500 ± 30 BP**
**Calibrated Result (95% Probability)**	**Cal BC 1900 to 1745 (Cal BP 3850 to 3695)**
Intercept of radiocarbon age with calibration curve	Cal BC 1875 (Cal BP 3825)<br>Cal BC 1840 (Cal BP 3790)<br>Cal BC 1820 (Cal BP 3770)<br>Cal BC 1795 (Cal BP 3745)<br>Cal BC 1780 (Cal BP 3730)
Calibrated Result (68% Probability)	Cal BC 1885 to 1765 (Cal BP 3835 to 3715)

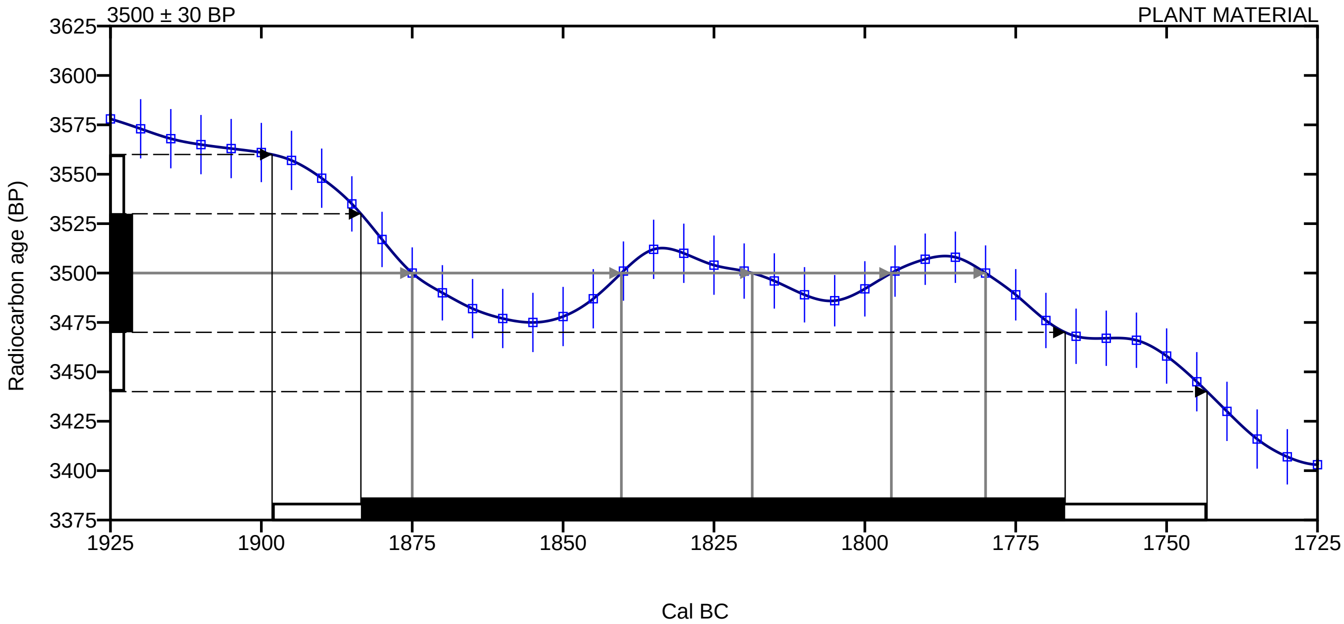

Database used

INTCAL13

References

Mathematics used for calibration scenario

A Simplified Approach to Calibrating C14 Dates, Talma, A. S., Vogel, J. C., 1993, Radiocarbon 35(2):317-322

References to INTCAL13 database

Reimer PJ et al. IntCal13 and Marine13 radiocarbon age calibration curves 0– 50,000 years cal BP. Radiocarbon 55(4):1869– 1887., 2013.

Beta Analytic Radiocarbon Dating Laboratory
4985 S.W. 74th Court, Miami, Florida 33155 • Tel: (305)667-5167 • Fax: (305)663-0964 • Email: beta@radiocarbon.com

CALIBRATION OF RADIOCARBON AGE TO CALENDAR YEARS

(Variables: C13/C12 = -28.7 o/oo : lab. mult = 1)

Laboratory number **Beta-422668 : FB48-20**

Conventional radiocarbon age **7250 ± 30 BP**

Calibrated Result (95% Probability) **Cal BC 6215 to 6050 (Cal BP 8165 to 8000)**

Intercept of radiocarbon age with calibration curve Cal BC 6075 (Cal BP 8025)

Calibrated Result (68% Probability) Cal BC 6200 to 6195 (Cal BP 8150 to 8145)
Cal BC 6100 to 6065 (Cal BP 8050 to 8015)

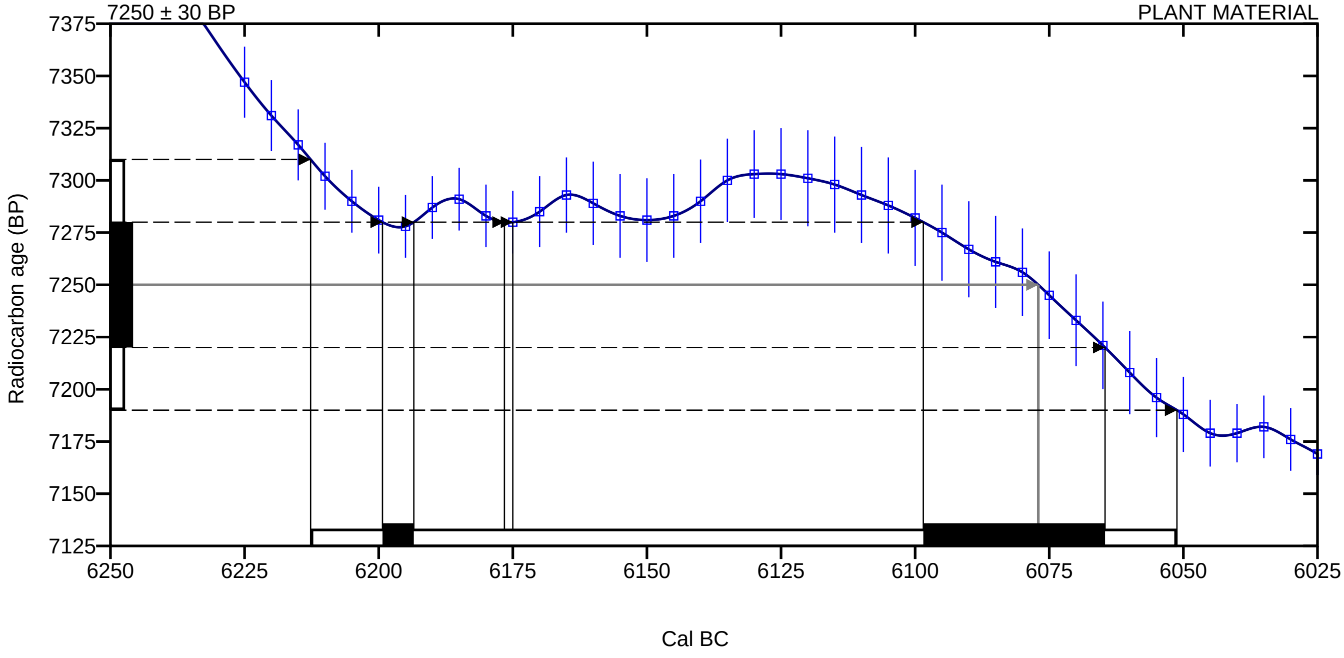

Database used

INTCAL13

References

Mathematics used for calibration scenario

A Simplified Approach to Calibrating C14 Dates, Talma, A. S., Vogel, J. C., 1993, Radiocarbon 35(2):317-322

References to INTCAL13 database

Reimer PJ et al. IntCal13 and Marine13 radiocarbon age calibration curves 0–50,000 years cal BP. Radiocarbon 55(4):1869–1887., 2013.

CALIBRATION OF RADIOCARBON AGE TO CALENDAR YEARS

(Variables: C13/C12 = -28.5 o/oo : lab. mult = 1)

Laboratory number **Beta-422669 : FB48-21**

Conventional radiocarbon age **7350 ± 30 BP**

Calibrated Result (95% Probability)
Cal BC 6250 to 6205 (Cal BP 8200 to 8155)
Cal BC 6190 to 6185 (Cal BP 8140 to 8135)
Cal BC 6165 to 6160 (Cal BP 8115 to 8110)
Cal BC 6140 to 6105 (Cal BP 8090 to 8055)

Intercept of radiocarbon age with calibration curve Cal BC 6225 (Cal BP 8175)

Calibrated Result (68% Probability) Cal BC 6235 to 6215 (Cal BP 8185 to 8165)

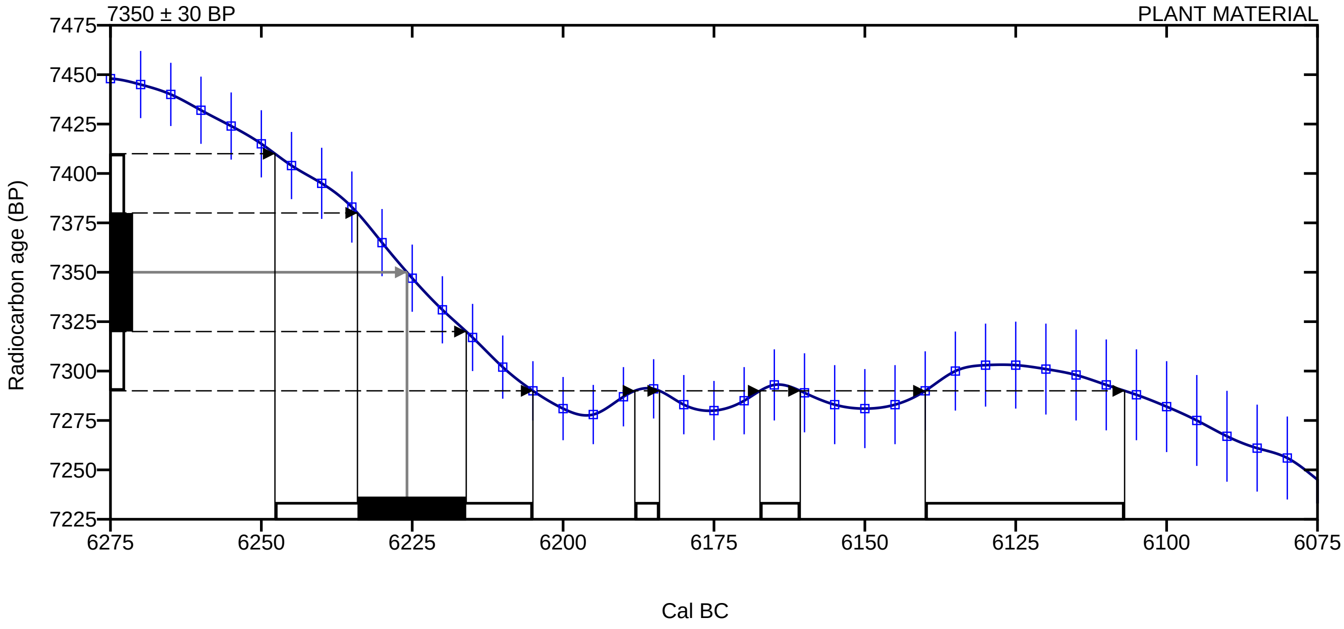

Database used
INTCAL13

References

Mathematics used for calibration scenario
A Simplified Approach to Calibrating C14 Dates, Talma, A. S., Vogel, J. C., 1993, Radiocarbon 35(2):317-322
References to INTCAL13 database
Reimer PJ et al. IntCal13 and Marine13 radiocarbon age calibration curves 0– 50,000 years cal BP. Radiocarbon 55(4):1869– 1887., 2013.

Beta Analytic Radiocarbon Dating Laboratory
4985 S.W. 74th Court, Miami, Florida 33155 • Tel: (305)667-5167 • Fax: (305)663-0964 • Email: beta@radiocarbon.com

CALIBRATION OF RADIOCARBON AGE TO CALENDAR YEARS

(Variables: C13/C12 = -27.6 o/oo : lab. mult = 1)

Laboratory number **Beta-422670 : FB48-37**

Conventional radiocarbon age **9210 ± 30 BP**

Calibrated Result (95% Probability) **Cal BC 8545 to 8505 (Cal BP 10495 to 10455)**
Cal BC 8490 to 8305 (Cal BP 10440 to 10255)

Intercept of radiocarbon age with calibration curve
Cal BC 8435 (Cal BP 10385)
Cal BC 8365 (Cal BP 10315)
Cal BC 8350 (Cal BP 10300)

Calibrated Result (68% Probability) Cal BC 8465 to 8330 (Cal BP 10415 to 10280)

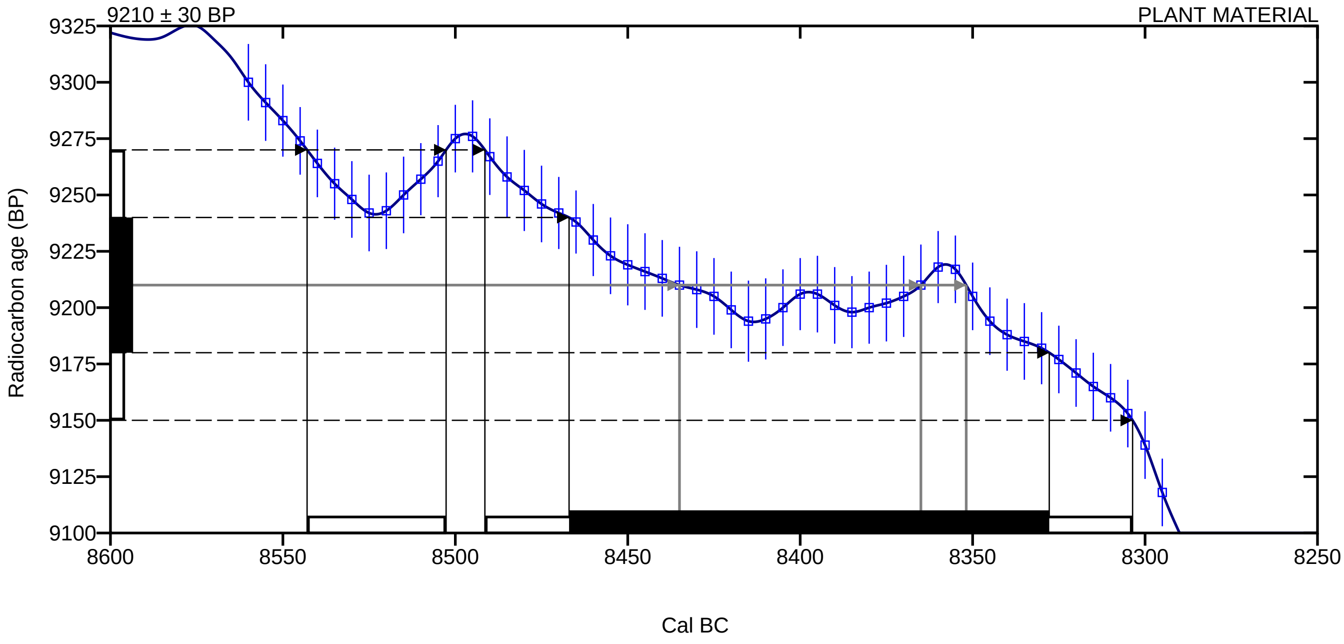

Database used

INTCAL13

References

Mathematics used for calibration scenario
A Simplified Approach to Calibrating C14 Dates, Talma, A. S., Vogel, J. C., 1993, Radiocarbon 35(2):317-322
References to INTCAL13 database
Reimer PJ et al. IntCal13 and Marine13 radiocarbon age calibration curves 0– 50,000 years cal BP. Radiocarbon 55(4):1869– 1887., 2013.

CALIBRATION OF RADIOCARBON AGE TO CALENDAR YEARS

(Variables: C13/C12 = -24.4 o/oo : lab. mult = 1)

Laboratory number **Beta-422672 : S3-C2-21.0**

Conventional radiocarbon age **5570 ± 30 BP**

Calibrated Result (95% Probability) **Cal BC 4455 to 4345 (Cal BP 6405 to 6295)**

Intercept of radiocarbon age with calibration curve
Cal BC 4440 (Cal BP 6390)
Cal BC 4425 (Cal BP 6375)
Cal BC 4370 (Cal BP 6320)

Calibrated Result (68% Probability) Cal BC 4450 to 4355 (Cal BP 6400 to 6305)

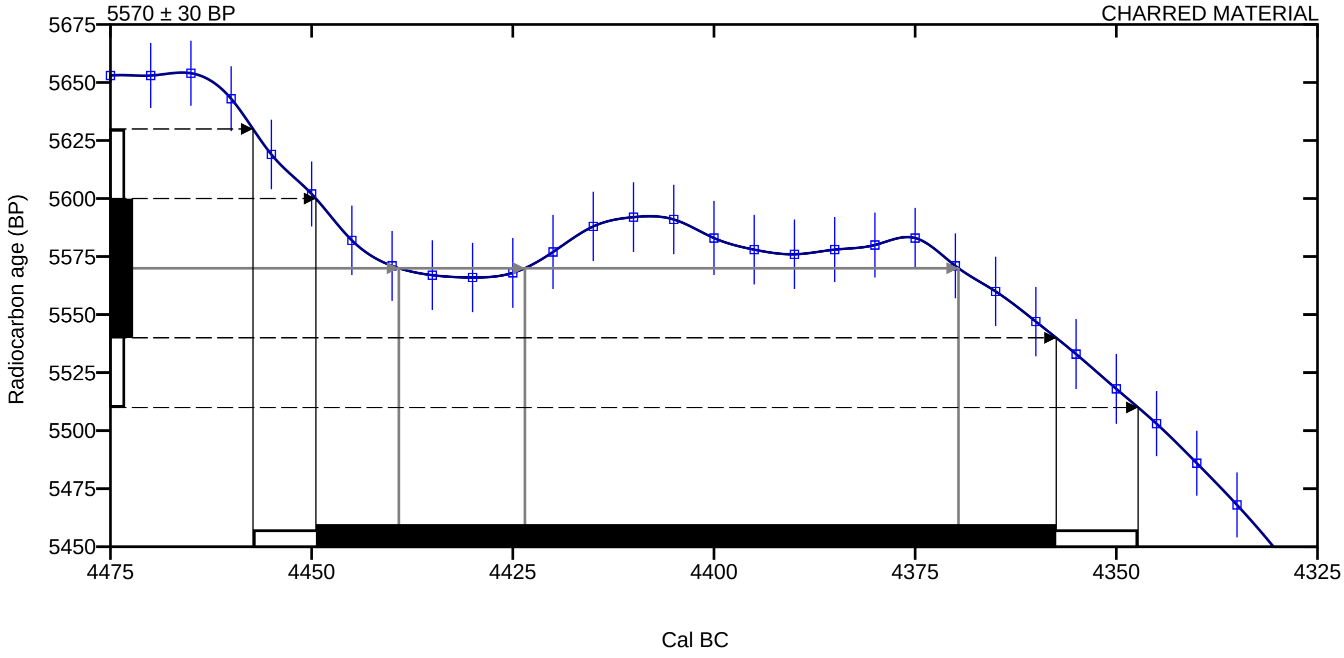

Database used
INTCAL13

References
Mathematics used for calibration scenario
A Simplified Approach to Calibrating C14 Dates, Talma, A. S., Vogel, J. C., 1993, Radiocarbon 35(2):317-322
References to INTCAL13 database
Reimer PJ et al. IntCal13 and Marine13 radiocarbon age calibration curves 0–50,000 years cal BP. Radiocarbon 55(4):1869–1887., 2013.

(Variables: C13/C12 = -26.9 o/oo : lab. mult = 1)

Laboratory number **Beta-422673 : S3-C5-22.8**

Conventional radiocarbon age **4580 ± 30 BP**

Calibrated Result (95% Probability)
Cal BC 3490 to 3470 (Cal BP 5440 to 5420)
Cal BC 3370 to 3335 (Cal BP 5320 to 5285)
Cal BC 3210 to 3190 (Cal BP 5160 to 5140)
Cal BC 3150 to 3140 (Cal BP 5100 to 5090)

Intercept of radiocarbon age with calibration curve Cal BC 3360 (Cal BP 5310)

Calibrated Result (68% Probability) Cal BC 3365 to 3350 (Cal BP 5315 to 5300)

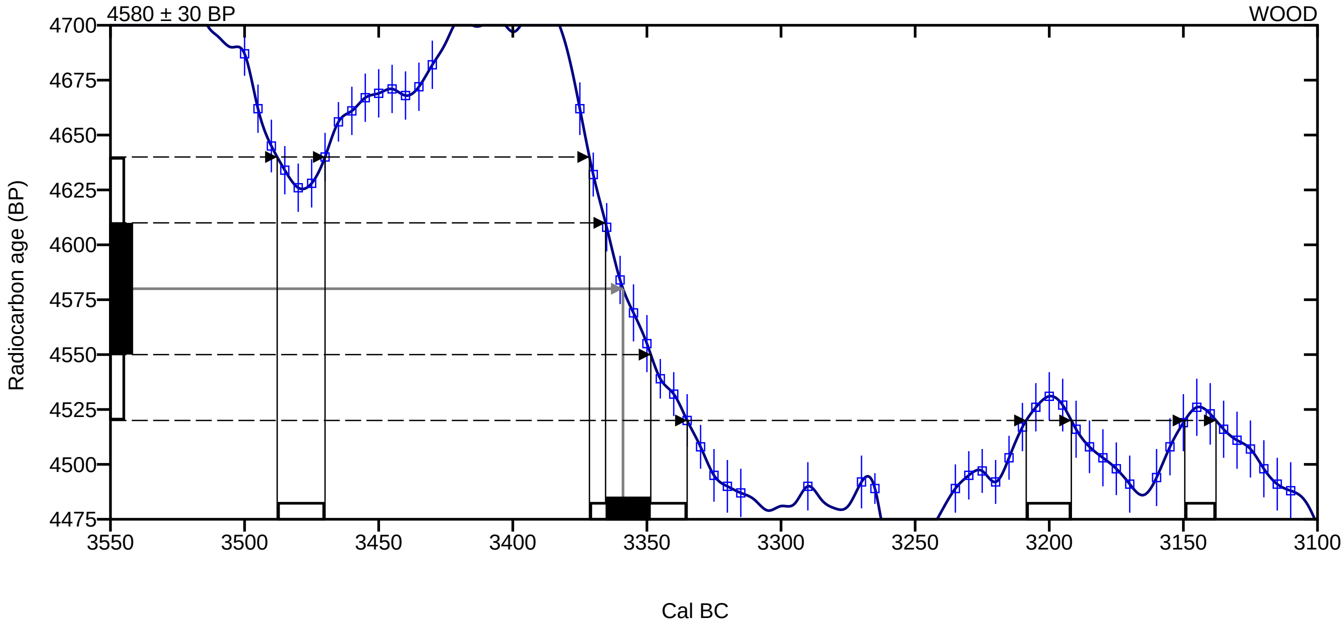

Database used
INTCAL13

References
Mathematics used for calibration scenario
A Simplified Approach to Calibrating C14 Dates, Talma, A. S., Vogel, J. C., 1993, Radiocarbon 35(2):317-322
References to INTCAL13 database
Reimer PJ et al. IntCal13 and Marine13 radiocarbon age calibration curves 0– 50,000 years cal BP. Radiocarbon 55(4):1869– 1887., 2013.

CALIBRATION OF RADIOCARBON AGE TO CALENDAR YEARS

(Variables: C13/C12 = -25.6 o/oo : lab. mult = 1)

Laboratory number **Beta-425692 : FB48-48.5**

Conventional radiocarbon age **11720 ± 40 BP**

Calibrated Result (95% Probability) **Cal BC 11635 to 11510 (Cal BP 13585 to 13460)**

Intercept of radiocarbon age with calibration curve Cal BC 11600 (Cal BP 13550)

Calibrated Result (68% Probability) Cal BC 11615 to 11530 (Cal BP 13565 to 13480)

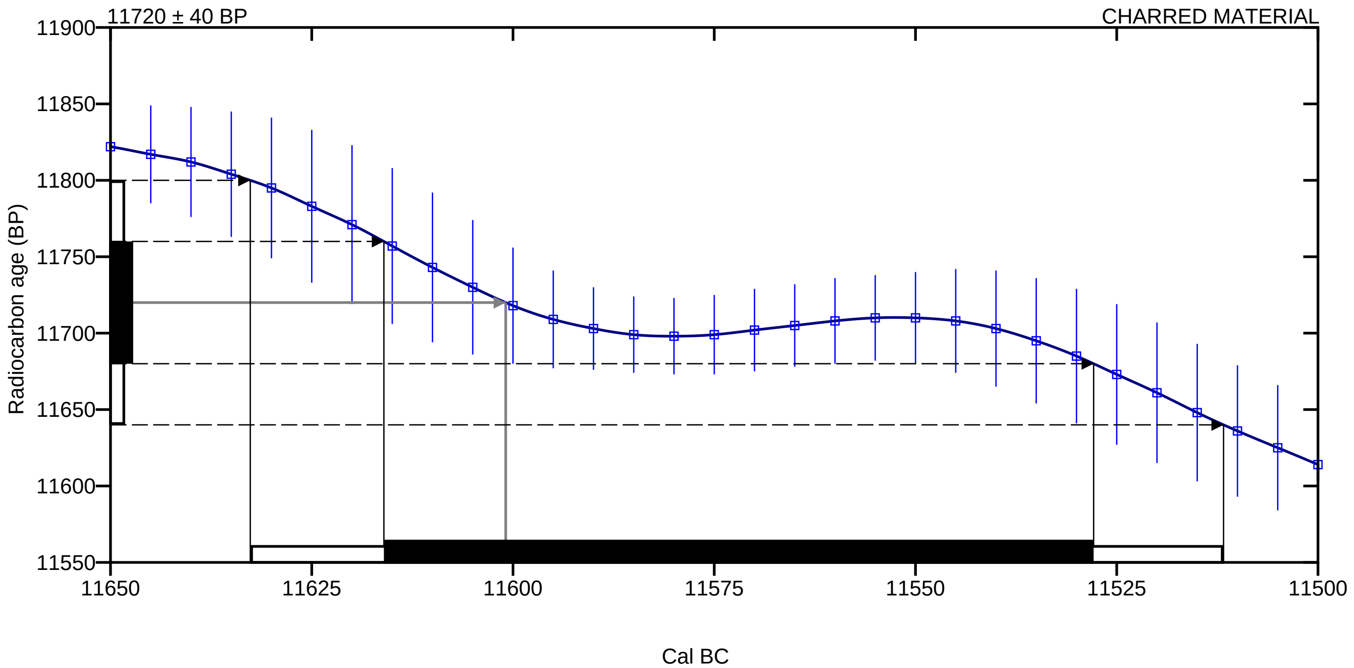

Database used

INTCAL13

References

Mathematics used for calibration scenario

A Simplified Approach to Calibrating C14 Dates, Talma, A. S., Vogel, J. C., 1993, Radiocarbon 35(2):317-322

References to INTCAL13 database

Reimer PJ et al. IntCal13 and Marine13 radiocarbon age calibration curves 0– 50,000 years cal BP. Radiocarbon 55(4):1869– 1887., 2013.

CALIBRATION OF RADIOCARBON AGE TO CALENDAR YEARS

(Variables: C13/C12 = -26.7 o/oo : lab. mult = 1)

**Laboratory number**	**Beta-424137 : FB38-16B**
**Conventional radiocarbon age**	**6170 ± 30 BP**
**Calibrated Result (95% Probability)**	**Cal BC 5215 to 5025 (Cal BP 7165 to 6975)**
Intercept of radiocarbon age with calibration curve	Cal BC 5205 (Cal BP 7155)<br>Cal BC 5170 (Cal BP 7120)<br>Cal BC 5075 (Cal BP 7025)
Calibrated Result (68% Probability)	Cal BC 5210 to 5055 (Cal BP 7160 to 7005)

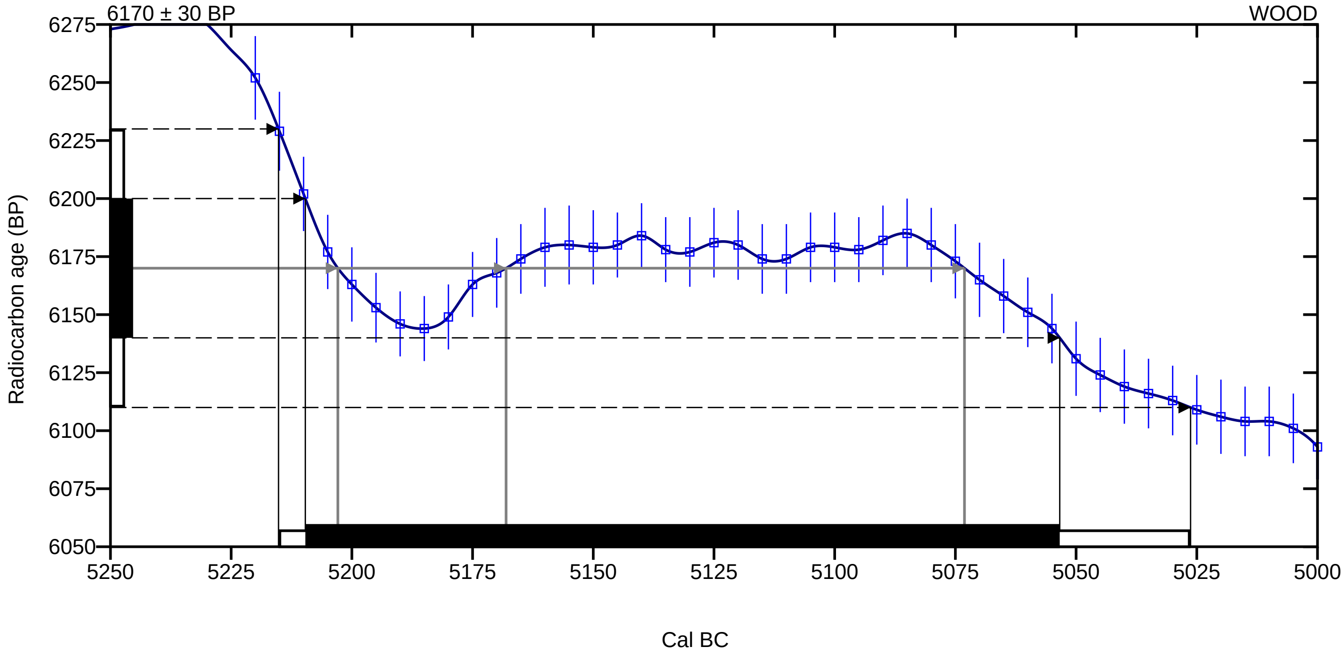

Database used

INTCAL13

References

Mathematics used for calibration scenario

A Simplified Approach to Calibrating C14 Dates, Talma, A. S., Vogel, J. C., 1993, Radiocarbon 35(2):317-322

References to INTCAL13 database

Reimer PJ et al. IntCal13 and Marine13 radiocarbon age calibration curves 0–50,000 years cal BP. Radiocarbon 55(4):1869–1887., 2013.

Beta Analytic Radiocarbon Dating Laboratory

4985 S.W. 74th Court, Miami, Florida 33155 • Tel: (305)667-5167 • Fax: (305)663-0964 • Email: beta@radiocarbon.com

(Variables: C13/C12 = -19.3 o/oo : lab. mult = 1)

Laboratory number **Beta-424138 : FB39-15**

Conventional radiocarbon age **2430 ± 30 BP**

Calibrated Result (95% Probability)
Cal BC 750 to 685 (Cal BP 2700 to 2635)
Cal BC 665 to 640 (Cal BP 2615 to 2590)
Cal BC 590 to 405 (Cal BP 2540 to 2355)

Intercept of radiocarbon age with calibration curve: Cal BC 510 (Cal BP 2460)

Calibrated Result (68% Probability)
Cal BC 730 to 690 (Cal BP 2680 to 2640)
Cal BC 660 to 650 (Cal BP 2610 to 2600)
Cal BC 540 to 410 (Cal BP 2490 to 2360)

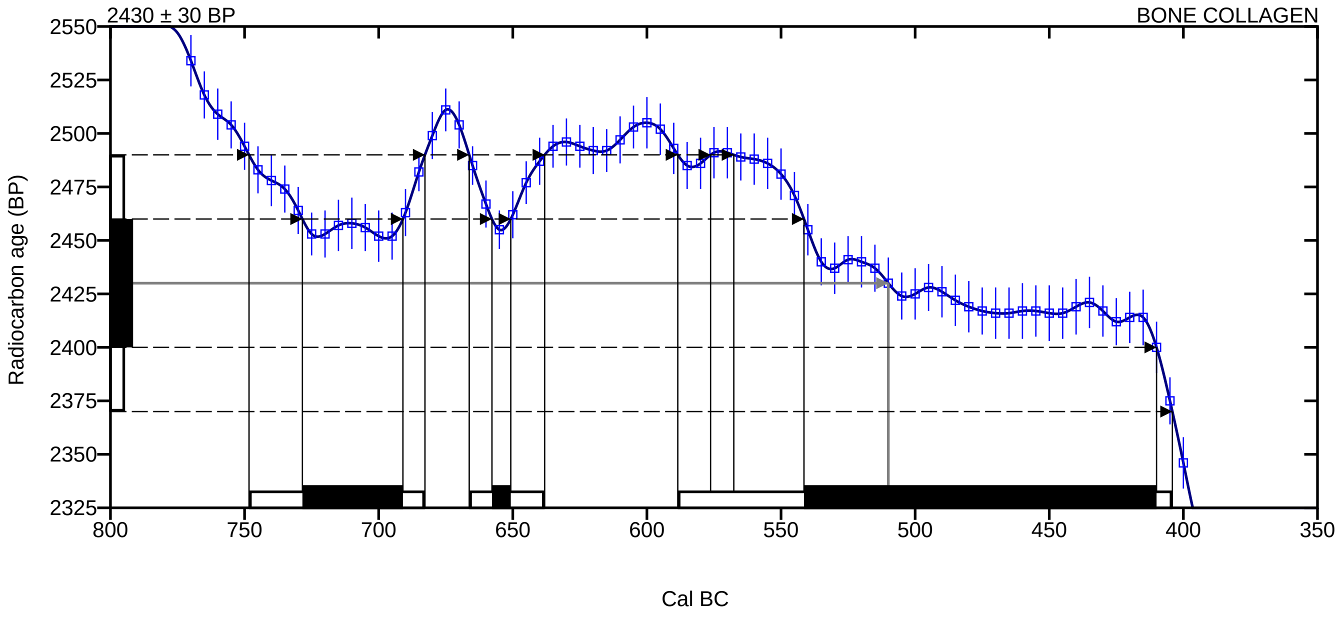

Database used

INTCAL13

References

Mathematics used for calibration scenario

A Simplified Approach to Calibrating C14 Dates, Talma, A. S., Vogel, J. C., 1993, Radiocarbon 35(2):317-322

References to INTCAL13 database

Reimer PJ et al. IntCal13 and Marine13 radiocarbon age calibration curves 0– 50,000 years cal BP. Radiocarbon 55(4):1869– 1887., 2013.

(Variables: C13/C12 = -26.5 o/oo : lab. mult = 1)

**Laboratory number**	**Beta-424139 : FB43-15**
**Conventional radiocarbon age**	**2540 ± 30 BP**
**Calibrated Result (95% Probability)**	**Cal BC 795 to 745 (Cal BP 2745 to 2695)**<br>**Cal BC 685 to 665 (Cal BP 2635 to 2615)**<br>**Cal BC 645 to 550 (Cal BP 2595 to 2500)**
Intercept of radiocarbon age with calibration curve	Cal BC 770 (Cal BP 2720)
Calibrated Result (68% Probability)	Cal BC 790 to 760 (Cal BP 2740 to 2710)

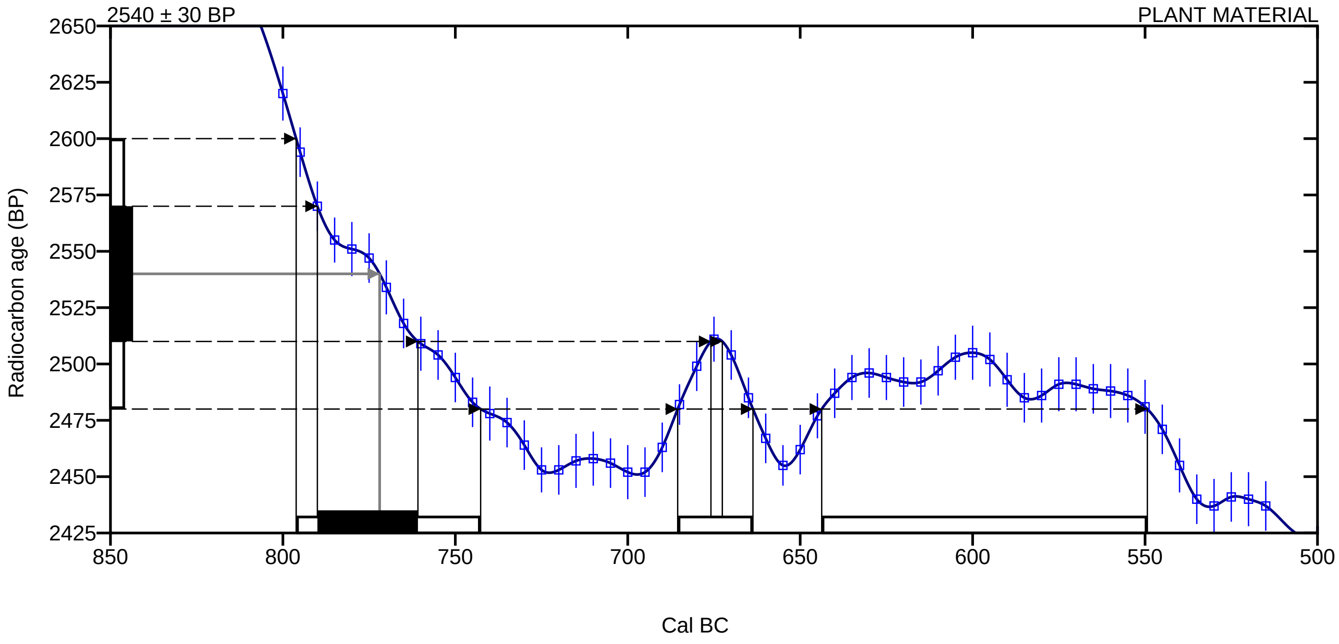

Database used

INTCAL13

References

Mathematics used for calibration scenario

A Simplified Approach to Calibrating C14 Dates, Talma, A. S., Vogel, J. C., 1993, Radiocarbon 35(2):317-322

References to INTCAL13 database

Reimer PJ et al. IntCal13 and Marine13 radiocarbon age calibration curves 0– 50,000 years cal BP. Radiocarbon 55(4):1869– 1887., 2013.

CALIBRATION OF RADIOCARBON AGE TO CALENDAR YEARS

(Variables: C13/C12 = -27.1 o/oo : lab. mult = 1)

**Laboratory number**	**Beta-424140 : FB48-14.7**
**Conventional radiocarbon age**	**2690 ± 30 BP**
**Calibrated Result (95% Probability)**	**Cal BC 900 to 800 (Cal BP 2850 to 2750)**
Intercept of radiocarbon age with calibration curve	Cal BC 825 (Cal BP 2775)
Calibrated Result (68% Probability)	Cal BC 840 to 810 (Cal BP 2790 to 2760)

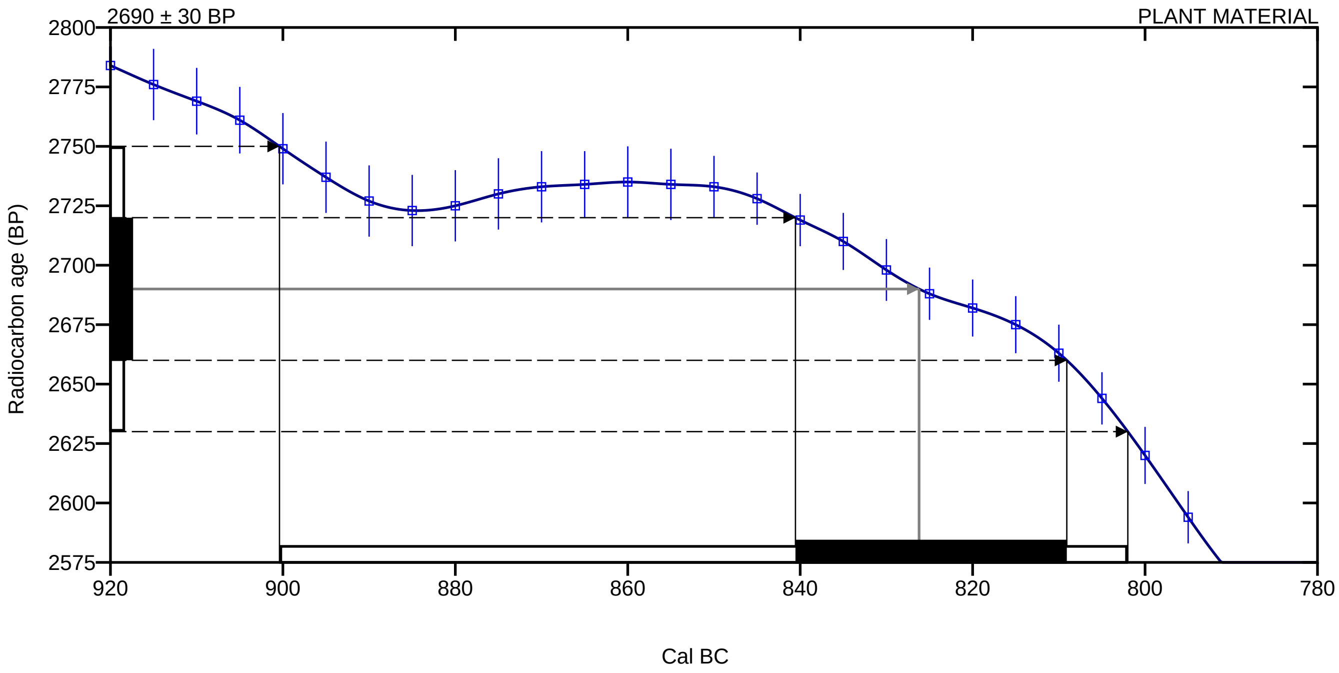

Database used
INTCAL13

References

Mathematics used for calibration scenario

A Simplified Approach to Calibrating C14 Dates, Talma, A. S., Vogel, J. C., 1993, Radiocarbon 35(2):317-322

References to INTCAL13 database

Reimer PJ et al. IntCal13 and Marine13 radiocarbon age calibration curves 0– 50,000 years cal BP. Radiocarbon 55(4):1869– 1887., 2013.

CALIBRATION OF RADIOCARBON AGE TO CALENDAR YEARS

(Variables: C13/C12 = -25.2 o/oo : lab. mult = 1)

Laboratory number **Beta-422662 : FB40-10**

Conventional radiocarbon age **2830 ± 30 BP**

Calibrated Result (95% Probability) **Cal BC 1050 to 910 (Cal BP 3000 to 2860)**

Intercept of radiocarbon age with calibration curve Cal BC 1000 (Cal BP 2950)

Calibrated Result (68% Probability) Cal BC 1015 to 970 (Cal BP 2965 to 2920)
Cal BC 960 to 930 (Cal BP 2910 to 2880)

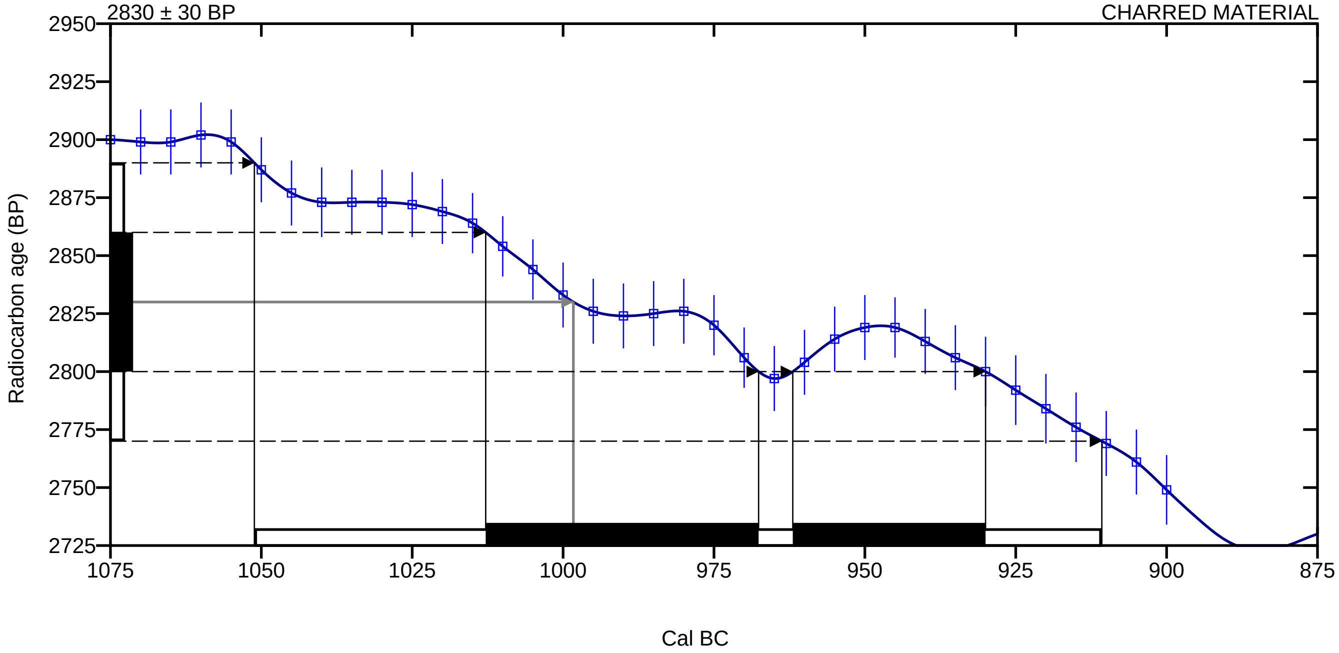

Database used
INTCAL13

References
Mathematics used for calibration scenario
A Simplified Approach to Calibrating C14 Dates, Talma, A. S., Vogel, J. C., 1993, Radiocarbon 35(2):317-322
References to INTCAL13 database
Reimer PJ et al. IntCal13 and Marine13 radiocarbon age calibration curves 0–50,000 years cal BP. Radiocarbon 55(4):1869–1887., 2013.

CALIBRATION OF RADIOCARBON AGE TO CALENDAR YEARS

(Variables: C13/C12 = -27.2 o/oo : lab. mult = 1)

**Laboratory number**	**Beta-422664 : FB47-9**
**Conventional radiocarbon age**	**3280 ± 30 BP**
**Calibrated Result (95% Probability)**	**Cal BC 1625 to 1500 (Cal BP 3575 to 3450)**
Intercept of radiocarbon age with calibration curve	Cal BC 1595 (Cal BP 3545)<br>Cal BC 1585 (Cal BP 3535)<br>Cal BC 1530 (Cal BP 3480)
Calibrated Result (68% Probability)	Cal BC 1610 to 1505 (Cal BP 3560 to 3455)

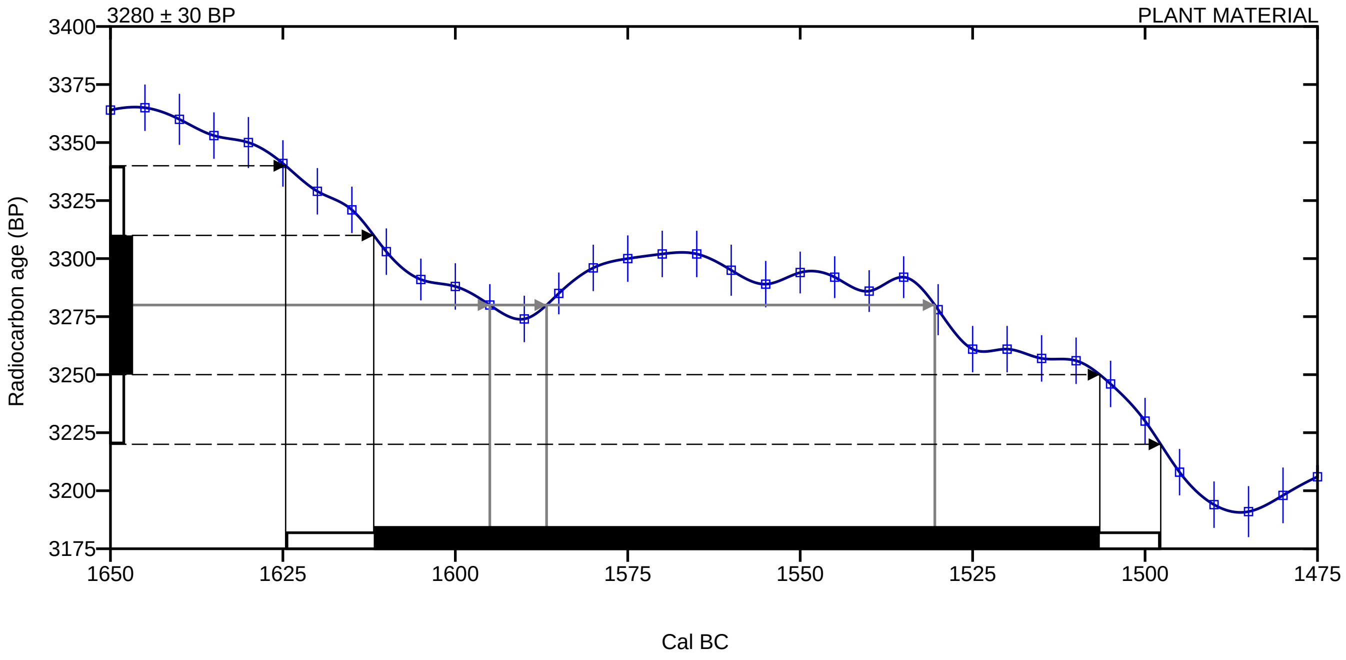

Database used
INTCAL13

References
Mathematics used for calibration scenario

A Simplified Approach to Calibrating C14 Dates, Talma, A. S., Vogel, J. C., 1993, Radiocarbon 35(2):317-322

References to INTCAL13 database

Reimer PJ et al. IntCal13 and Marine13 radiocarbon age calibration curves 0–50,000 years cal BP. Radiocarbon 55(4):1869–1887., 2013.

CALIBRATION OF RADIOCARBON AGE TO CALENDAR YEARS

(Variables: C13/C12 = -28 o/oo : lab. mult = 1)

Laboratory number **Beta-422665 : FB47-12**

Conventional radiocarbon age **2580 ± 30 BP**

Calibrated Result (95% Probability) **Cal BC 805 to 765 (Cal BP 2755 to 2715)**

Intercept of radiocarbon age with calibration curve Cal BC 790 (Cal BP 2740)

Calibrated Result (68% Probability) Cal BC 800 to 780 (Cal BP 2750 to 2730)

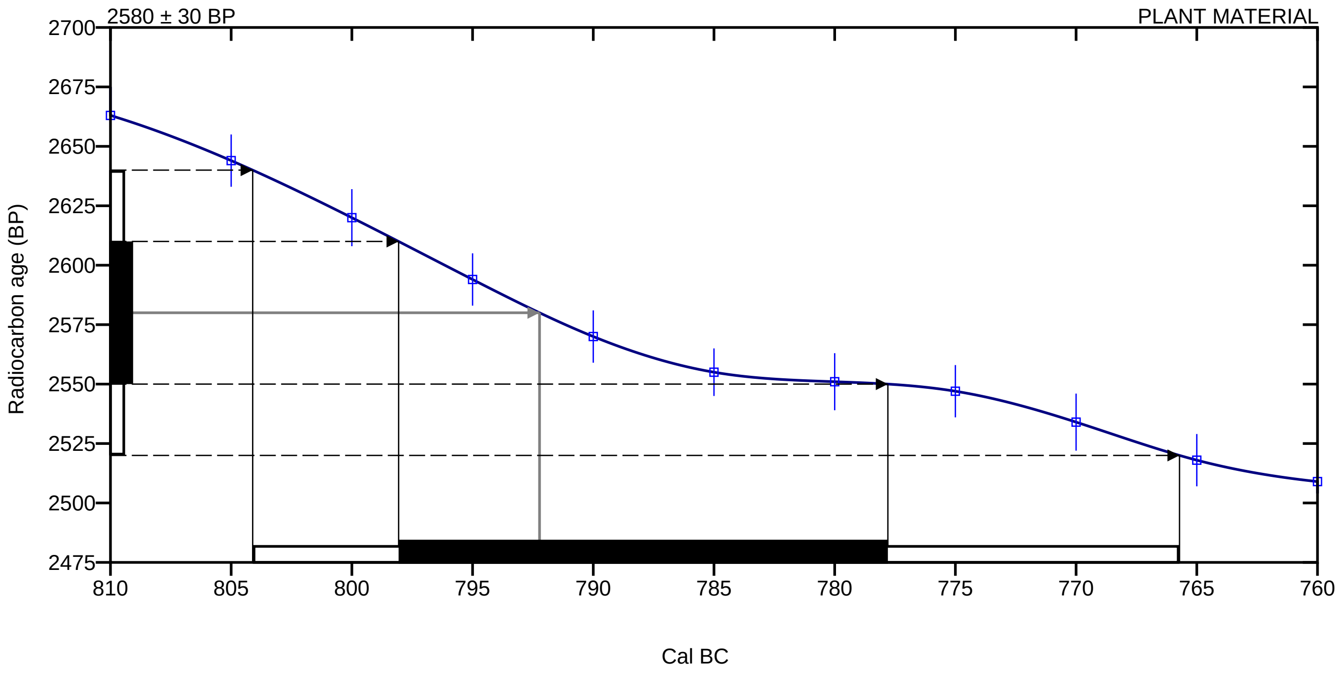

Database used

INTCAL13

References

Mathematics used for calibration scenario

A Simplified Approach to Calibrating C14 Dates, Talma, A. S., Vogel, J. C., 1993, Radiocarbon 35(2):317-322

References to INTCAL13 database

Reimer PJ et al. IntCal13 and Marine13 radiocarbon age calibration curves 0–50,000 years cal BP. Radiocarbon 55(4):1869–1887., 2013.

CALIBRATION OF RADIOCARBON AGE TO CALENDAR YEARS

(Variables: C13/C12 = -28 o/oo : lab. mult = 1)

Laboratory number **Beta-422666 : FB47-17**

Conventional radiocarbon age **3500 ± 30 BP**

Calibrated Result (95% Probability) **Cal BC 1900 to 1745 (Cal BP 3850 to 3695)**

Intercept of radiocarbon age with calibration curve
Cal BC 1875 (Cal BP 3825)
Cal BC 1840 (Cal BP 3790)
Cal BC 1820 (Cal BP 3770)
Cal BC 1795 (Cal BP 3745)
Cal BC 1780 (Cal BP 3730)

Calibrated Result (68% Probability) Cal BC 1885 to 1765 (Cal BP 3835 to 3715)

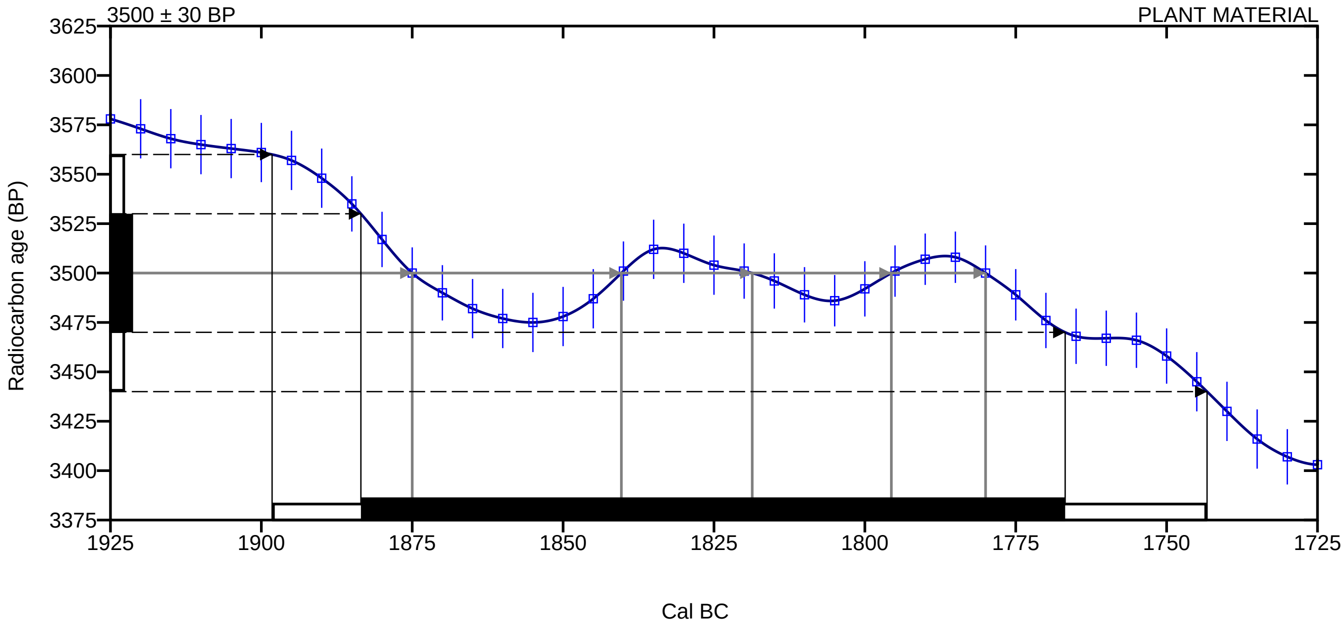

Database used
INTCAL13

References
Mathematics used for calibration scenario

A Simplified Approach to Calibrating C14 Dates, Talma, A. S., Vogel, J. C., 1993, Radiocarbon 35(2):317-322

References to INTCAL13 database

Reimer PJ et al. IntCal13 and Marine13 radiocarbon age calibration curves 0–50,000 years cal BP. Radiocarbon 55(4):1869–1887., 2013.

CALIBRATION OF RADIOCARBON AGE TO CALENDAR YEARS

(Variables: C13/C12 = -28.7 o/oo : lab. mult = 1)

**Laboratory number**	**Beta-422668 : FB48-20**
**Conventional radiocarbon age**	**7250 ± 30 BP**
**Calibrated Result (95% Probability)**	**Cal BC 6215 to 6050 (Cal BP 8165 to 8000)**
Intercept of radiocarbon age with calibration curve	Cal BC 6075 (Cal BP 8025)
Calibrated Result (68% Probability)	Cal BC 6200 to 6195 (Cal BP 8150 to 8145)<br>Cal BC 6100 to 6065 (Cal BP 8050 to 8015)

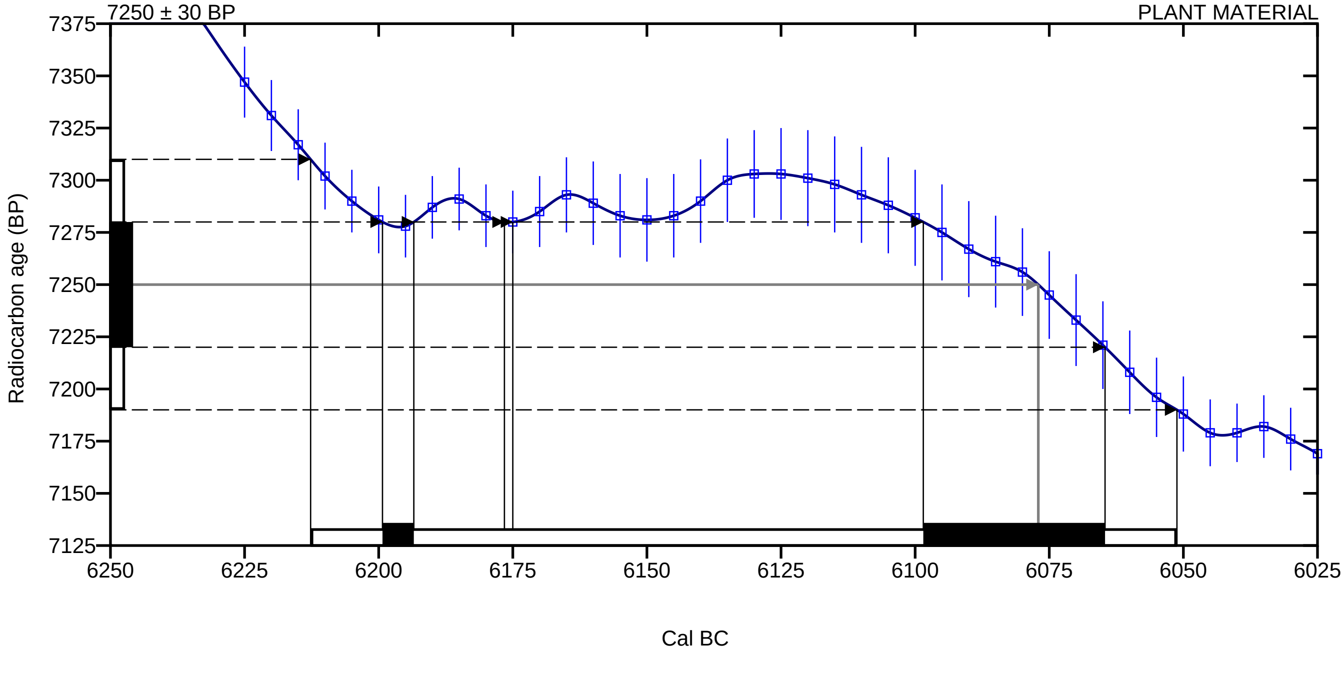

Database used

INTCAL13

References

Mathematics used for calibration scenario

A Simplified Approach to Calibrating C14 Dates, Talma, A. S., Vogel, J. C., 1993, Radiocarbon 35(2):317-322

References to INTCAL13 database

Reimer PJ et al. IntCal13 and Marine13 radiocarbon age calibration curves 0–50,000 years cal BP. Radiocarbon 55(4):1869–1887., 2013.

CALIBRATION OF RADIOCARBON AGE TO CALENDAR YEARS

(Variables: C13/C12 = -28.5 o/oo : lab. mult = 1)

**Laboratory number**	**Beta-422669 : FB48-21**
**Conventional radiocarbon age**	**7350 ± 30 BP**
**Calibrated Result (95% Probability)**	**Cal BC 6250 to 6205 (Cal BP 8200 to 8155)**<br>**Cal BC 6190 to 6185 (Cal BP 8140 to 8135)**<br>**Cal BC 6165 to 6160 (Cal BP 8115 to 8110)**<br>**Cal BC 6140 to 6105 (Cal BP 8090 to 8055)**
Intercept of radiocarbon age with calibration curve	Cal BC 6225 (Cal BP 8175)
Calibrated Result (68% Probability)	Cal BC 6235 to 6215 (Cal BP 8185 to 8165)

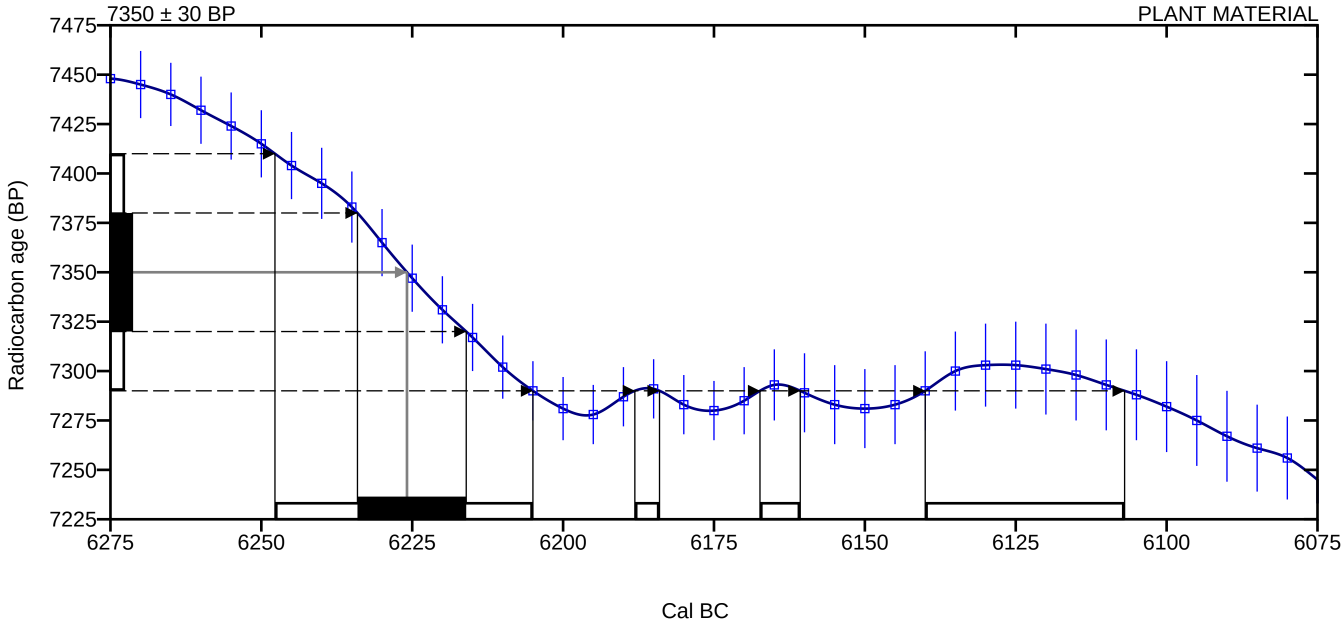

Database used

INTCAL13

References

Mathematics used for calibration scenario

A Simplified Approach to Calibrating C14 Dates, Talma, A. S., Vogel, J. C., 1993, Radiocarbon 35(2):317-322

References to INTCAL13 database

Reimer PJ et al. IntCal13 and Marine13 radiocarbon age calibration curves 0–50,000 years cal BP. Radiocarbon 55(4):1869–1887., 2013.

Beta Analytic Radiocarbon Dating Laboratory

4985 S.W. 74th Court, Miami, Florida 33155 • Tel: (305)667-5167 • Fax: (305)663-0964 • Email: beta@radiocarbon.com

CALIBRATION OF RADIOCARBON AGE TO CALENDAR YEARS

(Variables: C13/C12 = -27.6 o/oo : lab. mult = 1)

**Laboratory number**	**Beta-422670 : FB48-37**
**Conventional radiocarbon age**	**9210 ± 30 BP**
**Calibrated Result (95% Probability)**	**Cal BC 8545 to 8505 (Cal BP 10495 to 10455)**<br>**Cal BC 8490 to 8305 (Cal BP 10440 to 10255)**
Intercept of radiocarbon age with calibration curve	Cal BC 8435 (Cal BP 10385)<br>Cal BC 8365 (Cal BP 10315)<br>Cal BC 8350 (Cal BP 10300)
Calibrated Result (68% Probability)	Cal BC 8465 to 8330 (Cal BP 10415 to 10280)

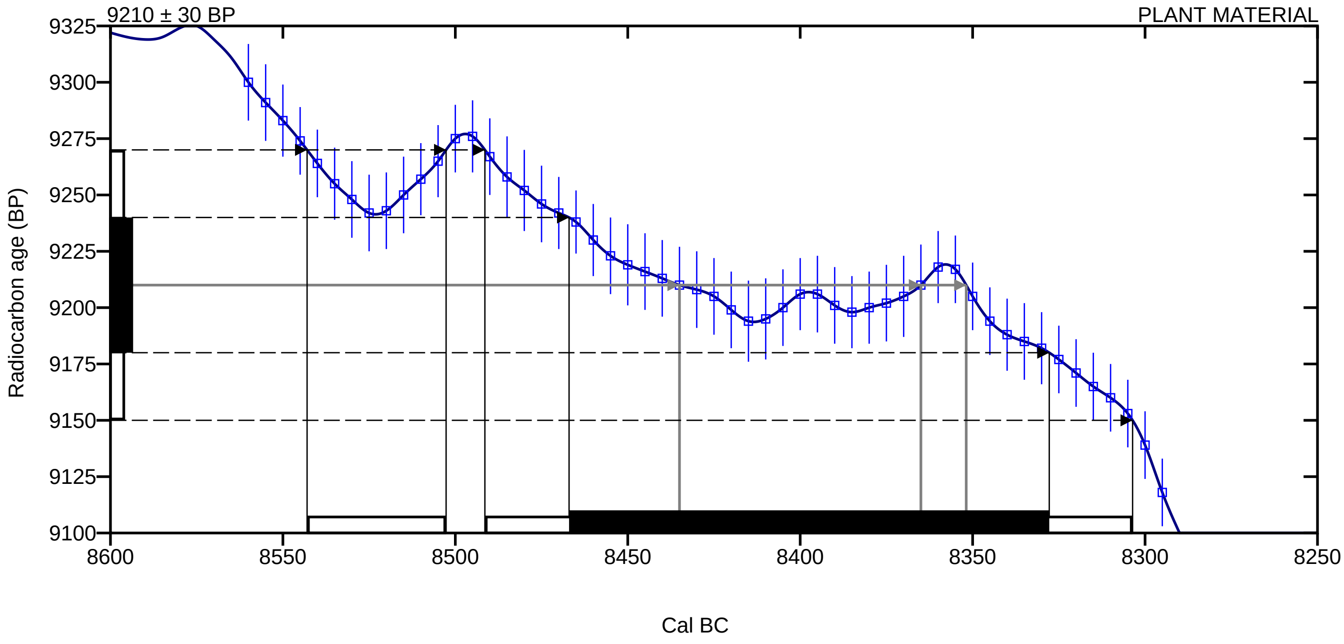

Database used

INTCAL13

References

Mathematics used for calibration scenario

A Simplified Approach to Calibrating C14 Dates, Talma, A. S., Vogel, J. C., 1993, Radiocarbon 35(2):317-322

References to INTCAL13 database

Reimer PJ et al. IntCal13 and Marine13 radiocarbon age calibration curves 0–50,000 years cal BP. Radiocarbon 55(4):1869–1887., 2013.

CALIBRATION OF RADIOCARBON AGE TO CALENDAR YEARS

(Variables: C13/C12 = -24.4 o/oo : lab. mult = 1)

Laboratory number **Beta-422672 : S3-C2-21.0**

Conventional radiocarbon age **5570 ± 30 BP**

Calibrated Result (95% Probability) **Cal BC 4455 to 4345 (Cal BP 6405 to 6295)**

Intercept of radiocarbon age with calibration curve
Cal BC 4440 (Cal BP 6390)
Cal BC 4425 (Cal BP 6375)
Cal BC 4370 (Cal BP 6320)

Calibrated Result (68% Probability) Cal BC 4450 to 4355 (Cal BP 6400 to 6305)

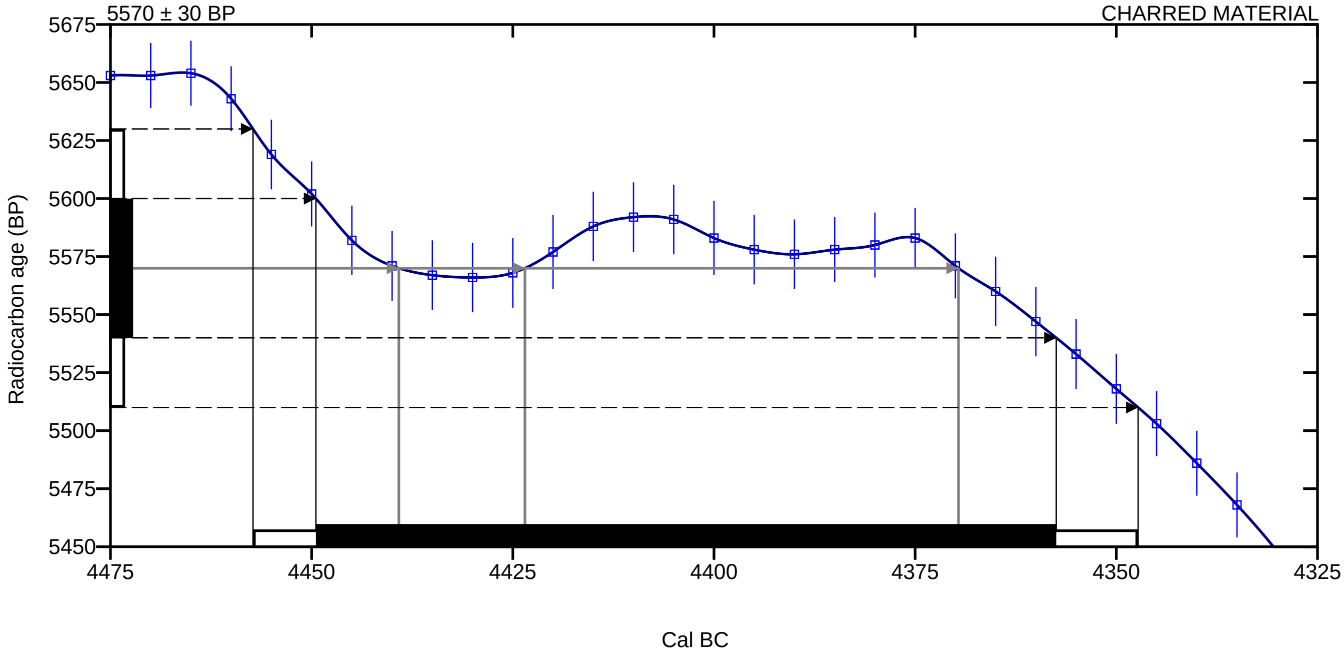

Database used

INTCAL13

References

Mathematics used for calibration scenario

A Simplified Approach to Calibrating C14 Dates, Talma, A. S., Vogel, J. C., 1993, Radiocarbon 35(2):317-322

References to INTCAL13 database

Reimer PJ et al. IntCal13 and Marine13 radiocarbon age calibration curves 0–50,000 years cal BP. Radiocarbon 55(4):1869–1887., 2013.

CALIBRATION OF RADIOCARBON AGE TO CALENDAR YEARS

(Variables: C13/C12 = -26.9 o/oo : lab. mult = 1)

**Laboratory number**	**Beta-422673 : S3-C5-22.8**
**Conventional radiocarbon age**	**4580 ± 30 BP**
**Calibrated Result (95% Probability)**	**Cal BC 3490 to 3470 (Cal BP 5440 to 5420)**<br>**Cal BC 3370 to 3335 (Cal BP 5320 to 5285)**<br>**Cal BC 3210 to 3190 (Cal BP 5160 to 5140)**<br>**Cal BC 3150 to 3140 (Cal BP 5100 to 5090)**
Intercept of radiocarbon age with calibration curve	Cal BC 3360 (Cal BP 5310)
Calibrated Result (68% Probability)	Cal BC 3365 to 3350 (Cal BP 5315 to 5300)

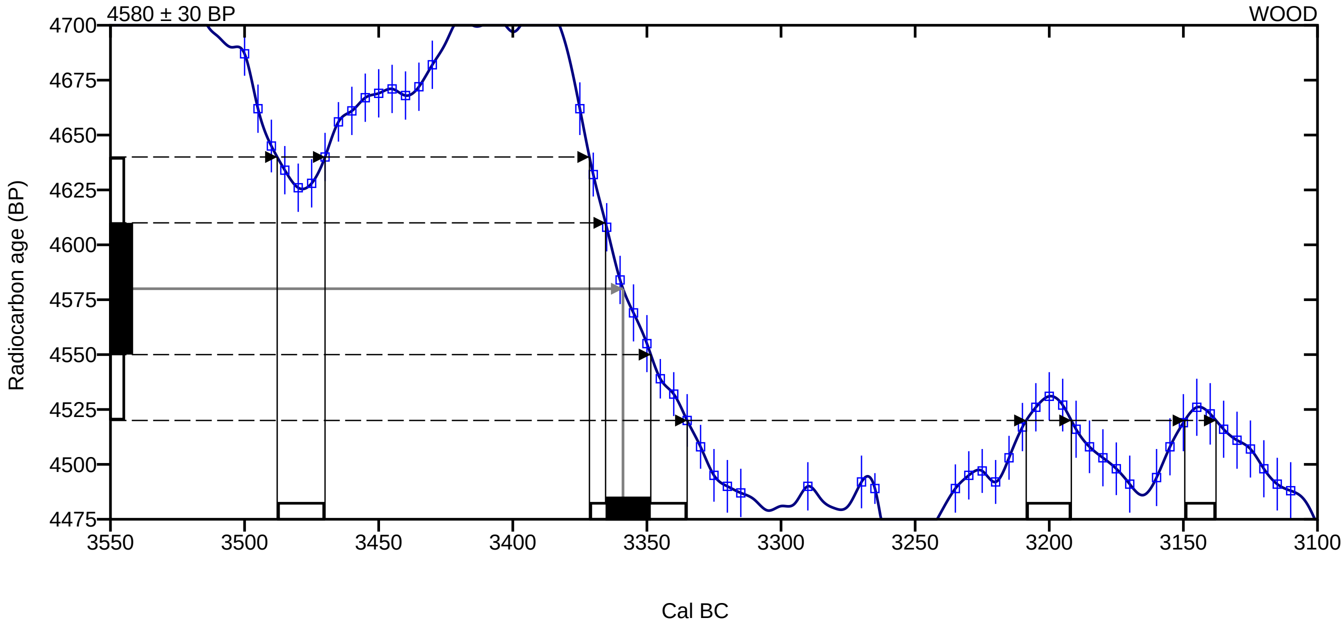

Database used

INTCAL13

References

Mathematics used for calibration scenario

A Simplified Approach to Calibrating C14 Dates, Talma, A. S., Vogel, J. C., 1993, Radiocarbon 35(2):317-322

References to INTCAL13 database

Reimer PJ et al. IntCal13 and Marine13 radiocarbon age calibration curves 0– 50,000 years cal BP. Radiocarbon 55(4):1869– 1887., 2013.
