# Supplementary material for: Rome in its setting. Post-glacial aggradation history of the Tiber River alluvial deposits and tectonic origin of the Tiber Island
Source: PLoS One. 2018 Mar 28;13(3):e0194838. doi: 10.1371/journal.pone.0194838 (PMC5874049; doi:10.1371/journal.pone.0194838)
Supplement: S4 File — (DOC) [file pone.0194838.s004.doc]

Rome, February 4th, 2018

to: Fabrizio Marra

Primo Ricercatore

Istituto Nazionale di Geofisica e Vulcanologia

Via di Vigna Murata 605, 00143, Roma

tel. 06 51860420

fax 06 51860507

fabrizio.marra@ingv.it

Dear Dr. Marra

you have my permission to use and modify if necessary the Digital Elevation

Model (DEM) for the region of Rome (TINITALY/01 square WA 6570) for

publication in Plos One.

Dr. Fabio Florindo

Director, Environment Department

Istituto Nazionale di Geofisica e Vulcanologia

Rome - Italy

fabio.florindo@ingv.it
